# Effect of Polyphenol-Rich Foods, Juices, and Concentrates on Recovery from Exercise Induced Muscle Damage: A Systematic Review and Meta-Analysis

**DOI:** 10.3390/nu13092988

**Published:** 2021-08-27

**Authors:** Lee Rickards, Anthony Lynn, Deborah Harrop, Margo E. Barker, Mark Russell, Mayur K. Ranchordas

**Affiliations:** 1 Academy of Sport & Physical Activity, Sheffield Hallam University, Sheffield S10 2BP, UK; lr7134@exchange.shu.ac.uk (L.R.); itsdh1@exchange.shu.ac.uk (D.H.); 2 Department of Service Sector, Management Business School, Sheffield Hallam University, Sheffield S1 1WP, UK; T.lynn@shu.ac.uk (A.L.); sbsmb@exchange.shu.ac.uk (M.E.B.); 3 School of Social and Health Sciences, Leeds Trinity University, Leeds LS18 5HD, UK; m.russell@leedstrinity.ac.uk

**Keywords:** polyphenols, muscle damage, recovery, supplementation, exercise

## Abstract

**Objectives.** To determine the effects of consuming polyphenol-rich foods, juices and concentrates on recovery from exercise-induced muscle damage (EIMD). **Method. Eligibility criteria.** Randomised and quasi-randomised placebo-controlled trials with a parallel or cross-over design evaluating the effects of consuming polyphenol-rich foods, juices and concentrates on recovery from EIMD in humans. Eligible studies included at least one of the primary outcome measures: maximal isometric voluntary contraction; MIVC, delayed onset muscle soreness; DOMS, or countermovement jump; CMJ. **Information sources.** AMED, Cochrane Central Register of Controlled Trials, International Clinical Trials Registry Platform, PUBMED, SCOPUS (Elsevier), SPORTDiscus (EBSCO), and the UK Clinical Trials Gateway were searched from inception to September 2020. **Risk of bias and quality of evidence.** Risk of bias was assessed using Cochrane Risk of Bias 2 tool. Quality of the evidence was assessed using the Grading of Recommendations, Assessment, Development and Evaluation framework. **Synthesis of results.** Random effects models were used to determine the effect of polyphenol supplementation on recovery from EIMD. Data are presented as standardised mean differences (SMD) with 95% confidence intervals (CI). **Results. Included studies.** Twenty-five studies were included; 15 had a parallel, and 10 had a cross-over design. A total of 527 participants (male: *n* = 425; female: *n* = 102) were included in the meta-analysis. **Synthesis of results.** Consumption of polyphenol-rich foods, juices and concentrates accelerated recovery of MIVC immediately post-exercise (SMD = 0.23, 95% CI 0.04, 0.42; *p* = 0.02; low-quality evidence), 24 h (SMD = 0.39, 95% CI 0.15, 0.62; *p* = 0.001; low-quality evidence), 48 h (SMD = 0.48, 95% CI 0.28, 0.67; *p* < 0.001; moderate-quality evidence), 72 h (SMD = 0.29, 95% CI 0.11, 0.46; *p* = 0.001; low-quality evidence) and 96 h post-exercise (SMD = 0.50, 95% CI 0.16, 0.83; *p* = 0.004; very low-quality evidence). DOMS was reduced at 24 h (SMD = −0.29, 95% CI −0.47, −0.11; *p* = 0.002; low-quality evidence), 48 h (SMD = −0.28, 95% CI −0.46, −0.09; *p* = 0.003; low-quality evidence) and 72 h post-exercise (SMD = −0.46, 95% CI −0.69, −0.24; *p* < 0.001; very low-quality evidence). CMJ height was greater immediately post-exercise (SMD = 0.27, 95% CI 0.01, 0.53; *p* = 0.04; low-quality evidence), at 24 h (SMD = 0.47, 95% CI 0.11, 0.83; *p* = 0.01; very low-quality evidence), 48 h (SMD = 0.58, 95% CI 0.24, 0.91; *p* < 0.001; very low-quality evidence) and 72 h post-exercise (SMD = 0.57, 95% CI 0.03, 1.10; *p* = 0.04; very low-quality evidence). Polyphenol supplementation did not alter creatine kinase, c-reactive protein, and interleukin−6 at any time points. At 72 h post-exercise, protein carbonyls (SMD = −0.64, 95% CI −1.14, −0.14; *p* = 0.01) were reduced. **Discussion. Limitations of evidence.** Risk of bias was high for 10 studies and moderate for 15. Sensitivity analyses excluding the high risk of bias studies reduced the SMDs for MIVC and DOMS, and for CMJ effects at 24 and 48 h were no longer statistically significant. **Interpretation.** Consuming polyphenol-rich foods, juices and concentrates accelerated recovery of muscle function while reducing muscle soreness in humans. Maximal benefit occurred 48–72 h post-exercise, however, the certainty of the evidence was moderate to very low. Supplementation could be useful when there is limited time between competitive events and impaired recovery could negatively impact performance.

## 1. Introduction

Exercise of a high intensity and/or duration, especially with an eccentric component, can induce muscle damage [1,2]. Exercise-induced muscle damage (EIMD) is characterised by impaired force production, increased muscle soreness and reduced range of motion [1,3]. These symptoms can impair subsequent performance. Therefore, sport nutrition strategies often aim to attenuate EIMD and accelerate recovery to enhance readiness to play or train. This is especially important when recovery time is reduced, such as during tournaments, multi-day events or periods of competition congestion.

The exact mechanisms underpinning EIMD have yet to be fully elucidated, but a two phase process has been proposed [4]. Firstly, mechanical damage to sarcomeres may cause overstretching of some filaments (sarcomere ‘popping’) [5] resulting in Z-band streaming and a loss of force production [6]. Secondly, an acute inflammatory response and disruption of redox balance may further damage the muscle [7]. Because inflammation and disruption of redox balance are implicated in the secondary phase of muscle damage, a growing number of studies have investigated whether foods with antioxidant and anti-inflammatory properties accelerate recovery. 

Polyphenols are secondary plant metabolites attributed with antioxidant and anti-inflammatory properties [8]. Connolly et al. [9] were the first to demonstrate that a polyphenol-rich tart cherry/apple juice blend could accelerate recovery from eccentric elbow flexion. Accordingly, studies have subsequently investigated the efficacy of a large number of other foods and extracts rich in polyphenols including pomegranate [10], bilberry [11], blueberry [12], beetroot [13] and cocoa [14]. The results of these studies have been inconsistent, potentially due to methodological variation, including differences in exercise protocols, intervention periods, outcome markers, dosages, and the polyphenol content and bioactive constituents of the supplements consumed (e.g., beetroot is rich in nitrate, betalains and polyphenols) [15,16].

To our knowledge, only one meta-analysis has synthesised the evidence on fruit-derived polyphenols and recovery from EIMD [17]. Supplementation elicited a faster recovery of maximal isometric voluntary contraction (MIVC), increased antioxidant capacity and reduced markers of muscle damage, inflammation, and oxidative stress. However, this meta-analysis only reviewed evidence at 24 and 48 h post exercise, even though many of the included studies measured outcomes beyond this point and the symptoms of EIMD often take longer than 48 h to resolve. It is also difficult to interpret the results of Doma et al. [17] for blood markers of recovery because they pooled results from different biochemical assays that are not directly comparable.

Doma et al. [17] investigated the effects of polyphenol-rich food and isolated extracts in their meta-analysis. However, we recently found that in elite football, practitioners preferred to recommend polyphenol-rich fruits and vegetables rather than supplements of isolated compounds or extracts (manuscript in preparation). As different foods have distinct polyphenolic profiles it is important to explore their individual efficacy. Doma et al. [17] did not address this issue in their review nor did they evaluate the effects of restricting polyphenols in the background diet which could also confound the results of studies. Given these limitations, the main aim of this meta-analysis was to assess the effects of polyphenol-rich fruits and vegetables on recovery of EIMD up to 96 h post-exercise. The primary outcome markers chosen to assess recovery in this meta-analysis were MIVC, countermovement jump (CMJ) and delayed onset muscle soreness (DOMS). MIVC and CMJ both measure muscle force which has been argued to be the most appropriate marker of muscle damage [18]. Whereas DOMS is a commonly used marker of exercise-induced muscle pain [18]. The secondary outcomes assessed were blood markers of muscle damage (creatine kinase), inflammation (c-reactive protein, interleukin-6), and oxidative stress (protein carbonyls). These were selected because they have been commonly used in the literature to measure the extent of muscle damage and/or recovery from the secondary phase of EIMD [3].

Additional aims of this review were to explore differences in the effects of individual polyphenol-rich fruit/vegetable products on recovery from EIMD and to investigate whether restricting polyphenols in the background diet influences the efficacy of supplementation.

## 2. Materials and Methods

The protocol for this review was registered on the International Prospective Register of Systematic Reviews (PROSPERO) (CRD42018097123; 4 June 2018) and undertaken according to the guidelines of the Preferred Reporting Items for Systematic Reviews and Meta- Analysis (PRIMSA) [19] and the Cochrane handbook [20]. The original protocol specified this review was limited to studies with a parallel design because in cross-over designs susceptibility to muscle damage might be reduced on second exposure to eccentric exercise, even in the contralateral limb, a phenomenon known as the repeated bout effect [21]. Acknowledging that many studies in this area used a cross-over design, we have presented results for each study design independently and then overall as a combined result. Studies on isolated polyphenols/extracts were excluded because prior work conducted with sport nutrition practitioners indicated that >70% preferred to recommend whole foods rather than extracts and isolated polyphenols (manuscript in preparation). 

### 2.1. Eligibility Criteria 

We included randomised and quasi-randomised controlled trials with a parallel or cross-over design published in English. Included studies compared a polyphenol-rich food, juice or concentrate with a placebo on recovery from EIMD. Participants were aged > 16 years, male or female, trained or untrained, and free from chronic disease. We included studies that: (1) used an exercise protocol designed to induce muscle damage; (2) investigated at least one of the following outcomes post-exercise: MIVC, DOMS or CMJ height. Studies were excluded if: (1) the polyphenol was not provided as a food, juice or concentrate; (2) polyphenol foods were combined with other supplements; (3) there was no control for practices that could have influenced recovery (e.g., simultaneous or additive use of compression garments, cold water immersion, other analgesic medication etc.).

### 2.2. Search Strategy

Studies that investigated the effects of polyphenols on recovery were identified by searching in the following databases from inception to September 2020: AMED, Cochrane Central Register of Controlled Trials, International Clinical Trials Registry Platform, PUBMED, SCOPUS (Elsevier), SPORTDiscus (EBSCO), and the UK Clinical Trials Gateway for ongoing, discontinued and completed studies. In addition, we performed citation chaining using identified studies to find other relevant publications. Details of our full search strategy is shown in online Supplementary File 1. 

### 2.3. Study Selection 

Eligibility of the studies was performed by two independent reviewers (LR and MKR) in a standardised manner. Titles, abstracts, and descriptors of the trials retrieved from the searches were independently screened. Studies that satisfied the inclusion criteria were selected and the full text reviewed. In the case of disagreements a third reviewer (AL) was consulted. 

### 2.4. Data Extraction 

Two reviewers (LR and MKR) independently extracted data from the included studies using a pre-piloted data extraction form. Disagreements were resolved by discussion and consultation with a third reviewer (AL). When necessary, we contacted authors for additional information and data not reported in their manuscript. When data were not available in the manuscripts and authors did not respond to our contact requests, data were extracted from graphs in the published manuscript using *Origin Pro 2020b* (Northampton, MA, USA, 2020), where possible. Information was extracted from each included study on: (1) characteristics of study participants (including age, sex, physical activity level); (2) type of intervention (including type, dosage, duration, and frequency of polyphenol supplementation); (3) means and standard deviations (SD) for each outcome measure (MIVC, DOMS, CMJ, creatine kinase; CK, C-reactive protein; CRP, interleukin-6; IL-6, and protein carbonyls; PC). Where standard errors were reported, we converted these to SDs using Review Manager [22].

### 2.5. Risk of Bias in Individual Studies

Included studies were independently assessed for risk of bias by two reviewers (AL and MKR). Studies with a parallel design were assessed using the Risk of Bias 2 tool (ROB 2) [23] and this was adapted for cross-over studies using guidelines provided by Higgins et al. [24]. Each study was assessed for quality across the following categories: (1) randomisation process; (2) deviations from intended interventions; (3) missing outcome data; (4) measurement of the outcome; (5) selection of the reported result. The overall assessment comprised of three ratings: low risk of bias, some concerns or high risk of bias. We resolved any disagreement by consulting a third reviewer (LR). Publication bias was investigated by visually inspecting funnel plots for asymmetry (see Supplementary File 2).

### 2.6. Data Synthesis 

Meta-analyses were conducted using Review Manager [22]. We converted the data to SMDs for all primary and secondary outcomes because of differences in the units of measurement reported across studies. For studies with a parallel design, extracted means, SDs and number of participants were inputted into Review Manager to calculate the SMDs. The computed 95% CIs were used to calculate standard errors of the SMDs using the following equation [20]:

$$\frac{Upper\;95\%\;CI-Lower\;95\%\;CI}{3.92}$$

For studies with a cross-over design, the extracted means, SDs and number of participants were used to calculate the SD pooled, SMD, and standard error of the SMD using the following equations [20]:

$$SMD=\frac{MD}{SD_{pooled}}$$

$$SD_{pooled}=\sqrt{\frac{SD_{E}^{2}+SD_{C}^{2}\;}{2}}$$

$$SE\left(SMD\right)=\sqrt{\frac{1}{N}+\frac{SMD^{2}\;}{2N}}\times \sqrt{2\left(1-Corr\right)}$$

When the raw data were available from cross-over trials we calculated the correlation between repeated measures. If no data were available we used a conservative correlation of 0.5 [20]. Some studies reported multiple measures of primary outcome markers (e.g., DOMS or MIVC at several anatomical sites [10,25,26]; or compared more than one treatment to a single placebo group [25,27,28]. To avoid these studies contributing more than one set of data (causing a unit of analysis error), we calculated combined means and SDs to produce a single SMD for each outcome marker at each time point per study [20,29]. Sensitivity analyses were conducted by removing the studies classified as high risk of bias to assess the robustness of treatment effects.

The polyphenol-rich foods included in this review differ in their content of polyphenols and other compounds that might promote recovery. Therefore, when two or more studies on an individual polyphenol-rich food were available, we conducted separate meta-analyses for the primary outcomes. To test whether polyphenol-rich foods were statistically different from each other we used the test for subgroup differences available in RevMan [22]. The efficacy of polyphenol supplementation to accelerate recovery may be influenced by the quantity of polyphenols in the background diet of participants. Therefore, we conducted subgroup analyses for our primary outcomes to compare studies that restricted the quantity of polyphenols in the background diet with those that did not.

SMDs were interpreted using the classifications of 0.2, 0.5 and 0.8 as small, moderate, and large effects, respectively [30]. Statistical significance was set at *p* ≤ 0.05. To aid the interpretation of the SMDs of our primary outcome markers, effect sizes were converted into percentages. First the SMDs were converted into mean differences (MDs) by multiplying them by estimates of the SDs associated with the most frequently used unit of measurement (MFU) (MIVC: N, DOMS: cm and CMJ height: cm) [31]. Second, these calculated MDs were converted into percentages:

$$\frac{MD}{Mean\;of\;MFU\;placebo\;group}\;\times \;100$$

We assessed heterogeneity of data by visually inspecting forest plots, and conducting the I^2^ test and χ^2^ test. We considered an I^2^ value greater than 40% and/or a χ^2^ *p* value of less than 0.1 as evidence of substantial heterogeneity [20]. For a number of outcome markers there was substantial heterogeneity for one or more time points, therefore, we used the random effects model for all of the analyses. When applied to homogeneous studies, the random effects model produces the same estimate of effect as the fixed effect model [32].

### 2.7. Quality Assessment 

Overall quality of evidence was assessed by two independent reviewers (AL and MKR) using the Grading of Recommendations, Assessment, Development and Evaluation framework (GRADE) [33]. Any disagreements were resolved by consulting a third reviewer (LR). Overall quality of evidence for each primary outcome was rated from high to very low. Quality was assessed against the following five factors: (1) study limitations; (2) imprecision; (3) inconsistency of results; (4) indirectness of evidence; (5) publication bias (see Supplementary File 3).

## 3. Results

### 3.1. Study Selection 

**Figure 1:**
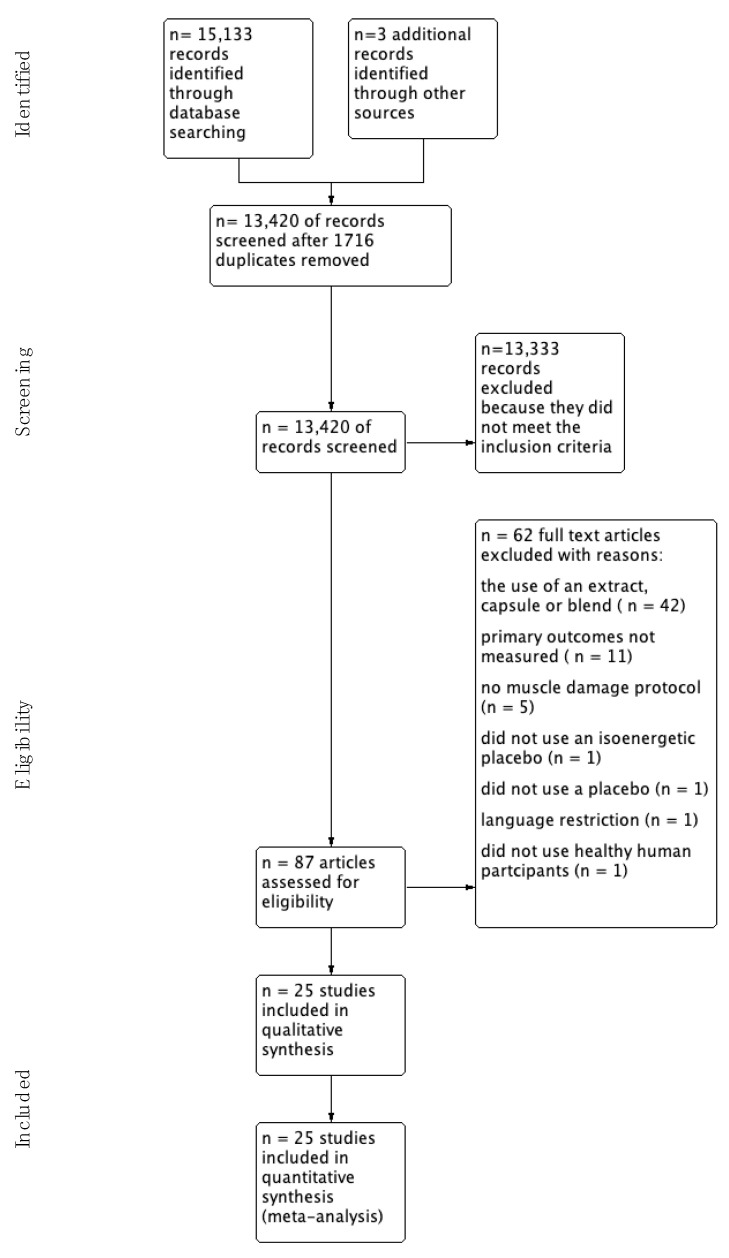
Study Flow Diagram.

A total of 25 studies were identified for inclusion in this review. The search of AMED, Cochrane Central Register of Controlled Trials, International Clinical Trials Registry, PUBMED, SCOPUS (Elsevier), SPORTDiscus (EBSCO), and UK Clinical Trials Gateway identified a total of 15,133 records and a further three studies were identified through citation chaining. Of these, 13,420 were screened after removing 1716 duplicates. After reviewing the abstracts 13,333 were removed because they did not meet our inclusion criteria. The full text of the remaining 87 studies were examined in more detail and 62 were removed (see flow diagram for reasons Figure 1). 

### 3.2. Study Characteristics 

#### 3.2.1. Methods

Of the 25 studies included in the review, 15 had a parallel design and 10 employed a cross-over design (see Table 1). Participants were randomised to treatment groups in 20 studies, whereas, a quasi-randomisation protocol was used in one cross-over [34] and four parallel studies [27,35,36,37]. In 18 studies both investigators and participants were blinded to the treatment allocation [10,13,14,25,27,28,34,35,37,38,39,40,41,42,43,44,45,46], in 4 studies investigators were aware of the treatment allocation [11,26,47,48] and in three studies there was no/unclear information on blinding [9,12,36]. The duration of supplementation ranged from 1 to 15 days.

#### 3.2.2. Participants

A total of 527 participants (male *n* = 425; female *n* = 102) were included in this meta-analysis. Of the 527 participants, 29 (5.5%) were semi-professional athletes [40,43], 31 (5.8%) were professional athletes [38,45,47] and 467 (88.6%) participants were recreationally trained.

#### 3.2.3. Intervention

All of the included studies compared a polyphenol-rich food, juice or concentrate to a placebo as follows:

- cherry (*n* = 12) * [9,28,34,36,37,38,39,40,41,45,47,48]
- pomegranate (*n* = 3) * [10,25,28]
- beetroot (*n* = 5) [13,27,35,42,43]
- cocoa (*n* = 2) [14,26]
- bilberry (*n* = 1) [11]
- blueberry (*n* = 1) [12]
- blackcurrant (*n* = 1) [44]
- mixed fruit juice (*n* = 1) [46]

* One study included cherry and pomegranate [28]. 

Trials were conducted in the UK (*n* = 16), the USA (*n* = 6), New Zealand (*n* = 1), Tunisia (*n* = 1), and Brazil (*n* = 1).

#### 3.2.4. Adverse Events

Only two of 25 studies asked participants about adverse responses to the supplements [11,12]; both reported no adverse events.

### 3.3. Outcomes 

#### 3.3.1. Primary 

Of the 25 studies included in this review, 19 reported on MIVC, 24 reported a measure of muscle soreness, and 11 reported on CMJ (see Table 1). There was, however, variability between the studies in the number of time points measured ranging from immediately post-exercise to 96 h post-exercise. 

#### 3.3.2. Secondary

Of the 25 studies included in this review, 18 reported on CK, 12 on CRP, eight on IL-6 and five measured PC (see Table 1). There was substantial variation in the number of post-exercise time points measured by each individual study. CK and CRP were measured up to 96 h post-exercise in some studies, however, IL-6 and PC were only measured for a maximum of 72 h post-exercise.

### 3.4. Risk of Bias within Studies 

**Figure 2:**
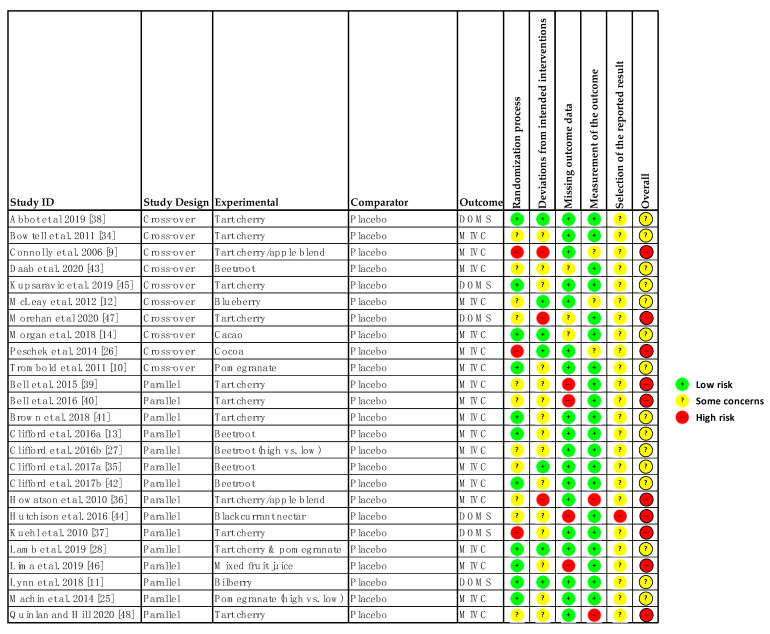
Risk of Bias Tool 2 [23]. MIVC: Maximal isometric voluntary contraction; DOMS: Delayed onset muscle soreness.

Of the 25 studies, 15 were rated as some concerns and 10 as high risk of bias (see Figure 2). Factors driving an overall high risk of bias were lack of information in the manuscript, failure to blind assessors, issues with randomisation, and lack of information on adherence to the intervention. An issue with the studies included in this review was that none pre-registered their study protocol so there was no way of confirming whether they had a pre-specified data analysis plan [24]. Visual inspection of the funnel plots (see Supplementary File 2) did not identify substantial asymmetry providing little evidence of publication bias. 

### 3.5. Syntheses of Results 

#### 3.5.1. Primary Outcomes

##### MIVC

**Figure 3:**
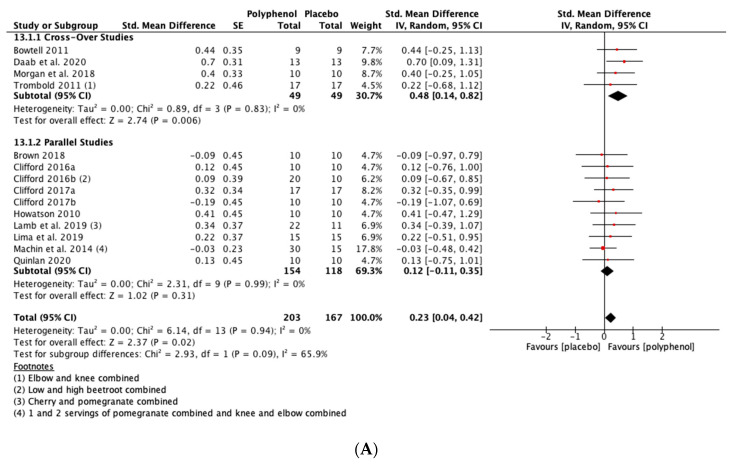

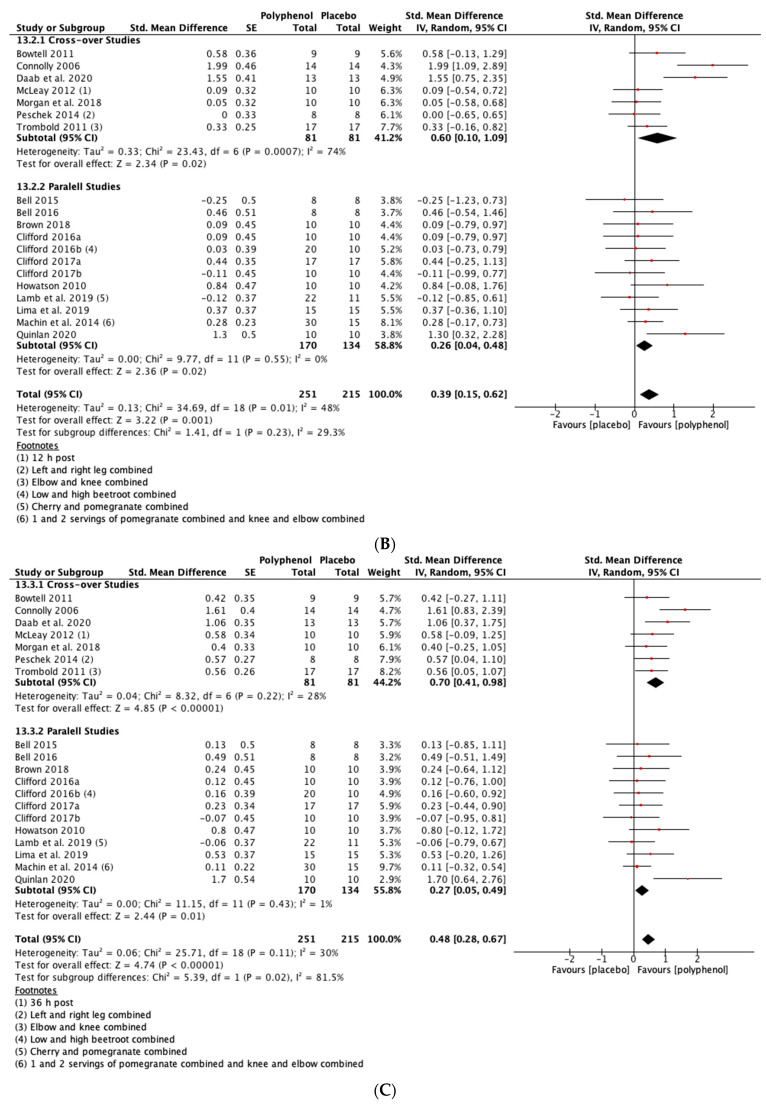

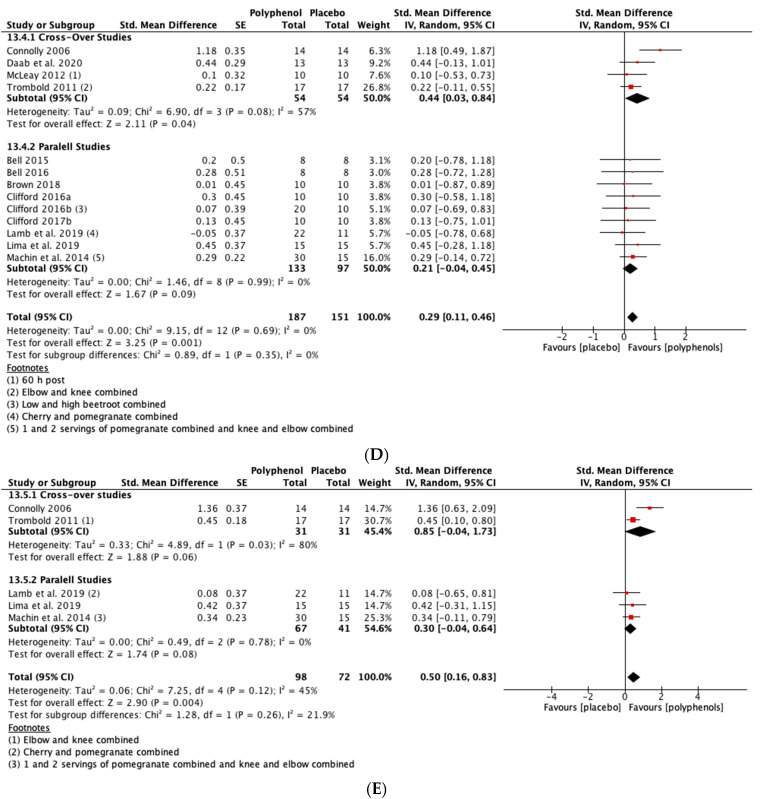
Effect of polyphenol-rich foods, juices and concentrates on recovery of maximal voluntary isometric contraction **A**) immediately post-exercise; (**B**) 24 h; (**C**) 48 h; (**D**) 72 h; (**E**) 96 h.

Polyphenol supplementation caused small but significant increases in MIVC in comparison to placebo immediately post-exercise (SMD = 0.23, 95% CI 0.04, 0.42; *p* = 0.02; participants = 370; studies = 14; I^2^ = 0%; low-quality evidence), 24 h post-exercise (SMD = 0.39, 95% CI 0.15, 0.62; *p* = 0.001; participants = 466; studies = 19; I^2^ = 48%; low-quality evidence), 48 h post-exercise (SMD = 0.48, 95% CI 0.28, 0.67; *p* < 0.001; participants = 466; studies = 19; I^2^ = 30%; moderate-quality evidence), 72 h post-exercise (SMD = 0.29, 95% CI 0.11, 0.46; *p* = 0.001; participants = 338; studies = 13; I^2^ = 0%; low-quality evidence) and at 96 h post-exercise (SMD = 0.50, 95% CI 0.16, 0.83; *p* = 0.004; participants = 170; studies = 5; I^2^ = 45%; very low-quality evidence). These SMDs equated to improvements in MIVC of 6.0% (immediately post), 7.7% (24 h), 9.6% (48 h), 5.7% (72 h) and 13.0% (96 h) (see Figure 3).

When the studies were separated by design, the effect sizes were larger in the cross-over studies than the parallel studies at all time points, but subgroup analyses only found a statistically significant difference at the 48 h time-point (*p* = 0.02; see Figure 3C). 

##### DOMS

**Figure 4:**
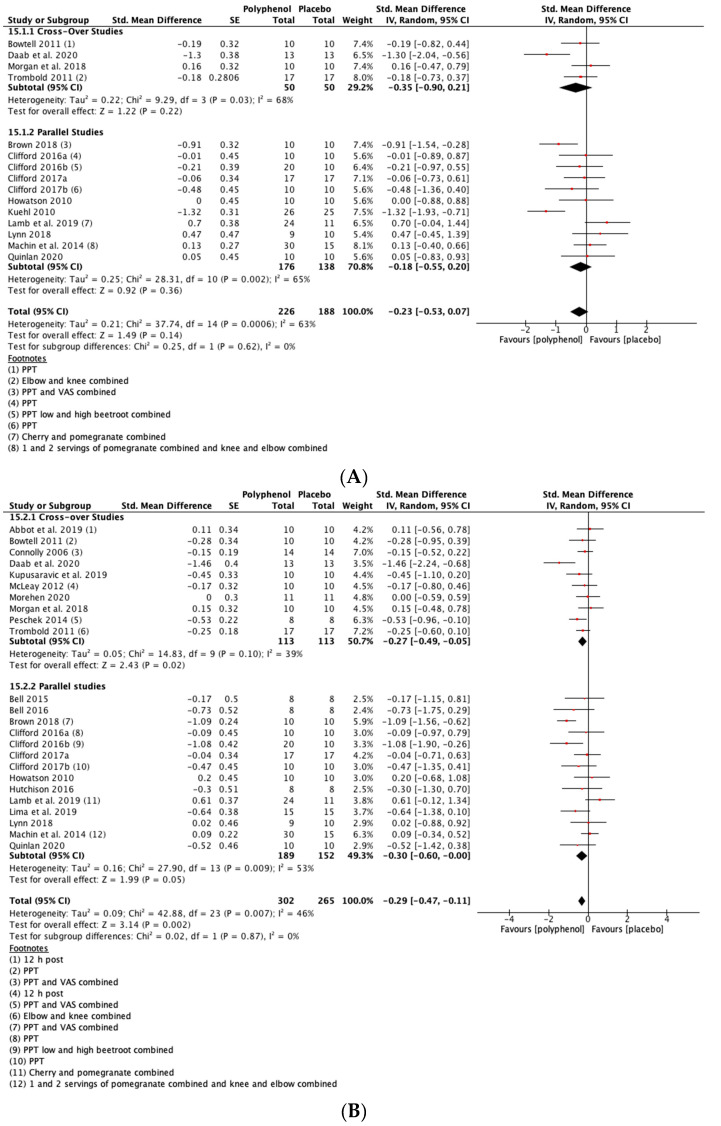

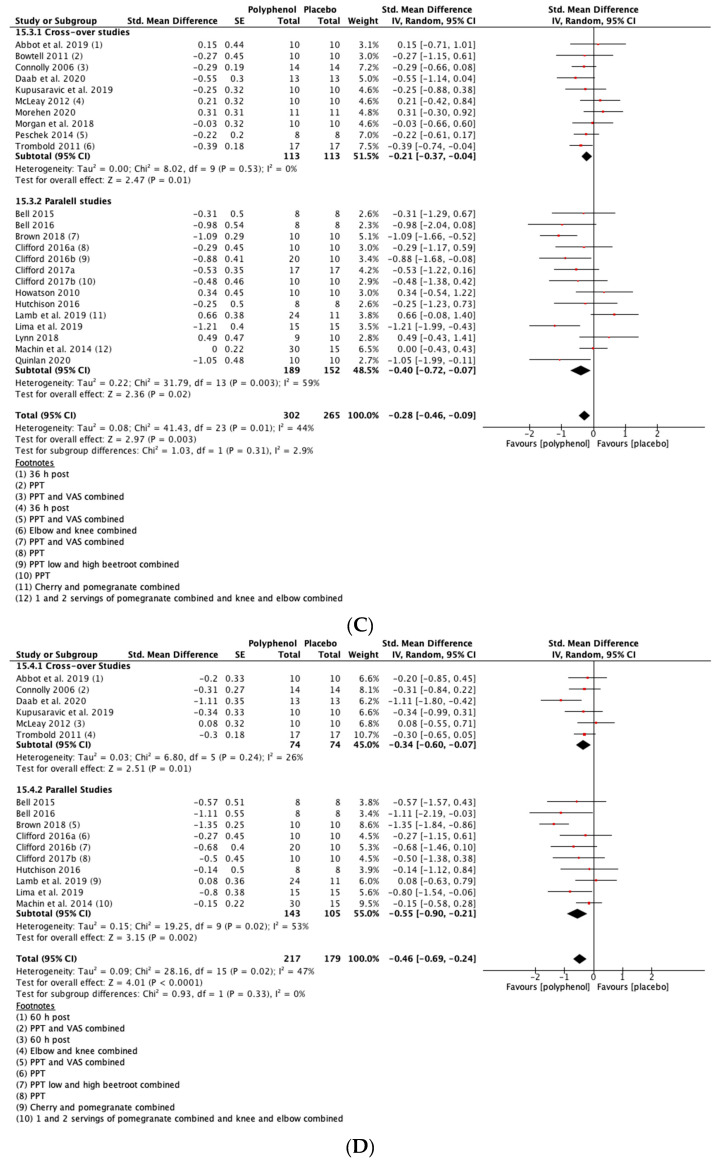

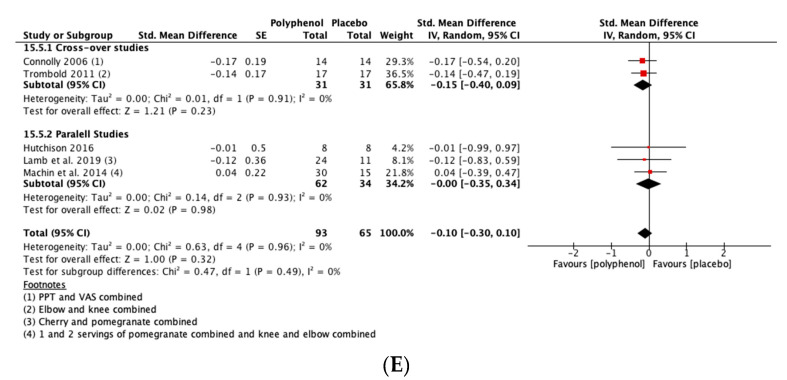
Effect of polyphenol-rich foods, juices and concentrates on recovery of delayed onset muscle soreness (**A**) immediately post-exercise; (**B**) 24 h; (C) 48 h; (**D**) 72 h; (**E**) 96 h. Unless stated otherwise SMDs are calculated from VAS of DOMS. PPT: Pain pressure threshold. VAS: Visual analogue scale.

There was a small but not statistically significant reduction in DOMS immediately post-exercise (SMD = −0.23, 95% CI −0.53, 0.07; *p* = 0.14; participants = 380; studies = 15; I^2^ = 63%; low-quality evidence). At 24 h post-exercise (SMD = −0.29, 95% CI −0.47, −0.11; *p* = 0.002; participants = 567; studies = 24; I^2^ = 46%; low-quality evidence), 48 h post-exercise (SMD = −0.28, 95% CI −0.46, −0.09; *p* = 0.003; participants = 567; studies = 24; I^2^ = 44%; low-quality evidence) and 72 h post-exercise (SMD = −0.46, 95% CI −0.69, −0.24; *p* < 0.001; participants = 396; studies = 16; I^2^ = 47%; very low-quality evidence) there were small but statistically significant reductions in DOMS in response to polyphenol supplementation. At 96 h, DOMS had returned towards baseline levels and there was no statistically significant difference between polyphenols and placebo (SMD = −0.10, 95% CI −0.30, 0.10; *p* = 0.32; participants = 158; studies = 5; I^2^ = 0%; low-quality evidence) (see Figure 4). These SMDs were equivalent to reductions in DOMS of 10.5% (immediately post), 9.4% (24 h), 10.3% (48 h), 29.3% (72 h) and 9.3% (96 h). Subgroup analyses did not detect any statistically significant differences in SMDs between cross-over and parallel studies at any post-exercise time point.

##### CMJ

**Figure 5:**
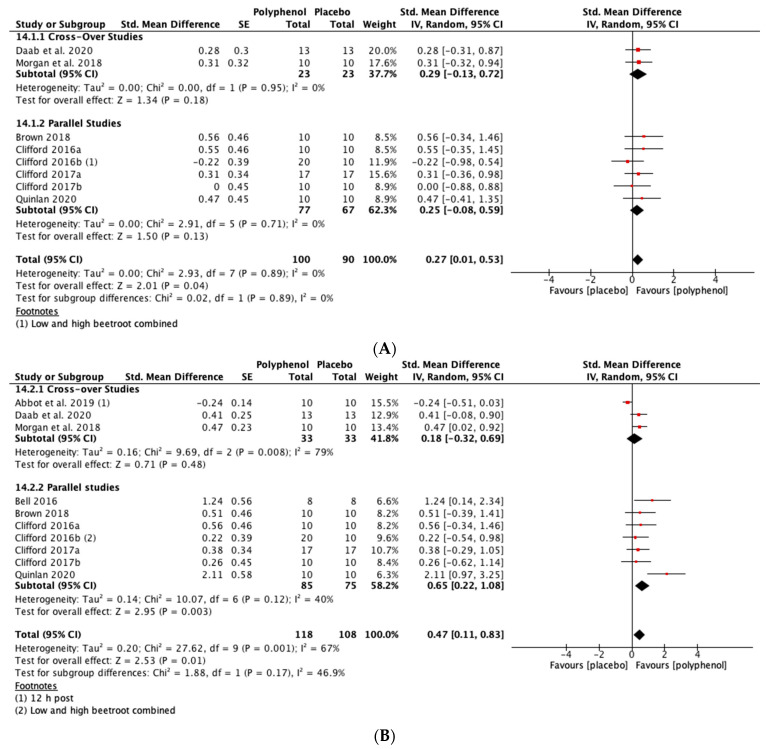

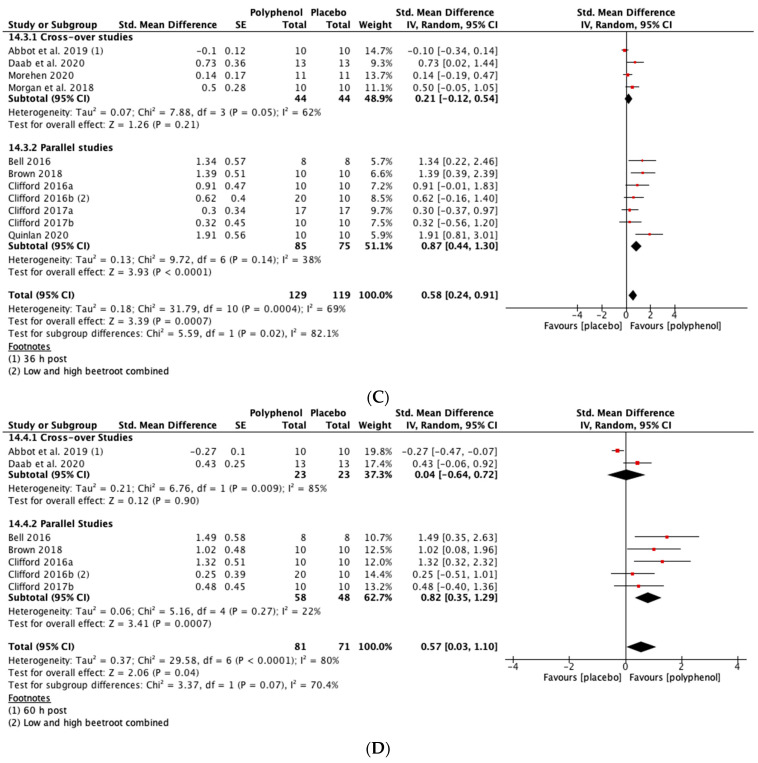
Effect of polyphenol-rich foods, juices and concentrates on recovery of countermovement jump height (**A**) immediately post-exercise; (**B**) 24 h; (**C**) 48 h; (**D**) 72 h.

Immediately post-exercise (SMD = 0.27, 95% CI 0.01, 0.53; *p* = 0.04; participants = 190; studies = 8; I^2^ = 0%; low-quality evidence) and at 24 h post-exercise (SMD = 0.47, 95% CI 0.11, 0.83; *p* = 0.01; participants = 226; studies = 10; I^2^ = 67%; very low-quality evidence) there were small statistically significant effects of polyphenol supplementation on CMJ height. At 48 h (SMD = 0.58, 95% CI 0.24, 0.91; *p* < 0.001; participants = 248; studies = 11; I^2^ = 69%; very low-quality evidence) and 72 h (SMD = 0.57, 95% CI 0.03, 1.10; *p* = 0.04; participants = 152; studies = 7; I^2^ = 80%; very low-quality evidence) post-exercise there were medium-sized effects of polyphenol supplementation on enhancing recovery of CMJ height (see Figure 5). These SMDs were equivalent to increases in CMJ height of 5.5% (immediately post), 9.9% (24 h), 13.1% (48 h) and 8.0% (72 h).

When the studies were separated by design, the SMDs were larger in the parallel studies at all time points except immediately post-exercise. However, subgroup analyses only revealed a statistically significant difference at 48 h (*p* = 0.02; see Figure 5C).

##### Heterogeneity 

We detected substantial heterogeneity at 24 h post-exercise for MIVC (I^2^ = 48%; χ^2^ = 34.69, df = 18; *p* = 0.01), immediately (I^2^ = 63%; χ^2^ = 37.74, df = 14; *p* < 0.001), 24 h (I^2^ = 46%; χ^2^ = 42.88, df = 23; *p* = 0.007), 48 h (I^2^ = 44%; χ^2^ = 41.43, df = 23; *p* = 0.01) and 72 h post-exercise (I^2^ = 47%; χ^2^ = 28.16, df = 15; *p* = 0.02) for DOMS, and at 24 h (I^2^ = 67%; χ^2^ = 27.62, df = 9; *p* = 0.001) and 48 h (I^2^ = 69%; χ^2^ = 31.79, df = 10; *p* < 0.001) post-exercise for CMJ height. Exploration of heterogeneity revealed that different studies were driving the substantial heterogeneity for each primary outcome. Exclusion of these studies removed the heterogeneity and attenuated the SMDs for DOMS and MIVC, but treatment effects remained statistically significant. For CMJ, removing studies causing substantial heterogeneity had almost no effect on the SMDs.

##### Sensitivity Analysis 

A sensitivity analysis excluding 10 studies classified as high risk of bias (see Supplementary File 4) attenuated the SMDs for MIVC, but the effects of polyphenol supplementation remained statistically significant. Removal of the high risk studies had little effect on the SMDs for DOMS. The SMDs for CMJ height were attenuated and no longer statistically significant immediately post-exercise (SMD = 0.25, 95% CI −0.03, 0.53; *p* = 0.08) and at the 24 (SMD = 0.25. 95% CI −0.03, 0.53; *p* = 0.08) and 72 h time-points (SMD = 0.44, 95% CI −0.09, 0.97; *p* = 0.10) (see Supplementary File 4). 

#### 3.5.2. Secondary Outcomes

##### CK

There were no statistically significant effects of polyphenol supplementation on CK immediately post-exercise (SMD = −0.05, 95% CI −0.35, 0.25; *p* = 0.75; participants = 315; studies = 14; I^2^ = 52%), 24 h post-exercise (SMD = 0.11, 95% CI −0.10, 0.32; *p* = 0.30; participants = 367; studies = 17; I^2^ = 27%), 48 h post-exercise (SMD = 0.07, 95% CI −0.19, 0.33; *p* = 0.62; participants = 397; studies = 18; I^2^ = 55%) and 72 h post-exercise (SMD = −0.14, 95% CI −0.52, 0.24; *p* = 0.47; participants = 202; studies = 9; I^2^ = 52%). Only three parallel studies measured CK at 96 h post-exercise; these revealed a moderate-sized effect in favour of polyphenol supplementation (SMD = −0.62, 95% CI −1.25, 0.00; *p* = 0.05; participants = 80; studies = 3; I^2^ = 41%) (see Supplementary File 5).

##### CRP

There were no statistically significant effects of polyphenol supplementation on CRP immediately post-exercise (SMD = 0.01, 95% CI −0.33, 0.34; *p* = 0.97; participants = 229; studies = 11; I^2^ = 45%), 24 h (SMD = 0.00, 95% CI −0.37, 0.38; *p* = 0.98; participants = 249; studies = 12; I^2^ = 60%), 48 h (SMD = 0.04, 95% CI −0.32, 0.41; *p* = 0.81; participants = 249; studies = 12; I^2^ = 56%) and 72 h post-exercise (SMD = −0.12, 95% CI −0.50, 0.26; *p* = 0.54; participants = 118; studies = 6; I^2^ = 18%). No studies measured CRP at 96 h post-exercise (see Supplementary File 6).

##### IL-6

Polyphenol supplementation had no statistically significant effect on IL-6 immediately (SMD = −0.34, 95% CI −0.76, 0.08; *p* = 0.11; participants = 156; studies = 7; I^2^ = 57%), 24 h (SMD = −0.18, 95% CI −0.64, 0.28; *p* = 0.44; participants = 150; studies = 7; I^2^ = 56%), 48 h (SMD = −0.19, 95% CI −0.68, 0.30; *p* = 0.45; participants = 172; studies = 8; I^2^ = 78%), and 72 h post-exercise (SMD = −0.56, 95% CI −1.25, −0.13; *p* = 0.11; participants = 82; studies = 4; I^2^ = 61%). Only one study measured IL-6 at 96 h post-exercise which showed a moderate but not statistically significant effect (SMD = −0.76, 95% CI −1.80, 0.28; *p* = 0.15; participants = 16; studies = 1) (see Supplementary File 7).

##### PC

Immediately post-exercise (SMD = 0.46, 95% CI −0.67, 1.60; *p* = 0.42; participants = 76; studies = 4; I^2^ = 82%), at 24 h (SMD = 0.39, 95% CI −0.24, 1.01; *p* = 0.22; participants = 96; studies = 5; I^2^ = 66%) and 48 h post-exercise (SMD = 0.33, 95% CI −0.25, 0.92; *p* = 0.27; participants = 96; studies = 5; I^2^ = 63%) polyphenol supplementation caused small increases in PC relative to placebo, although the differences were not statistically significant. There were only two studies (one parallel and one cross-over) that measured PC at 72 h post-exercise; these indicated a moderate-sized effect in favour of polyphenol supplementation (SMD = −0.64, 95% CI −1.14, −0.14; *p* = 0.01; participants = 40; studies = 2; I^2^ = 0%). No studies measured PC at 96 h post-exercise (see Supplementary File 8).

#### 3.5.3. Analyses by Individual Polyphenol-Rich Foods

Individual meta-analyses and subgroup analyses were conducted for tart cherry (*n* = 12), beetroot (*n* = 6), pomegranate (*n* = 3) and cocoa (*n* = 2). When the studies on tart cherry were analysed alone, the magnitude of the SMDs were greater than for all studies combined for MIVC and CMJ, however, statistical significance was attenuated at most time-points. The effect of tart cherry on DOMS was less than for all studies combined except for 72 h post-exercise. For pomegranate, the SMDs for MIVC were smaller than for all studies combined and only statistically significant at 96 h post-exercise. Pomegranate did not cause a statistically significant reduction in DOMS at any of the post-exercise time-points. None of the pomegranate studies measured CMJ height. When individual meta-analyses were conducted for beetroot, the SMDs for DOMS were greater than for all of the studies combined whereas the SMDs for CMJ were slightly attenuated. There were no statistically significant effects of beetroot on MIVC. Two studies analysed the effects of cocoa on recovery of MIVC and DOMS up to 48 h post-exercise. Analysis revealed a statistically significant improvement in MIVC at 48 h post-exercise but no other statistically significant effects (see Supplementary File 9 for all of the individual analyses).

Subgroup analyses revealed no statistically significant differences for MIVC between pomegranate, cherry, beetroot, and cocoa. There were no statistically significant differences between cherry and beetroot for CMJ. No pomegranate studies and only one cocoa study measured CMJ. Both beetroot and cherry reduced DOMS and there were no statistically significant differences between them at any of the post-exercise time-points. Beetroot caused a statistically significant greater reduction in DOMS than pomegranate at 24 (*p* = 0.05) and 72 (*p* = 0.05) h post-exercise. Cherry caused a greater reduction in DOMS than pomegranate, but the difference was only statistically significant at 72 h post-exercise (*p* = 0.05). There were no statistically significant differences for DOMS between cocoa and the three other polyphenol supplements (see Supplementary File 9, Table S4). 

#### 3.5.4. Comparison of Studies Based on Restriction of Dietary Polyphenol Intake

Six studies [12,27,28,39,40,44] directed participants to restrict their intake of dietary polyphenols for the duration of the study, but one reported poor compliance from participants [28]. Comparison of the remaining five studies that restricted polyphenols in the background diet with those studies that did not restrict polyphenols revealed no statistically significant differences for all of the primary outcomes (MIVC, DOMS, and CMJ; see Supplementary File 10 for analyses).

#### 3.5.5. Quality of Evidence

The evidence for MIVC was rated moderate to very low across the post-exercise time-points. The evidence was downgraded for serious risk of bias (all time-points), serious inconsistency (24 h and 96 h), and serious imprecision (immediately, 72 h and 96 h). For DOMS the evidence was rated low to very low. The evidence was downgraded for serious risk of bias (all time-points), serious inconsistency (immediately, 24 h, 48 h and 72 h), and serious imprecision (72 h and 96 h). The evidence was rated low to very low for CMJ. The quality of the evidence was downgraded for serious risk of bias (all time-points), serious inconsistency (24 h and 72 h) and serious imprecision (all time-points) (See Supplementary File 3).

## 4. Discussion

In this meta-analysis, polyphenol-rich foods accelerated the recovery of muscle function and reduced muscle soreness post EIMD, however, the certainty of the evidence was moderate to very low. The SMDs for the effects of polyphenol supplementation were small to moderate for our primary outcomes. When the SMDs were converted into percentages, benefits ranged from 5.7% to 13.0% for MIVC, 9.3% to 29.3% for muscle soreness, and 5.5% to 13.1% for CMJ.

A reduction of 14% [49] has been proposed as the minimal important difference (MID) [50] in muscle soreness. In this study, the difference between polyphenol supplementation and placebo was comparable to the lower threshold of the MID at most post-exercise time-points but much greater at 72 h. Recovery of muscle strength and explosive lower body power after EIMD was commonly assessed by measuring MIVC and CMJ, respectively. To our knowledge, there are no consensus guidelines as to the MIDs for these measures. We found that polyphenol supplementation accelerated recovery of CMJ by between 5.5–13.1% and MIVC by 5.7–13.0% in comparison to a placebo. For sports that require explosive lower body power and maximal strength where limited recovery time is available between events, differences of this magnitude could translate into meaningful performance effects, however, there is a need to determine the MID for MIVC and CMJ to aid the interpretation of meta-analyses.

The studies included in this meta-analysis investigated the effect of eight different types of polyphenol-rich plant-based foods. Different plants vary in their profile of polyphenols [51] and other bioactive compounds (e.g., beetroot is rich in nitrate [15] and betalains [16], which could determine their ability to accelerate recovery from EIMD. Thus, we explored the efficacy of individual polyphenol-rich foods. Our meta-analysis of 12 studies on tart cherry found evidence of enhanced recovery of MIVC, CMJ height and muscle soreness at some post-exercise time-points. Analysis of five studies on beetroot found no evidence of a benefit for MIVC, but faster recovery of CMJ and muscle soreness. Whereas, for pomegranate, analysis of three studies found limited evidence of faster recovery of MIVC, but no effect on muscle soreness. For cocoa there was no evidence of a benefit for muscle soreness but there was a faster recovery of MIVC at 48 h post-exercise. There were insufficient studies to explore the individual effects of the other polyphenol-rich supplements. Subgroup analyses failed to resolve any statistically significant differences between food type for MIVC or CMJ. The reduction in DOMS from beetroot juice was statistically greater than pomegranate juice at 24 h and 72 h, whereas for tart cherry DOMS was only significantly lower than pomegranate at 72 h. Interpretation of these subgroup analyses needs to be cautious because of the small number of studies included, especially for pomegranate and cocoa.

The ability of polyphenol supplements to accelerate recovery would be expected to be greater in participants that restricted their intake of dietary polyphenols. However, subgroup analyses failed to detect any statistically significant differences in markers of recovery when we compared studies that instructed participants to restrict their diet with those that had no restrictions. A number of limitations in the literature may explain these findings. First, there was substantial heterogeneity in study design, second, there were a limited number of studies [12,27,39,40,44] that restricted polyphenols, third, only two [27,40] of the five studies included a washout period that excluded polyphenol consumption before the intervention, and finally, no studies reported the habitual intakes of dietary polyphenols in their participants, which could have influenced the efficacy of the interventions. Thus, there is a need for future studies in this area to better characterise the polyphenol intakes of their participants.

### 4.1. Mechanisms

Polyphenol supplementation failed to reduce CK at any of the post-exercise time-points. Serum CK levels are commonly used to assess muscle damage post-exercise, however, the increase in serum CK does not directly correlate with the degree of muscle damage and the loss of force production [18,52]. Also there is substantial inter-individual variability in CK response after muscle damage [53]. Therefore, CK may have limited ability to detect differences in the extent of muscle damage in response to a dietary/recovery intervention [11,28] which probably explains the lack of treatment effect observed in this meta-analysis.

It has been suggested that polyphenols may accelerate recovery through enhancing antioxidant status and supressing inflammation [1]. A number of studies in this review measured total antioxidant status (TAS) in the blood [12,34,36]. We did not conduct a meta-analysis on TAS because it is no longer recommended as a valid method of measuring antioxidant status in vivo [54]. Some studies in this review measured blood-borne markers of oxidative stress such as F2-isoprostanes, lipid hydroperoxides, and PC. We did not pool all of the oxidative stress markers into a single meta-analysis because they are not directly comparable [55,56]. We only analysed the data for PC because it was the only oxidative stress marker measured in multiple studies. There was no effect, except at the 72 h time-point, however, the analysis at 72 h only included two studies [12,13].

The most commonly analysed markers of inflammation in the studies included in this review were CRP and IL-6. We found no effect of polyphenol supplementation on CRP or IL-6 at any post-exercise time-point. This may be because serum markers do not accurately mirror intramuscular processes [57]. To elucidate the mechanisms through which polyphenols accelerate recovery, future studies should complement serum markers with intramuscular measures of antioxidative status, oxidative stress, and inflammation. 

### 4.2. Comparison with Other Reviews

Our study expands the findings from a previous meta-analysis of fruit supplements and recovery from EIMD by Doma et al. [17]. In agreement with Doma et al. [17] we found that polyphenol supplementation caused a faster recovery of MIVC at 24 h and 48 h post-exercise but we found smaller SMDs. Notably, Doma et al. (2020) investigated effects only at 24 h and 48 h post-exercise despite the recovery process often extending beyond this [2]. Whereas in our review we observed beneficial effects of polyphenol supplementation for up to 96 h post-exercise. While we found beneficial effects on DOMS at 24, 48 and 72 h post-exercise, Doma et al. [17] only reported positive effects at 24 h and did not examine responses at the 72 h timepoint. In a number of studies included in our meta-analysis DOMS was still elevated at 72 h.

Doma et al. [17] found a reduction in markers of muscle damage, inflammation, and oxidative stress at 24 and 48 h post-exercise whereas, we found no effect at those time-points but observed moderate reductions at 72 h for PC. Possible explanations for the disagreement between our findings and those of Doma et al. [17] include differences in studies selected for the meta-analysis, focusing on polyphenol-rich food, juices and concentrates from fruit and vegetables whilst excluding extracts, and our decision not to pool markers of inflammation and oxidative stress. When studies measured more than one blood marker of muscle damage, inflammation, or oxidative stress, Doma et al. [17] combined these into a single estimate of effect in their meta-analysis. This is problematic because some of the biochemical markers they combined are not directly comparable. For example, for oxidative stress they combined PC, TBARS, uric acid and superoxide dismutase and these are markers of different processes that may not correlate with each other.

Hill et al. [58] conducted a systematic review and meta-analysis on the effect of tart cherry (juices, concentrates, and extracts) and recovery from EIMD. They found moderate beneficial effects on muscular power and recovery of muscular strength, and small beneficial effects on DOMS. However, Hill et al. [58] combined all post-exercise time points into a single summary estimate of effect for each outcome marker. This makes direct comparison with our meta-analysis impossible because we investigated the effects at each post-exercise time point separately in order to capture the time course of recovery and to avoid unit of analysis errors [20,29]. We found weaker evidence than Hill et al. [58] that tart cherry supplementation enhanced recovery after EIMD with statistically significant effects on MIVC, DOMS and CMJ only at a limited number of post-exercise time points (see Supplementary File 9).

### 4.3. Limitations 

The results of this systematic review and meta-analysis should be interpreted cautiously considering several limitations in the included studies. Ten of the 25 studies included in this meta-analysis were categorised as high risk of bias but when these were removed in a sensitivity analysis the beneficial effects of polyphenol supplementation were attenuated. There was moderate to substantial heterogeneity for several outcomes across various time points. Many of our subgroup analyses were conducted on a small number of studies which limited the statistical power to detect differences.

The studies included in the meta-analyses varied in design. Of the 25 studies, only five [38,39,43,45,47] recruited well-trained athletes (11.3% of the total participants). Moreover only 19.4% of the participants included in this review were female. Male and female participants were combined in five studies [11,35,36,37,44]. It has been reported that oestrogen may protect muscle from injury by reducing inflammation [59], however, these studies failed to report whether they controlled for stages of the menstrual cycle. Studies varied in the duration outcomes markers were measured post EIMD ranging from immediately to 96 h after exercise. The studies of shorter duration (i.e., up to 48 h) may not have accurately captured the time-course of response for some markers of recovery [2].

Five studies included in this review did not report the polyphenolic composition of the products they administered. Moreover, when polyphenol composition was reported, authors mainly relied on published data rather than analysing the batch of polyphenol supplement they used. Because of limited and possibly unreliable data on the polyphenol content of supplements used we could not explore a dose response relationship in this meta-analysis.

We downgraded the quality of the evidence for all primary outcomes across all time-points to moderate- to very-low. The main reasons for downgrading were serious risk of bias, small overall sample sizes, moderate to substantial heterogeneity and imprecision in summary effect estimates.

### 4.4. Future Research 

To clarify the effects of polyphenols on recovery from EIMD there is a need for future studies to accurately characterise the composition of the products used, identify the optimal dose, measure the appearance of polyphenolic metabolites in urine and blood, and utilise tissue markers of muscle damage, inflammation, and antioxidant status. It remains unclear what the optimal duration of polyphenol supplementation is and whether ingestion should start before or after muscle damage has incurred. A possible concern of long-term polyphenol supplementation is that it may blunt important stress signals required for the adaptive response to exercise [3] however, this requires confirmation. This review highlighted that females were underrepresented in the literature and no studies had directly compared the differences in the effects of polyphenol supplementation on recovery between sexes, thus future research opportunities exist to address this gap. The majority of participants included in the studies were recreationally-trained athletes. It is important to confirm the effects of polyphenol supplementation in well-trained and elite athletes who are more likely to experience limited recovery times between competitions. All of the studies included in this review were rated as ‘some concerns’ or ‘high’ risk of bias. Thus, there is a need for higher quality randomised controlled trials investigating polyphenols on recovery from EIMD.

## 5. Conclusions

Our systematic review and meta-analysis found moderate to very low certainty evidence that polyphenol-rich foods, juices, and concentrates accelerate recovery of muscle function (up to 13%) and reduce muscle soreness (up to 29%) after EIMD. This magnitude of enhanced recovery could benefit athletes in scenarios where there is limited time between competitive events and impaired recovery could negatively impact performance. Polyphenol supplementation may be useful in situations where EIMD impairs muscle function for extended periods of time such as after very heavy training loads, or at the initiation of a training programme. The mechanisms through which polyphenol supplementation enhances recovery is uncertain, but inhibition of inflammation and enhanced antioxidant capacity may be important. That said, in this review, little evidence of a reduction in either inflammation or oxidative stress was evident. This could reflect limitations in the blood-based methods used by the studies included in this review. Future studies should adopt robust methods of determining inflammation and redox status within muscle tissue to unravel the mechanisms through which polyphenols enhance recovery from EIMD.

In conclusion, our review suggests that consumption of polyphenol-rich foods, juices, and concentrates provides practitioners and athletes with a low risk, food-first approach for enhancing recovery in scenarios where optimising rapid recovery is key. Further investigation is required to determine the optimal duration and dosage of polyphenol supplementation and explore whether enhanced recovery comes at the expense of impaired adaptation. There is also a need for more studies to investigate the effect in well-trained and elite athletes.

## Figures and Tables

**Table 1:** Characteristics of the studies included in the systematic review and meta-analysis.

Authors	Participant Characteristics at Baseline	Study Design	Exercise Intervention	Polyphenol Supplement	Dosage and Duration	Outcome Variables and Time of Measurement (h)
Abbot et al. 2019 [38)]	Professional male soccer players Age 19 ± 1 years Height 1.8 ± 0.6 m Mass 77.3 ± 6.4 kg *n* = 10	Crossover	90 min soccer match	Tart cherry juice Polyphenol content not stated	2 × 30 mL concentrate for 3 days (morning of match until 36 h post-exercise)	DOMS (12, 36, 60) CMJ (12, 36, 60)
Bell et al. 2015 [39]	Male healthy trained cyclists Age 30 ± 8 years Height 181.1 ± 6.7 cm Mass 76.5 ± 9.2 kg $\dot{V}$O_2peak_ 61.6 ± 10.4 mL·kg^−1^·min^−1^ *n* = 16	Parallel	109 min stochastic cycling	Montmorency tart cherry juice Per 1 mL 9.2 mg of anthocyanins (HPLC) Atlas Biosciences, Tuscon, Arizona, USA	2 × 30 mL concentrate for 8 days (4 days pre-exercise, on the day of, and 3 days post-exercise)	MIVC (PE, 24, 48, 72) DOMS (PE, 24, 48, 72) CK (PE, 24, 48, 72) CRP (PE, 24, 48, 72) IL-6 (PE, 24, 48, 72)
Bell et al. 2016 [40]	Male semi-professional soccer players Age 25 ± 4 years Height 180.8 ± 7.4 cm Mass 81.9 ± 6.6 kg $\dot{V}$O_2peak_ 54.9 mL·kg^−1^·min^−1^ *n* = 16	Parallel	Loughborough intermittent shuttle test	Montmorency tart cherry juice Per 1000 mL 73.5 mg cyanidin-3-glucoside (HPLC) 178.8 mg of GAE (total phenols) 0.58 trolox equivalent (TEAC) Based on previous work from own laboratory (61)	2 × 30 mL concentrate for 8 days (4 days pre-exercise, on the day of, and 3 days post-exercise)	MIVC (PE, 24, 48, 72) DOMS PE, 24, 48, 72) CMJ (PE, 24, 48, 72) CK (PE, 24, 48, 72) CRP (PE, 24, 48, 72) IL-6 (PE, 24, 48, 72)
Bowtell et al. 2011 [34]	Male well-trained participants Age 27.8 ± 1.6 years Height 1.76 ± 0.03 m Mass 81.3 ± 4.3 kg *n* = 10	Crossover	10 × 10 single leg extension	Montmorency tart cherry juice 275 mmol·L^−1^ trolox equivalents (ORAC) (Brunswick Laboratories, Southborough, USA) Per 1 mL 9.117 mg of anthocyanins: malvidin (4.696 mg) and cyanidin (3.346 mg) (HPLC) (Atlas, Biosciences, Tucson, Arizona, USA)	2 × 30 mL concentrate for 10 days (7 days pre-exercise, on the day of, and 2 days post-exercise)	MIVC (PE, 24, 48) CK (24, 48) CRP (PE, 24, 48) PC (PE, 24, 48)
Brown, Stevenson and Howatson (2019) [41]	Female physically active females Age 19 ± 1 years Height 167 ± 6 cm Mass 61.4 ± 5.7 kg BMI 22.1 ± 1.9 kg m^−2^ *n* = 20	Parallel	15 × 30 m repeated sprints with 30 s rest	Montmorency tart cherry juice Per 1000 mL 73.5 mg cyanidin-3-glucoside (HPLC) 178.8 mg of GAE (total phenols) 0.58 trolox equivalent (TEAC) Based on previous work from own laboratory (61).	2 × 30 mL concentrate for 8 days (4 days pre-exercise, on the day of, and 3 days post-exercise)	MIVC (PE, 24, 48, 72) DOMS (PE, 24, 48, 72) CMJ (PE, 24, 48, 72) CK (PE, 24, 48, 72) CRP, (PE, 24, 48, 72)
Clifford et al. 2016a [13]	Male collegiate team sport players Age 22 ± 2.5 years Height 1.80 ± 0.70 m Mass 75.1 ± 10.9 kg *n* = 20	Parallel	20 × 30 m sprints on day 1 and day 4 (72 h apart)	Beetroot juice Per 1000 mL 1606.9 ± 151 mg GAE (total phenols) 11.4 ± 0.2 mmol trolox equivalents (TEAC) (62).	2 × 250 mL for 3 days (day of and 2 days post-exercise)	MIVC (PE, 24, 48, 72, 96) CMJ (PE, 24, 48, 72, 96) CK (PE, 24, 48, 72, 96) CRP (PE, 24, 48, 72, 96) PC (PE, 24, 48, 72, 96)
Clifford et al. 2016b [27]	Male recreational active participants Age 21.3 ± 4 years Height 178 ± 0.76 m Mass 75.6 ± 8.3 kg *n* = 30	Parallel	5 × 20 drop jumps	Beetroot juice (high 250 mL vs. low dose 125 mL) Per 250 mL 401.72 ± 37.72 mg GAE (total phenols) 2.85 ± 0.05 mmol trolox equivalents (DPPH)	3 × servings on day of exercise and 2 × servings for two days post-exercise	MIVC, (PE, 24, 48, 72) CMJ (PE, 24 48, 72) CK (PE, 24, 48, 72) IL-6 (PE, 24, 48, 72)
Clifford et al. 2017a [35]	Recreational runners Age 40.5 ± 11 years Height 1.71 ± 0.08 cm Mass 70.3 ± 10.85 kg *n* = 34 (m = 21; f = 13)	Parallel	Druridge Bay Marathon (Northumbria, UK)	Beetroot juice (250 mL) Per 250 mL ~400 mg GAE (total phenols) ~3 mmol trolox equivalents (DPPH) (62).	3 × servings on day of exercise and 2 × servings for two days post-exercise	MIVC (PE, 24, 48) DOMS (PE, 24, 48) CMJ (PE, 24, 48) CK (PE, 24, 48) CRP (PE, 24, 48) IL-6 (PE, 24, 48)
Clifford et al. 2017b [42]	Male healthy untrained participants Age 21.7 ± 2.3 years Height 178.0 ± 6.46 cm Mass 75.1 ± 10.13 kg *n* = 30	Parallel	5 × 20 drop jumps	Beetroot juice (250 mL) Per 250 mL ~400 mg GAE (total phenols) (62).	3 × servings on day of exercise and 2 × servings for two days post-exercise	MIVC (PE, 24, 48, 72) CMJ (PE, 24, 48, 72) CK, (PE, 24, 48, 72) CRP (PE, 24, 48, 72)
Connoll, McHugh and Padilla Zakour 2006 (9)	Male Age 22 ± 4 y ears Height 1.78 ± 0.86 m Mass 90 ± 18 kg *n* = 16	Crossover	2 × 20 eccentric elbow contractions	Tart cherry juice and apple juice blend Per 12 fl oz 600 mg of GAE (total phenols) 40 mg of cyanidin-3-glucoside equivalents (pH differential)	2 × 12 fl oz bottles for 8 days (3 days pre-exercise, on the day of, and 4 days post-exercise)	MIVC (24, 48, 72, 96) DOMS, (24, 48, 72, 96)
Daab et al. 2020 [43]	Male semi-professional soccer players Age 22.1 ± 0.56 years Height 178 ± 1.19 cm Mass 75.8 ± 5.58 kg *n* = 13	Crossover	Loughborough intermittent shuttle test	Beetroot juice Polyphenol content not stated	2 × 150 mL per day for 7 days (3 days pre-exercise, on the day of, and 3 days post-exercise)	MIVC (PE, 24, 48, 72) DOMS (PE, 24, 48, 72) CMJ (PE, 24, 48, 72) CK (PE, 24, 48, 72) CRP (PE, 24, 48, 72)
Howatson et al. 2010 [36]	Marathon runners Age 37.5 ± 9 years Height 1.76 ± 0.07 m Mass 73.3 ± 9.85 kg *n* = 20 (m = 13; f = 7)	Parallel	London Marathon (UK)	Tart cherry juice and apple juice blend 55 mmol·L^−1^ trolox equivalents (ORAC) Per 12 fl oz 600 mg of GAE (total phenols) 40 mg of cyanidin-3-glucoside equivalents (pH differential) (9).	2 × 8 fl oz bottles for 8 days (5 days pre-exercise, on the day of, and 2 days post-exercise)	MIVC (PE, 24, 48) DOMS (PE, 24,48) CK (PE, 24, 48) CRP (PE, 24, 48) IL-6 (PE, 24 48) PC (PE, 24, 48)
Hutchison et al. 2016 [44]	Healthy participants Age 20.2 ± 0.6 years Height 165.75 ± 3 cm Mass 64.2 ± 5 kg *n* = 16 (m = 3; f = 13)	Parallel	3 × 10 eccentric squats	Blackcurrant nectar 7340 μmol trolox equivalents (TEAC) Per 16 fl oz 193.25 mg malvidin glucosides (pH differential) 175.69 mg cyanidin glucosides (pH differential)	16 fl oz bottle twice per day for 8 days (4 days pre-exercise, on the day of, and 3 days post-exercise)	DOMS (PE, 24, 48, 96) CK (PE, 24, 48, 96) IL-6 (PE, 24, 48, 96)
Kuehl et al. 2010 [37]	Healthy runners Age 35.8 ± 9.6 years *n* = 54 (m = 36; f = 18)	Parallel	Oregon Hood to Coast Relay Race (USA)	Montmorency tart cherry juice and apple juice blend Per 12 fl oz 600 mg of GAE (total phenols) 40 mg of cyanidin-3-glucoside equivalents (pH differential) (9).	2 × 355 mL tart cherry juice for 8 days (7 days pre-exercise, on the day of the trial)	DOMS (PE)
Kupsaravic, McShane and Clifford. 2019 [45]	Elite male rugby union players Age 28 ± 4 years Height 1.88 ± 0.64 m Mass 106.8 ± 7.6 kg *n* = 10	Crossover	Rugby Union match	Montmorency tart cherry juice Polyphenol content not stated	2 × 30 mL concentrate for 5 days (2 days pre-exercise, on the day of, 2 days post-exercise)	DOMS (24, 48, 72)
Lamb et al. 2019 [28]	Male non-resistance trained Age 24 IQR 22,33 years BMI 25.6 ± 4 kg m^−2^ *n* = 36	Parallel	5 × 10 eccentric elbow contractions non dominant arm	Montmorency tart cherry juice and Pomegranate Wonderful juice Tart cherry juice per 30 mL 294.7 ± 14.9 mg GAE (total phenols) 7.7 ± 0.3 mg anthocyanins (pH differential) Pomegranate Wonderful per 250 mL 878.9 ± 92.7 mg GAE (total phenols) 49.4 ± 2.0 mg total anthocyanins (pH differential)	2 × 30 mL of concentrate for 9 days (cherry) and 2 × 250 mL for 9 days (pomegranate) (4 days pre-exercise, on the day of, 4 days post-exercise)	MIVC (PE, 24, 48, 72, 96) DOMS (PE, 24, 48, 72, 96) CK (PE, 24, 48, 72, 96)
Lima et al. 2019 [46]	Healthy male physical education students Age 22.3 ± 2.6 years Height 176.6 ± 6.4 cm Mass 77.1 ± 10.5 kg *n* = 30	Parallel	30 min downhill run at 70% $\dot{V}$O_2max_	Anthocyanin-rich antioxidant juice that consisted of a mixture of clarified apple juice with plum, blueberry, maquiberry, raspberry and cranberry 67,680 μmol·mL^−1^ of trolox equivalents (ORAC) Per 240 mL 58 mg of anthocyanins	2 × 240 mL for 9 days (4 days pre-exercise, on the day of, 4 days post-exercise)	MIVC (PE, 24, 48, 72. 96) DOMS (24, 48, 72, 96) CK (48, 96)
Lynn et al. 2018 [11]	Recreational runners Age 30.9 ± 10.53 years Height 1.74 ± 0.08 m Mass 71.4 ± 10.5 kg BMI 23.5 ± 2.45 kg m^−2^ *n* = 21 (m = 16; f = 5)	Parallel	Sheffield Half Marathon (UK)	Bilberry juice Per 200 mL 744.14 ± 81.75 mg of GAE (total phenols) 80.04 ± 3.51 mg of total anthocyanins (pH differential)	2 × 200 mL for 8 days (5 days pre-exercise, on the day of, 2 days post-exercise)	DOMS (PE, 24, 48) CK (PE, 24, 48) CRP (PE, 24, 48)
Machin et al. 2014 [25]	Male non-resistance trained Age 22.3 ± 4.1 years Height 174.9 ± 6.2 cm Mass 73.8 ± 11.5 kg *n* = 45	Parallel	20 min of downhill running and 40 repetitions of bilateral eccentric elbow contractions	Pomegranate Wonderful juice (high 2 × 30 mL vs. low 1 × 30 mL) Per 30 mL 650 mg of GAE consisting of 95.5% ellagitannins, 3.5% ellagic acid, and 1% anthocyanins	1 or 2 × servings for 8 days (3 days pre-exercise, on the day of, 4 days post-exercise)	MIVC (PE, 24, 48, 72, 96) DOMS (PE, 24, 48, 72, 96)
McLeay et al. 2012 [12]	Healthy recreational females Age 22 ± 1 years Height 167 ± 5 cm Mass 62 ± 8 kg *n* = 14	Crossover	3 × 100 eccentric knee extensions	Smoothie with New Zealand blueberries (200 g), banana (~50 g) and apple juice (200 mL) 5417 μmol trolox equivalents (ORAC) Per 100 mL 168 mg of GAE (total phenols) 96.6 mg of anthocyanins 26 mg of phenolic acid 10.2 mg of flavonoids	3 × servings on the day of exercise and 1 × serving for 2 days post-exercise	MIVC (12, 36, 60) DOMS (12, 36, 60) CK (12, 36, 60) IL-6 (12, 36, 60) PC (12, 36, 60)
Morehen et al. 2020 [47]	Male Professional Rugby players Age 18 ± 1 years Height 182 ± 0.04 cm Mass 92.2 ± 8.6 kg *n* = 11	Crossover	Rugby Union match	Montmorency tart cherry juice Per 30 mL 320 mg of anthocyanins	2 × 30 mL concentrate per day for 7 days, (4 days pre-match, on the day of, 2 days post-match)	DOMS (24, 48) CMJ (48) IL-6 (PE, 48)
Morgan et al. 2018 [14]	Healthy recreational active males Age 22.8 ± 3.3 years Height 1.84 ± 0.59 cm Mass 85.3 ± 12 kg *n* = 10	Crossover	Single leg extension 10 × 10 repetitions at 80% 1RM	Ecuadorian cacao juice (ZumaCacao^®^) Per 330 mL serving 154 mg of polyphenols 8 mg epicatechin 43 mg catechins 23 mg flavanols 12 mg proanthocyanidins HPLC -Atlas, Bioscience, Inc, Tucson, Arizona, USA	330 mL per day for 10 days (7 days pre-exercise, on the day of, 2 days post-exercise)	MIVC (PE, 24, 48) DOMS (PE, 24, 48) CMJ (PE, 24, 48) CK (PE, 24, 48) CRP (PE, 24, 48) PC (PE, 24, 48)
Peschek et al. 2014 [26]	Male well trained runners and triathletes Age 24.6 ± 5.6 years Height 182.1 ± 6.3 cm Mass 73.4 ± 7 kg Body fat percentage 13.7 ± 5.1% *n* = 8	Crossover	30 min downhill run at 70% $\dot{V}$O_2max_	Unsweetened Cocoa Via consisted of cocoa powder, salt, and soy lecithin Per 240 mL 350 mg flavanols	1 g·kg^−1^ of body weight of cocoa milk at 1 h and at 2 h post-exercise	MIVC (24, 48) DOMS (24, 48) CK (24, 48)
Quinlan and Hill 2020 [48]	Team sport recreational athletes Age 26.5 ± 4.5 years Height 175.3 ± 9.75 cm Mass 70.2 ± 12.85 kg Predicted $\dot{V}$O_2peak_ 44.4 ± 8.1 mL·kg^−1^·min^−1^ *n* = 20 (m = 8; f = 12)	Parallel	Loughborough intermittent shuttle test followed by 12 × 20 m sprints	Montmorency tart cherry juice Polyphenol content not stated	2 × 30 mL concentrate for 8 days (5 day pre-exercise, on the day of, 2 days post-exercise)	MIVC (PE, 24, 48) DOMS (PE, 24, 48) CMJ (PE, 24, 48) CK (PE, 24, 48) CRP (PE, 24, 48)
Trombold et al. 2011 [10]	Male recreational active Age 21.9 ± 2.4 years Height 179.1 ± 8.4 cm Mass 80.2 ± 7.5 kg *n* = 17	Crossover	3 × 20 eccentric elbow contractions, 6 × 10 eccentric knee contractions	Pomegranate Wonderful juice Per 1000 mL 1979 mg of tannins 384 mg of anthocyanins 121 mg of ellagic acid derivatives (Content obtained from the manufacturer)	2 × 250 mL for 15 days (8 days pre-exercise, on the day of, 6 days post-exercise)	MIVC (PE, 24, 48, 72, 96) DOMS, (PE, 24, 48, 72, 96)

BMI, body mass index; CK, creatine kinase; CMJ, countermovement jump; CRP, c-reactive protein; DOMS, delayed onset muscle soreness; DPPH, 2,2-diphenyl-1-picrylhydrazyl; GAE, gallic acid equivalents; h, hours; HPLC, high performance liquid chromatography; IQR, interquartile range; IL-6, interleukin-6; MIVC, maximal isometric voluntary contraction; ORAC, oxygen radical absorbance capacity; PC, protein carbonyls; PE, post-exercise; RM, repetition maximum; TEAC, trolox equivalent antioxidant capacity; $\dot{V}$O_2max_, maximal oxygen uptake; $\dot{V}$O_2peak_, peak oxygen uptake.

## Data Availability

Data are contained within the article or available from the included studies that have been cited throughout.

## Supplementary Materials

The following are available online at https://www.mdpi.com/article/10.3390/nu13092988/s1, Supplementary File 1: Database search strategy; Supplementary File 2: Funnel Plots; Supplementary File 3: Quality assessment; Supplementary File 4: Sensitivity analysis; Supplementary File 5: creatine kinase forest plot; Supplementary File 6: C-reactive protein forest plot; Supplementary File 7: Interleukin-6 forest plot; Supplementary File 8: Protein carbonyls forest plot; Supplementary File 9: Analysis by individual polyphenol-rich foods; Supplementary File 10: Polyphenol restriction forest plot.
