# Supplementary material for: Effect of Polyphenol-Rich Foods, Juices, and Concentrates on Recovery from Exercise Induced Muscle Damage: A Systematic Review and Meta-Analysis
Source: Nutrients. 2021 Aug 27;13(9):2988. doi: 10.3390/nu13092988 (PMC8465563; doi:10.3390/nu13092988)
Supplement: Supplementary file 1 [file nutrients-13-02988-s001.zip › Supplementary Fie 1 Database search strategies.pdf]

Supplementary File 1 Database search strategy

Sport Discus search terms

TI (pomegranate* OR blueberr* OR bilberr* OR blackcurrant* OR cocoa* OR "green tea"* OR "black tea"* OR quercetin* OR grape* OR beetroot* OR "red wine"* OR soy* OR anthocyanin* OR flavones* OR isoflavones* OR flavanols* OR resveratrol* OR picied* OR benzoic* OR cinnamic* OR cherry*) OR AB (pomegranate* OR blueberr* OR bilberr* OR blackcurrant* OR cocoa* OR "green tea"* OR "black tea"* OR quercetin* OR grape* OR beetroot* OR "red wine"* OR soy* OR anthocyanin* OR flavones* OR isoflavones* OR flavanols* OR reservatrol* OR picied* OR benzoic* OR cinnamic* OR cherry*)

TI (cycling OR running OR soccer OR rugby OR BOXING or BASKETBALL OR gymnastics OR GOLF OR WRESTLING OR "MARTIAL ART*" OR ATHLETICS OR TENNIS OR SQUASH OR BADMINTON OR BASEBALL OR SKIING OR SKATING OR DIVING OR SWIMMING OR VOLLEYBALL OR HOCKEY OR CRICKET OR WALKING) OR AB (cycling OR running OR soccer OR rugby OR BOXING or BASKETBALL OR gymnastics OR GOLF OR WRESTLING OR "MARTIAL ART*" OR ATHLETICS OR TENNIS OR SQUASH OR BADMINTON OR BASEBALL OR SKIING OR SKATING OR DIVING OR SWIMMING OR VOLLEYBALL OR HOCKEY OR CRICKET OR WALKING)

DE "PHYSICAL training & conditioning" OR DE "ALTITUDE training" OR DE "ANAEROBIC training" OR DE "ARCHERY training" OR DE "BADMINTON training" OR DE "BASE training (Exercise)" OR DE "BASEBALL training" OR DE "BASKETBALL training" OR DE "BICYCLE racing training" OR DE "BODYBUILDING training" OR DE "BOWLING training" OR DE "BULLFIGHT training & conditioning" OR DE "CANOEING training" OR DE "CAVING training & conditioning" OR DE "COMPOUND exercises" OR DE "CONTINUOUS training (Exercise)" OR DE "CONTRAST training (Physical training & conditioning)" OR DE "COXSWAINING training" OR DE "CRICKET training & conditioning" OR DE "CROSS-training (Sports)" OR DE "CYCLING training" OR DE "DANCE training & conditioning" OR DE "DEEP diving training & conditioning" OR DE "DIVING training" OR DE "DOGSLEDDING training & conditioning" OR DE "ENDURANCE sports training" OR DE "FENCING training" OR DE "FIELD hockey training & conditioning" OR DE "FOOTBALL training" OR DE "FUNCTIONAL training" OR DE "GLIDING & soaring training & conditioning" OR DE "GOLF training" OR DE "GYMNASTICS training" OR DE "HANDBALL training & conditioning" OR DE "HIKING training & conditioning" OR DE "HOCKEY training" OR DE "HUNTING training & conditioning" OR DE "INTERVAL training" OR DE "ISOLATION exercises" OR DE "KAYAKING training" OR DE "KNIFE fighting -- Training" OR DE "LACROSSE training & conditioning" OR DE "LONG slow distance training" OR DE "MARTIAL arts training" OR DE "MOTORSPORTS training & conditioning" OR DE "MOUNTAINEERING training" OR DE "ORIENTEERING training" OR DE "OVERTRAINING" OR DE "PACE training" OR DE "PARACHUTING training & conditioning" OR DE "PERIODIZATION training" OR DE "PERSONAL training" OR DE "POLO training & conditioning" OR DE "PRACTICE (Sports)" OR DE "PRESEASON (Sports)" OR DE "RACQUETBALL training" OR DE "RECOVERY training" OR DE "RELAY racing -- Training" OR DE "REPETITION training" OR DE "RESISTANCE training (Physical training & conditioning)" OR DE "ROCK climbing training" OR DE "RODEO training & conditioning" OR DE "ROLLER skating training & conditioning" OR DE "ROWING training" OR DE "RUGBY football training" OR DE "RUNNING training" OR DE "SHOT putting training" OR DE "SKATING training" OR DE "SKI training" OR DE "SKYDIVING training & conditioning" OR DE "SOCCER training" OR DE "SOFTBALL training" OR DE "SPEED endurance training" OR DE "SQUASH training" OR DE "STRENGTH training" OR DE "SURFING training" OR DE "SWIMMING training" OR DE "TABLE tennis training & conditioning" OR DE "TEAM handball training" OR DE "TENNIS training" OR DE "TRACK & field -- Training" OR DE "TRAINING of acrobats" OR DE "TRAINING of boxers (Sports)" OR DE "TRIATHLON training" OR DE "TUG of war (Game) -- Training" OR DE "VAULTING (Horsemanship) -- Training" OR DE "VOLLEYBALL training" OR DE "WATER polo training" OR DE "WEIGHT training" OR

DE "WHEELCHAIR sports training" OR DE "WINTER sports training & conditioning" OR DE "WRESTLING training" OR DE "YOGA training & conditioning"

Cochrane Central Register search terms

pomegranate* or blueberr* or bilberr* or blackurrant* or cherr* or cocoa* or "green tea" or "black tea" or quercetin* or grape* or "red wine" or beetroot* or polyphenol* or flavonoid* or soy* or anthocyanin* or flavones* or isoflavones* or flavanols* or reservatrol* or Picied* or benzoic* or Cinnamic*:ti,ab

exercis* or "mechanical exercise*" or "metabolic exercise*" or "resistance training" or "eccentric training" or "circuit training" or "weight training" or "plyometric training" or weightlifting or cycling or netball or tennis or soccer or rugby or boxing or basketball or gymnastics or golf or wrestling or "martial art*" or athletics or "table tennis" or squash or badminton or baseball or skiing or skating or diving or swimming or volleyball or hockey or cricket or walking or sport*:ti,ab

Pubmed search terms

((((((pomegranate*[Title/Abstract] OR blueberr*[Title/Abstract] OR bilberr*[Title/Abstract] OR BLACKCURRANT*[Title/Abstract] OR COCOA*[Title/Abstract] OR "GREEN TEA*"[Title/Abstract] OR "BLACK TEA*"[Title/Abstract] OR QUERCETIN*[Title/Abstract] OR GRAPE*[Title/Abstract] OR "RED WINE*"[Title/Abstract] OR BEETROOT*[Title/Abstract] OR SOY*[Title/Abstract] OR CHERR*[Title/Abstract] OR POLYPHENOL*[Title/Abstract] OR ANTHOCYANIN*[Title/Abstract] OR FLAVONES*[Title/Abstract] OR ISOFLAVONES*[Title/Abstract] OR FLAVANOLS*[Title/Abstract] OR RESVERATROL*[Title/Abstract] OR PICIED*[Title/Abstract] OR BENZOIC*[Title/Abstract] OR CINNAMIC*[Title/Abstract]))) OR (((((((((((((((blackcurrant[MeSH Terms]) OR blueberry[MeSH Terms]) OR pomegranate[MeSH Terms]) OR green tea[MeSH Terms]) OR black tea[MeSH Terms]) OR quercetin[MeSH Terms]) OR grape[MeSH Terms]) OR red wine[MeSH Terms]) OR soy[MeSH Terms]) OR anthocyanin[MeSH Terms]) OR flavones[MeSH Terms]) OR isoflavones[MeSH Terms]) OR flavonoid[MeSH Terms]) OR benzoic[MeSH Terms]) OR cinnamic[MeSH Terms]) OR cherry[MeSH Terms]))) AND ((((exercis*[Title/Abstract] OR "RESISTANCE EXERCISE"[Title/Abstract] OR "ECCENTRIC TRAINING"[Title/Abstract] OR WEIGHTLIFTING[Title/Abstract] OR "CIRCUIT TRAINING"[Title/Abstract] OR "WEIGHT TRAINING"[Title/Abstract] OR "PLYOMETRIC TRAINING"[Title/Abstract] OR CYCLING[Title/Abstract] OR RUNNING[Title/Abstract] OR SOCCER[Title/Abstract] OR RUGBY[Title/Abstract] OR BOXING[Title/Abstract] OR BASKETBALL[Title/Abstract] OR GYMNASTICS[Title/Abstract] OR GOLF[Title/Abstract] OR WRESTLING[Title/Abstract] OR "MARTIAL ART*"[Title/Abstract] OR ATHLETICS[Title/Abstract] OR TENNIS[Title/Abstract] OR SQUASH[Title/Abstract] OR BADMINTON[Title/Abstract] OR BASEBALL[Title/Abstract] OR SKIING[Title/Abstract] OR SKATING[Title/Abstract] OR DIVING[Title/Abstract] OR SWIMMING[Title/Abstract] OR VOLLEYBALL[Title/Abstract] OR HOCKEY[Title/Abstract] OR CRICKET[Title/Abstract] OR WALKING[Title/Abstract] OR NETBALL OR "TABLE TENNIS"[Title/Abstract] OR SPORT*[Title/Abstract]))) OR (((((((("Exercise"[Mesh] OR "Circuit-Based Exercise"[Mesh] OR "Plyometric Exercise"[Mesh] OR "Resistance Training"[Mesh] OR "High-Intensity Interval Training"[Mesh])) OR "Circuit-Based Exercise"[Mesh]) OR "Soccer"[Mesh]) OR walking[MeSH Terms]) OR "Walking"[Mesh]) OR "Sports"[Mesh]) OR "Walking"[Mesh]))) AND English[lang]) AND (English[lang])

Scopus search terms

((TITLE-ABS-KEY (pomegranate* OR blueberr* OR bilberr* OR blackcurrant* OR cocoa OR "gr een tea*" OR "black tea*" OR quercetin* OR grape* OR "red wine*" OR beetroot* OR soy* O

R polyphenol* OR flavonoid* OR anthocyanin* OR flavones* OR isoflavones* OR flavanols O R cherry*) OR TITLE-ABS-KEY (resveratrol OR picied* OR benzoic* OR cinnamic*))) AND ((TI TLE-ABS-KEY (exercise* OR "resistance training" OR "eccentric training" OR weightlifting OR "cir cuit training" OR "weight training" OR "plyometric training" OR cycling OR running OR soccer O R netball OR table AND tennis OR rugby OR boxing OR basketball OR gymnastics) OR TITLE-A BS-KEY (golf OR wrestling OR "martial art*" OR athletics OR tennis OR squash OR badminto n OR baseball OR skiing OR skating OR diving OR swimming OR volleyball OR hockey OR cric ket OR walking)))

Amed search terms

(pomegranate* or blueberr* or bilberr* or blackurrant* or cherr* or cocoa* or "green tea" or "black tea" or quercetin* or grape* or "red wine" or beetroot* or polyphenol* or flavonoid* or soy* or anthocyanin* or flavones* or isoflavones* or flavanols* or resveratrol* or Picied* or benzoic* or Cinnamic*).ti. or (pomegranate* or blueberr* or bilberr* or blackurrant* or cherr* or cocoa* or "green tea" or "black tea" or quercetin* or grape* or "red wine" or beetroot* or polyphenol* or flavonoid* or soy* or anthocyanin* or flavones* or isoflavones* or flavanols* or resveratrol* or Picied* or benzoic* or Cinnamic*).ab.

(exercis* or "mechanical exercise" or "metabolic exercise" or "resistance training" or " eccentric training" or weightlifting or "circuit training" or " weight training" or "plyometric training" or cycling or netball or tennis or soccer or rugby or basketball or boxing or gymnastics or golf or wrestling or "martial art*" or athletics or tennis or squash or badminton or baseball or skiing or skating or diving or swimming or volleyball or hockey or cricket or walking or sport*).ti. or (exercis* or "mechanical exercise" or "metabolic exercise" or "resistance training" or " eccentric training" or weightlifting or "circuit training" or " weight training" or "plyometric training" or cycling or netball or tennis or soccer or rugby or basketball or boxing or gymnastics or golf or wrestling or "martial art*" or athletics or tennis or squash or badminton or baseball or skiing or skating or diving or swimming or volleyball or hockey or cricket or walking or sport*).ab.

International Clinical Trials search strategy

pomegranate OR blueberr* OR cherry OR cherries OR blackcurrant* OR tea OR teas OR cocoa OR beetroot OR grape OR grapes OR wine OR wines OR soy OR soya OR quercetin OR polyphenol* OR flavonoid* OR anthocyanin* OR flavones* OR isoflavones* OR flavanols* OR reservatrol* OR picied OR benzoic OR cinnamic*

UK Clinical Trials search strategy

((pomegranate* OR blueberr* OR cherr* OR blackcurrant* OR tea* OR cocoa OR beetroot OR grape* OR "red wine*" OR soy* OR quercetin OR polyphenol* OR flavonoid* OR anthocyanin* OR flavones* OR isoflavones* OR flavanols* OR reservatrol* OR picied OR benzoic OR cinnamic*) AND (exercise* OR training OR weightlifting OR cycling OR running OR sport* OR soccer OR rugby OR boxing OR basketball OR gymnastics OR golf OR wrestling OR "martial art*"OR athletics OR tennis OR squash OR badminton OR baseball OR skiing OR skating OR diving OR swimming OR volleyball OR hockey OR cricket OR walking))
