# Supplementary material for: Effect of Polyphenol-Rich Foods, Juices, and Concentrates on Recovery from Exercise Induced Muscle Damage: A Systematic Review and Meta-Analysis
Source: Nutrients. 2021 Aug 27;13(9):2988. doi: 10.3390/nu13092988 (PMC8465563; doi:10.3390/nu13092988)
Supplement: Supplementary file 1 [file nutrients-13-02988-s001.zip › Supplementary File 10 Polyphenol restriction_edited.pdf]

(A)

Study or Subgroup	Std. Mean Difference	SE	Polyphenol Total	Placebo Total	Weight	Std. Mean Difference IV, Random, 95% CI
**27.2.1 Restricted**						
Bell 2015	−0.25	0.5	8	8	3.8%	−0.25 [−1.23, 0.73]
Bell 2016	0.46	0.51	8	8	3.7%	0.46 [−0.54, 1.46]
Clifford 2016b (1)	0.03	0.39	20	10	5.2%	0.03 [−0.73, 0.79]
McLeay 2012 (2)	0.09	0.32	10	10	6.3%	0.09 [−0.54, 0.72]
**Subtotal (95% CI)**			**46**	**36**	**19.1%**	**0.08 [−0.32, 0.47]**
Heterogeneity: Tau² = 0.00; Chi² = 1.01, df = 3 (P = 0.80); I² = 0%						
Test for overall effect: Z = 0.38 (P = 0.71)						
**27.2.2 Non-Restricted**						
Bowtell 2011	0.58	0.36	9	9	5.6%	0.58 [−0.13, 1.29]
Brown 2018	0.09	0.45	10	10	4.4%	0.09 [−0.79, 0.97]
Clifford 2016a	0.09	0.45	10	10	4.4%	0.09 [−0.79, 0.97]
Clifford 2017a	0.44	0.35	17	17	5.8%	0.44 [−0.25, 1.13]
Clifford 2017b	−0.11	0.45	10	10	4.4%	−0.11 [−0.99, 0.77]
Connolly 2006	1.99	0.46	14	14	4.3%	1.99 [1.09, 2.89]
Daab et al. 2020	1.55	0.41	13	13	4.9%	1.55 [0.75, 2.35]
Howatson 2010	0.84	0.47	10	10	4.2%	0.84 [−0.08, 1.76]
Lamb et al. 2019 (3)	−0.12	0.37	22	11	5.5%	−0.12 [−0.85, 0.61]
Lima et al. 2019	0.37	0.37	15	15	5.5%	0.37 [−0.36, 1.10]
Machin et al. 2014 (4)	0.28	0.23	30	15	8.1%	0.28 [−0.17, 0.73]
Morgan et al. 2018	0.05	0.32	10	10	6.3%	0.05 [−0.58, 0.68]
Peschek 2014 (5)	0	0.33	8	8	6.1%	0.00 [−0.65, 0.65]
Quinlan 2020	1.3	0.5	10	10	3.8%	1.30 [0.32, 2.28]
Trombold 2011 (6)	0.33	0.25	17	17	7.7%	0.33 [−0.16, 0.82]
**Subtotal (95% CI)**			**205**	**179**	**80.9%**	**0.47 [0.19, 0.74]**
Heterogeneity: Tau² = 0.16; Chi² = 31.40, df = 14 (P = 0.005); I² = 55%						
Test for overall effect: Z = 3.27 (P = 0.001)						
**Total (95% CI)**			**251**	**215**	**100.0%**	**0.39 [0.15, 0.62]**
Heterogeneity: Tau² = 0.13; Chi² = 34.69, df = 18 (P = 0.01); I² = 48%						
Test for overall effect: Z = 3.22 (P = 0.001)						
Test for subgroup differences: Chi² = 2.46, df = 1 (P = 0.12), I² = 59.3%						

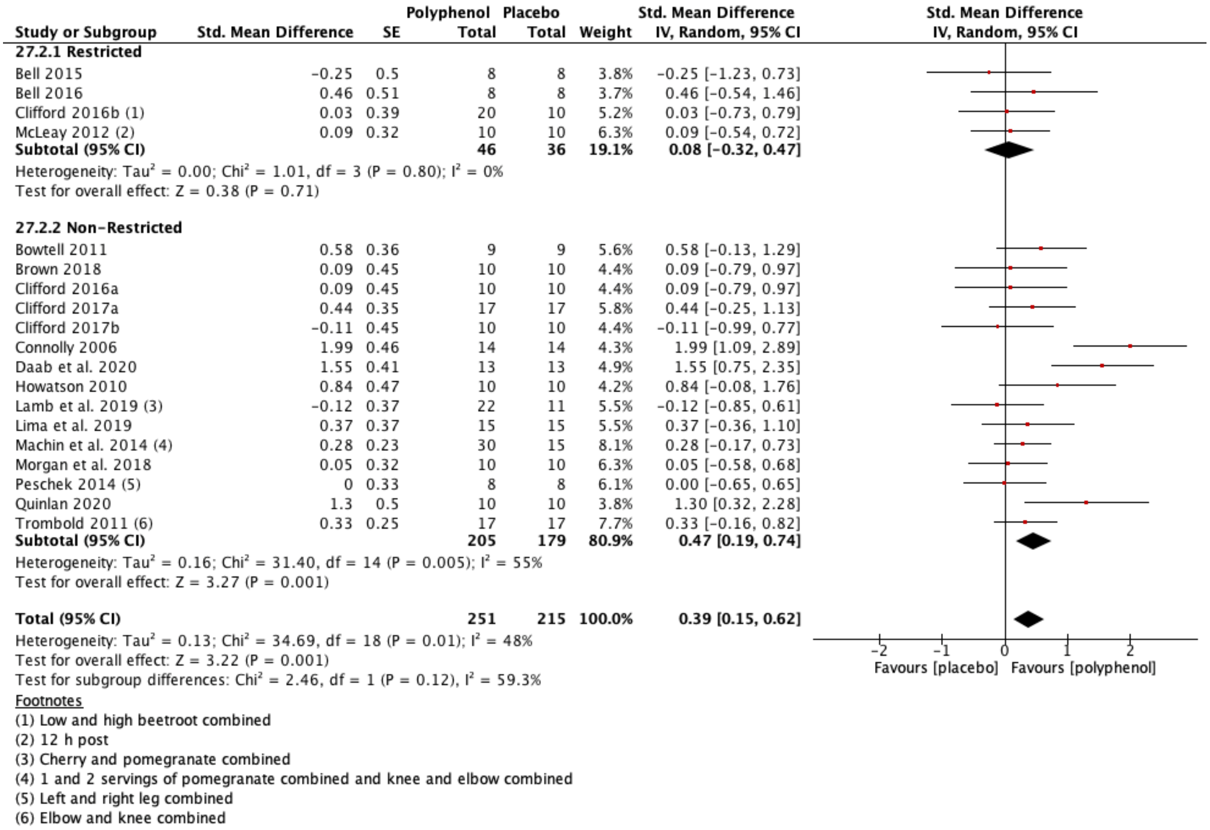

Footnotes
(1) Low and high beetroot combined
(2) 12 h post
(3) Cherry and pomegranate combined
(4) 1 and 2 servings of pomegranate combined and knee and elbow combined
(5) Left and right leg combined
(6) Elbow and knee combined

(B)

Study or Subgroup	Std. Mean Difference	SE	Polyphenol Total	Placebo Total	Weight	Std. Mean Difference IV, Random, 95% CI
**27.3.1 Restricted**						
Bell 2015	0.13	0.5	8	8	3.3%	0.13 [−0.85, 1.11]
Bell 2016	0.49	0.51	8	8	3.2%	0.49 [−0.51, 1.49]
Clifford 2016b (1)	0.16	0.39	20	10	4.9%	0.16 [−0.60, 0.92]
McLeay 2012 (2)	0.58	0.34	10	10	5.9%	0.58 [−0.09, 1.25]
**Subtotal (95% CI)**			**46**	**36**	**17.3%**	**0.37 [−0.04, 0.78]**
Heterogeneity: Tau² = 0.00; Chi² = 0.96, df = 3 (P = 0.81); I² = 0%						
Test for overall effect: Z = 1.76 (P = 0.08)						
**27.3.2 Non-Restricted**						
Bowtell 2011	0.42	0.35	9	9	5.7%	0.42 [−0.27, 1.11]
Brown 2018	0.24	0.45	10	10	3.9%	0.24 [−0.64, 1.12]
Clifford 2016a	0.12	0.45	10	10	3.9%	0.12 [−0.76, 1.00]
Clifford 2017a	0.23	0.34	17	17	5.9%	0.23 [−0.44, 0.90]
Clifford 2017b	−0.07	0.45	10	10	3.9%	−0.07 [−0.95, 0.81]
Connolly 2006	1.61	0.4	14	14	4.7%	1.61 [0.83, 2.39]
Daab et al. 2020	1.06	0.35	13	13	5.7%	1.06 [0.37, 1.75]
Howatson 2010	0.8	0.47	10	10	3.7%	0.80 [−0.12, 1.72]
Lamb et al. 2019 (3)	−0.06	0.37	22	11	5.3%	−0.06 [−0.79, 0.67]
Lima et al. 2019	0.53	0.37	15	15	5.3%	0.53 [−0.20, 1.26]
Machin et al. 2014 (4)	0.11	0.22	30	15	9.7%	0.11 [−0.32, 0.54]
Morgan et al. 2018	0.4	0.33	10	10	6.1%	0.40 [−0.25, 1.05]
Peschek 2014 (5)	0.57	0.27	8	8	7.9%	0.57 [0.04, 1.10]
Quinlan 2020	1.7	0.54	10	10	2.9%	1.70 [0.64, 2.76]
Trombold 2011 (6)	0.56	0.26	17	17	8.2%	0.56 [0.05, 1.07]
**Subtotal (95% CI)**			**205**	**179**	**82.7%**	**0.51 [0.27, 0.75]**
Heterogeneity: Tau² = 0.09; Chi² = 24.52, df = 14 (P = 0.04); I² = 43%						
Test for overall effect: Z = 4.18 (P < 0.0001)						
**Total (95% CI)**			**251**	**215**	**100.0%**	**0.48 [0.28, 0.67]**
Heterogeneity: Tau² = 0.06; Chi² = 25.71, df = 18 (P = 0.11); I² = 30%						
Test for overall effect: Z = 4.74 (P < 0.00001)						
Test for subgroup differences: Chi² = 0.34, df = 1 (P = 0.56), I² = 0%						

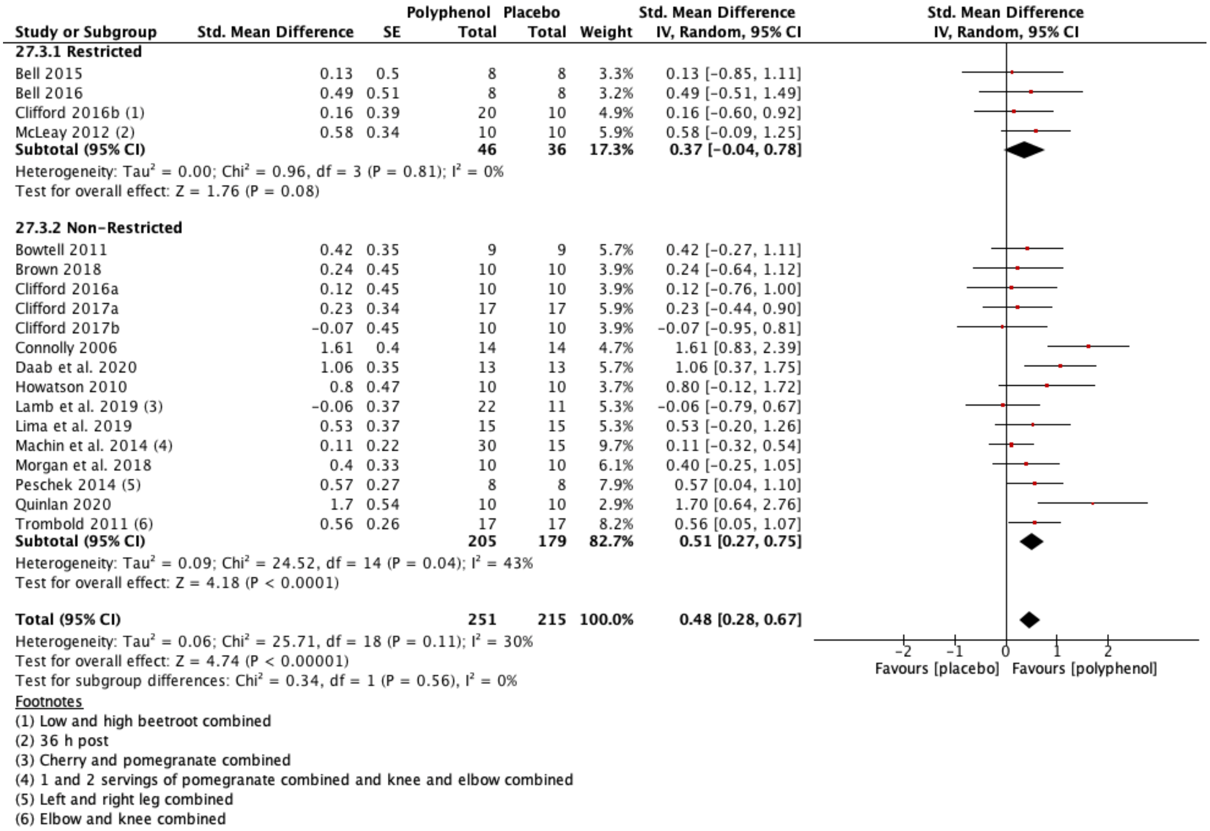

Footnotes
(1) Low and high beetroot combined
(2) 36 h post
(3) Cherry and pomegranate combined
(4) 1 and 2 servings of pomegranate combined and knee and elbow combined
(5) Left and right leg combined
(6) Elbow and knee combined

(C)

Study or Subgroup	Std. Mean Difference	SE	Polyphenol Total	Placebo Total	Weight	Std. Mean Difference IV, Random, 95% CI
**27.4.1 Restricted**						
Bell 2015	0.2	0.5	8	8	3.1%	0.20 [−0.78, 1.18]
Bell 2016	0.28	0.51	8	8	3.0%	0.28 [−0.72, 1.28]
Clifford 2016b (1)	0.07	0.39	20	10	5.1%	0.07 [−0.69, 0.83]
McLeay 2012 (2)	0.1	0.32	10	10	7.6%	0.10 [−0.53, 0.73]
**Subtotal (95% CI)**			**46**	**36**	**18.8%**	**0.14 [−0.26, 0.54]**
Heterogeneity: Tau² = 0.00; Chi² = 0.14, df = 3 (P = 0.99); I² = 0%						
Test for overall effect: Z = 0.67 (P = 0.50)						
**27.4.2 Non-Restrcited**						
Brown 2018	0.01	0.45	10	10	3.8%	0.01 [−0.87, 0.89]
Clifford 2016a	0.3	0.45	10	10	3.8%	0.30 [−0.58, 1.18]
Clifford 2017b	0.13	0.45	10	10	3.8%	0.13 [−0.75, 1.01]
Connolly 2006	1.18	0.35	14	14	6.3%	1.18 [0.49, 1.87]
Daab et al. 2020	0.44	0.29	13	13	9.2%	0.44 [−0.13, 1.01]
Lamb et al. 2019 (3)	−0.05	0.37	22	11	5.7%	−0.05 [−0.78, 0.68]
Lima et al. 2019	0.45	0.37	15	15	5.7%	0.45 [−0.28, 1.18]
Machin et al. 2014 (4)	0.29	0.22	30	15	16.0%	0.29 [−0.14, 0.72]
Trombold 2011 (5)	0.22	0.17	17	17	26.8%	0.22 [−0.11, 0.55]
**Subtotal (95% CI)**			**141**	**115**	**81.2%**	**0.32 [0.12, 0.52]**
Heterogeneity: Tau² = 0.00; Chi² = 8.35, df = 8 (P = 0.40); I² = 4%						
Test for overall effect: Z = 3.19 (P = 0.001)						
**Total (95% CI)**			**187**	**151**	**100.0%**	**0.29 [0.11, 0.46]**
Heterogeneity: Tau² = 0.00; Chi² = 9.15, df = 12 (P = 0.69); I² = 0%						
Test for overall effect: Z = 3.25 (P = 0.001)						
Test for subgroup differences: Chi² = 0.67, df = 1 (P = 0.41), I² = 0%						

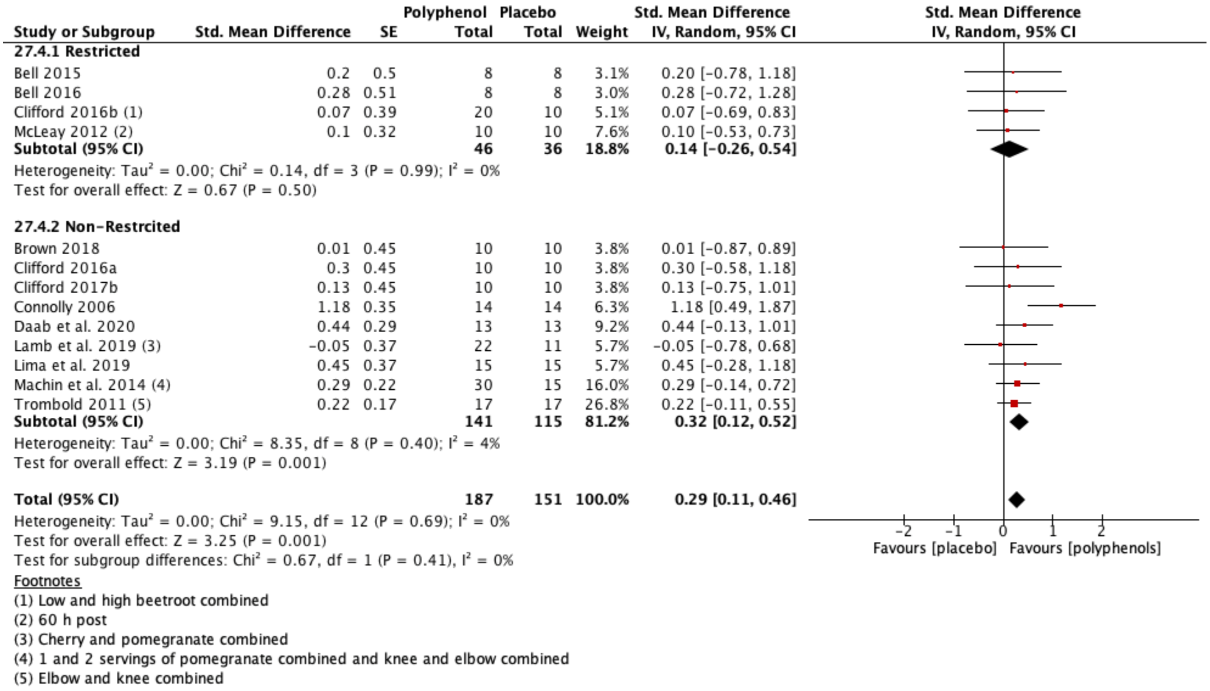

Footnotes
(1) Low and high beetroot combined
(2) 60 h post
(3) Cherry and pomegranate combined
(4) 1 and 2 servings of pomegranate combined and knee and elbow combined
(5) Elbow and knee combined

Supplementary Figure 10a. Comparison of studies based on restriction of dietary polyphenol intake: The effect of polyphenol-rich foods, juices and concentrates on recovery of maximal isometric voluntary contraction (A) 24 hours; (B) 48 hours; (C) 72 hours.

(A)

Study or Subgroup	Std. Mean Difference	SE	Polyphenol Total	Placebo Total	Weight	Std. Mean Difference IV, Random, 95% CI
**28.2.1 Restricted**						
Bell 2015	−0.17	0.5	8	8	2.5%	−0.17 [−1.15, 0.81]
Bell 2016	−0.73	0.52	8	8	2.4%	−0.73 [−1.75, 0.29]
Clifford 2016b (1)	−1.08	0.42	20	10	3.3%	−1.08 [−1.90, −0.26]
Hutchison 2016	−0.3	0.51	8	8	2.5%	−0.30 [−1.30, 0.70]
McLeay 2012 (2)	−0.17	0.32	10	10	4.5%	−0.17 [−0.80, 0.46]
**Subtotal (95% CI)**			**54**	**44**	**15.2%**	**−0.46 [−0.83, −0.08]**
Heterogeneity: $Tau^2 = 0.00$; $Chi^2 = 3.70$, df = 4 (P = 0.45); $I^2 = 0\%$						
Test for overall effect: Z = 2.37 (P = 0.02)						
**28.2.2 Non-Restricted**						
Abbot et al. 2019 (3)	0.11	0.34	10	10	4.2%	0.11 [−0.56, 0.78]
Bowtell 2011 (4)	−0.28	0.34	10	10	4.2%	−0.28 [−0.95, 0.39]
Brown 2018 (5)	−1.09	0.24	10	10	5.9%	−1.09 [−1.56, −0.62]
Clifford 2016a (6)	−0.09	0.45	10	10	3.0%	−0.09 [−0.97, 0.79]
Clifford 2017a	−0.04	0.34	17	17	4.2%	−0.04 [−0.71, 0.63]
Clifford 2017b (7)	−0.47	0.45	10	10	3.0%	−0.47 [−1.35, 0.41]
Connolly 2006 (8)	−0.15	0.19	14	14	7.0%	−0.15 [−0.52, 0.22]
Daab et al. 2020	−1.46	0.4	13	13	3.5%	−1.46 [−2.24, −0.68]
Howatson 2010	0.2	0.45	10	10	3.0%	0.20 [−0.68, 1.08]
Kupusaravic et al. 2019	−0.45	0.33	10	10	4.4%	−0.45 [−1.10, 0.20]
Lamb et al. 2019 (9)	0.61	0.37	24	11	3.8%	0.61 [−0.12, 1.34]
Lima et al. 2019	−0.64	0.38	15	15	3.7%	−0.64 [−1.38, 0.10]
Lynn 2018	0.02	0.46	9	10	2.9%	0.02 [−0.88, 0.92]
Machin et al. 2014 (10)	0.09	0.22	30	15	6.3%	0.09 [−0.34, 0.52]
Morehen 2020	0	0.3	11	11	4.8%	0.00 [−0.59, 0.59]
Morgan et al. 2018	0.15	0.32	10	10	4.5%	0.15 [−0.48, 0.78]
Peschek 2014 (11)	−0.53	0.22	8	8	6.3%	−0.53 [−0.96, −0.10]
Quinlan 2020	−0.52	0.46	10	10	2.9%	−0.52 [−1.42, 0.38]
Trombold 2011 (12)	−0.25	0.18	16	16	7.2%	−0.25 [−0.60, 0.10]
**Subtotal (95% CI)**			**247**	**220**	**84.8%**	**−0.26 [−0.46, −0.05]**
Heterogeneity: $Tau^2 = 0.10$; $Chi^2 = 38.29$, df = 18 (P = 0.004); $I^2 = 53\%$						
Test for overall effect: Z = 2.46 (P = 0.01)						
**Total (95% CI)**			**301**	**264**	**100.0%**	**−0.29 [−0.47, −0.11]**
Heterogeneity: $Tau^2 = 0.09$; $Chi^2 = 42.88$, df = 23 (P = 0.007); $I^2 = 46\%$						
Test for overall effect: Z = 3.14 (P = 0.002)						
Test for subgroup differences: $Chi^2 = 0.83$, df = 1 (P = 0.36), $I^2 = 0\%$						

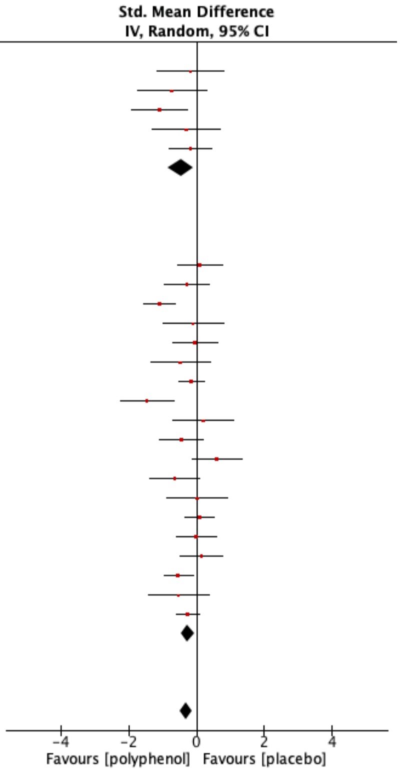

Footnotes
(1) PPT low and high beetroot combined
(2) 12 h post
(3) 12 h post
(4) PPT
(5) PPT and VAS combined
(6) PPT
(7) PPT
(8) PPT and VAS combined
(9) Cherry and pomegranate group combined
(10) 1 and 2 servings of pomegranate combined and knee and elbow combined
(11) PPT and VAS combined
(12) Elbow and knee combined

(B)

Study or Subgroup	Std. Mean Difference	SE	Polyphenol Total	Placebo Total	Weight	Std. Mean Difference IV, Random, 95% CI
**28.3.1 Restricted**						
Bell 2015	−0.31	0.5	8	8	2.6%	−0.31 [−1.29, 0.67]
Bell 2016	−0.98	0.54	8	8	2.3%	−0.98 [−2.04, 0.08]
Clifford 2016b (1)	−0.88	0.41	20	10	3.4%	−0.88 [−1.68, −0.08]
Hutchison 2016	−0.25	0.5	8	8	2.6%	−0.25 [−1.23, 0.73]
McLeay 2012 (2)	0.21	0.32	10	10	4.6%	0.21 [−0.42, 0.84]
**Subtotal (95% CI)**			**54**	**44**	**15.5%**	**−0.38 [−0.85, 0.10]**
Heterogeneity: $Tau^2 = 0.10$; $Chi^2 = 6.12$, df = 4 (P = 0.19); $I^2 = 35\%$						
Test for overall effect: Z = 1.54 (P = 0.12)						
**28.3.2 Non-Restricted**						
Abbot et al. 2019 (3)	0.15	0.44	10	10	3.1%	0.15 [−0.71, 1.01]
Bowtell 2011 (4)	−0.27	0.45	10	10	3.0%	−0.27 [−1.15, 0.61]
Brown 2018 (5)	−1.09	0.29	10	10	5.1%	−1.09 [−1.66, −0.52]
Clifford 2016a (6)	−0.29	0.45	10	10	3.0%	−0.29 [−1.17, 0.59]
Clifford 2017a	−0.53	0.35	17	17	4.2%	−0.53 [−1.22, 0.16]
Clifford 2017b (7)	−0.48	0.46	10	10	2.9%	−0.48 [−1.38, 0.42]
Connolly 2006 (8)	−0.29	0.19	14	14	7.2%	−0.29 [−0.66, 0.08]
Daab et al. 2020	−0.55	0.3	13	13	5.0%	−0.55 [−1.14, 0.04]
Howatson 2010	0.34	0.45	10	10	3.0%	0.34 [−0.54, 1.22]
Kupusaravic et al. 2019	−0.25	0.32	10	10	4.6%	−0.25 [−0.88, 0.38]
Lamb et al. 2019 (9)	0.66	0.38	24	11	3.8%	0.66 [−0.08, 1.40]
Lima et al. 2019	−1.21	0.4	15	15	3.5%	−1.21 [−1.99, −0.43]
Lynn 2018	0.49	0.47	9	10	2.8%	0.49 [−0.43, 1.41]
Machin et al. 2014 (10)	0	0.22	30	15	6.5%	0.00 [−0.43, 0.43]
Morehen 2020	0.31	0.31	11	11	4.8%	0.31 [−0.30, 0.92]
Morgan et al. 2018	−0.03	0.32	10	10	4.6%	−0.03 [−0.66, 0.60]
Peschek 2014 (11)	−0.22	0.2	8	8	7.0%	−0.22 [−0.61, 0.17]
Quinlan 2020	−1.05	0.48	10	10	2.7%	−1.05 [−1.99, −0.11]
Trombold 2011 (12)	−0.39	0.18	17	17	7.5%	−0.39 [−0.74, −0.04]
**Subtotal (95% CI)**			**248**	**221**	**84.5%**	**−0.26 [−0.46, −0.06]**
Heterogeneity: $Tau^2 = 0.09$; $Chi^2 = 35.23$, df = 18 (P = 0.009); $I^2 = 49\%$						
Test for overall effect: Z = 2.51 (P = 0.01)						
**Total (95% CI)**			**302**	**265**	**100.0%**	**−0.28 [−0.46, −0.09]**
Heterogeneity: $Tau^2 = 0.08$; $Chi^2 = 41.43$, df = 23 (P = 0.01); $I^2 = 44\%$						
Test for overall effect: Z = 2.97 (P = 0.003)						
Test for subgroup differences: $Chi^2 = 0.20$, df = 1 (P = 0.66), $I^2 = 0\%$						

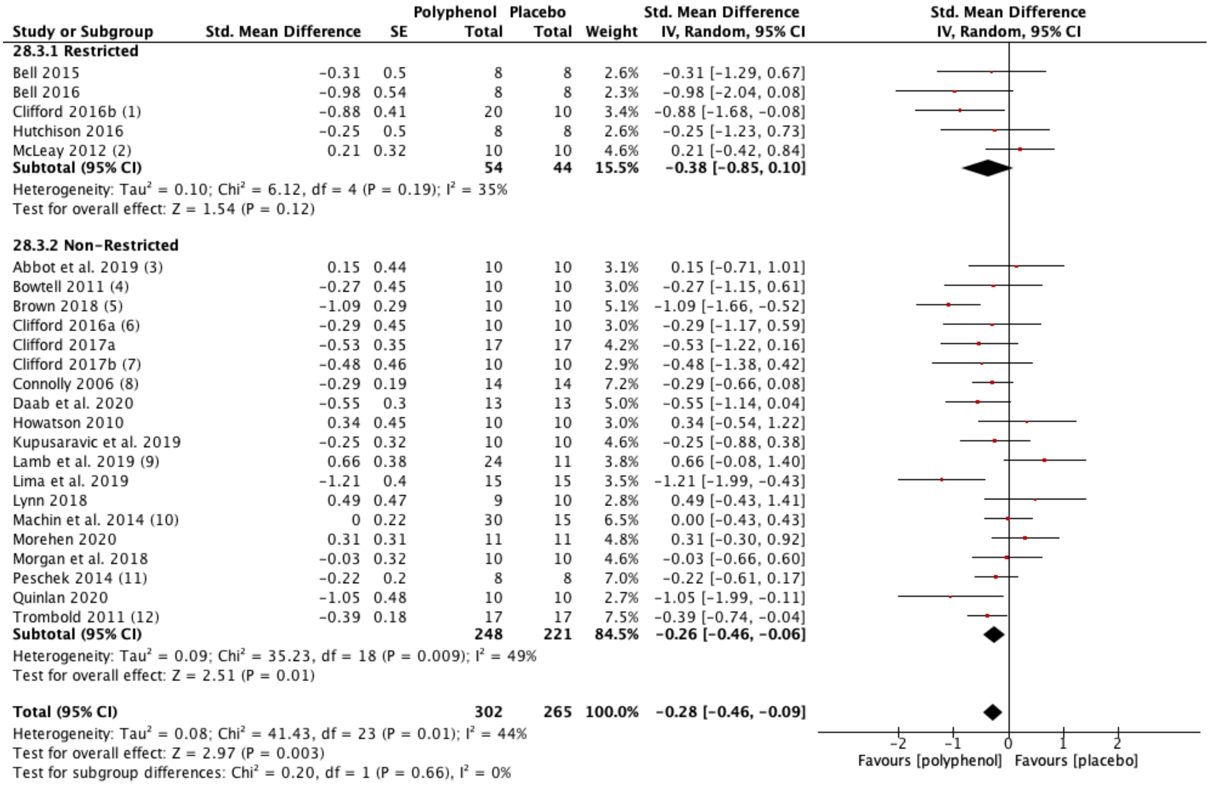

<u>Footnotes</u>
(1) PPT low and high beetroot combined
(2) 36 h post
(3) 36 h post
(4) PPT
(5) PPT and VAS combined
(6) PPT
(7) PPT
(8) PPT and VAS combined
(9) Cherry and pomegranate group combined
(10) 1 and 2 servings of pomegranate combined and knee and elbow combined
(11) PPT and VAS combined
(12) Elbow and knee combined

(C)

Study or Subgroup	Std. Mean Difference	SE	Polyphenol Total	Placebo Total	Weight	Std. Mean Difference IV, Random, 95% CI
**28.4.1 Restricted**						
Bell 2015	−0.57	0.51	8	8	3.8%	−0.57 [−1.57, 0.43]
Bell 2016	−1.11	0.55	8	8	3.4%	−1.11 [−2.19, −0.03]
Clifford 2016b (1)	−0.68	0.4	20	10	5.3%	−0.68 [−1.46, 0.10]
Hutchison 2016	−0.14	0.5	8	8	3.9%	−0.14 [−1.12, 0.84]
McLeay 2012 (2)	0.08	0.32	10	10	6.8%	0.08 [−0.55, 0.71]
**Subtotal (95% CI)**			**54**	**44**	**23.1%**	**−0.39 [−0.80, 0.03]**
Heterogeneity: Tau² = 0.04; Chi² = 4.75, df = 4 (P = 0.31); I² = 16%						
Test for overall effect: Z = 1.82 (P = 0.07)						
**28.4.2 Non-Restricted**						
Abbot et al. 2019 (3)	−0.2	0.33	10	10	6.6%	−0.20 [−0.85, 0.45]
Brown 2018 (4)	−1.35	0.25	10	10	8.6%	−1.35 [−1.84, −0.86]
Clifford 2016a (5)	−0.27	0.45	10	10	4.5%	−0.27 [−1.15, 0.61]
Clifford 2017b (6)	−0.5	0.45	10	10	4.5%	−0.50 [−1.38, 0.38]
Connolly 2006 (7)	−0.31	0.27	14	14	8.1%	−0.31 [−0.84, 0.22]
Daab et al. 2020	−1.11	0.35	13	13	6.2%	−1.11 [−1.80, −0.42]
Kupusaravic et al. 2019	−0.34	0.33	10	10	6.6%	−0.34 [−0.99, 0.31]
Lamb et al. 2019 (8)	0.08	0.36	24	11	6.0%	0.08 [−0.63, 0.79]
Lima et al. 2019	−0.8	0.38	15	15	5.6%	−0.80 [−1.54, −0.06]
Machin et al. 2014 (9)	−0.15	0.22	30	15	9.5%	−0.15 [−0.58, 0.28]
Trombold 2011 (10)	−0.3	0.18	16	16	10.7%	−0.30 [−0.65, 0.05]
**Subtotal (95% CI)**			**162**	**134**	**76.9%**	**−0.48 [−0.75, −0.20]**
Heterogeneity: Tau² = 0.11; Chi² = 23.20, df = 10 (P = 0.01); I² = 57%						
Test for overall effect: Z = 3.43 (P = 0.0006)						
**Total (95% CI)**			**216**	**178**	**100.0%**	**−0.46 [−0.69, −0.24]**
Heterogeneity: Tau² = 0.09; Chi² = 28.16, df = 15 (P = 0.02); I² = 47%						
Test for overall effect: Z = 4.01 (P < 0.0001)						
Test for subgroup differences: Chi² = 0.13, df = 1 (P = 0.72), I² = 0%						

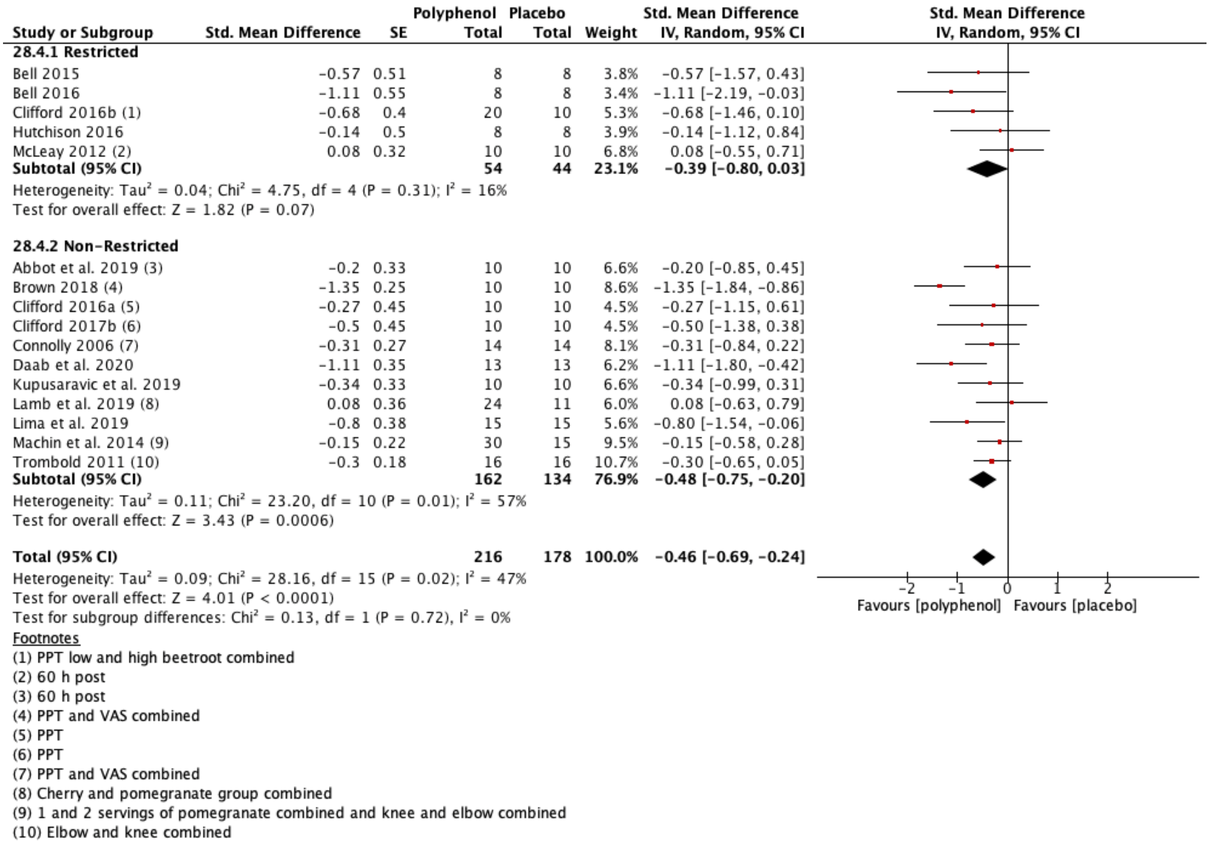

Footnotes
(1) PPT low and high beetroot combined
(2) 60 h post
(3) 60 h post
(4) PPT and VAS combined
(5) PPT
(6) PPT
(7) PPT and VAS combined
(8) Cherry and pomegranate group combined
(9) 1 and 2 servings of pomegranate combined and knee and elbow combined
(10) Elbow and knee combined

Supplementary Figure 10b. Comparison of studies based on restriction of dietary polyphenol intake: The effect of polyphenol-rich foods, juices and concentrates on delayed onset muscle soreness (A) 24 hours; (B) 48 hours; (C) 72 hours.
PPT: Pain pressure threshold
VAS: Visual analogue scale

(A)

Study or Subgroup	Std. Mean Difference	SE	Polyphenol Total	Placebo Total	Weight	Std. Mean Difference IV, Random, 95% CI
**29.2.1 Restricted**						
Bell 2016	1.24	0.56	8	8	6.6%	1.24 [0.14, 2.34]
Clifford 2016b (1)	0.22	0.39	20	10	9.6%	0.22 [−0.54, 0.98]
**Subtotal (95% CI)**			**28**	**18**	**16.2%**	**0.65 [−0.34, 1.64]**
Heterogeneity: Tau^2 = 0.29; Chi^2 = 2.23, df = 1 (P = 0.13); I^2 = 55%						
Test for overall effect: Z = 1.29 (P = 0.20)						
**29.2.2 Non-Restricted**						
Abbot et al. 2019 (2)	−0.24	0.14	10	10	15.5%	−0.24 [−0.51, 0.03]
Brown 2018	0.51	0.46	10	10	8.2%	0.51 [−0.39, 1.41]
Clifford 2016a	0.56	0.46	10	10	8.2%	0.56 [−0.34, 1.46]
Clifford 2017a	0.38	0.34	17	17	10.7%	0.38 [−0.29, 1.05]
Clifford 2017b	0.26	0.45	10	10	8.4%	0.26 [−0.62, 1.14]
Daab et al. 2020	0.41	0.25	13	13	12.9%	0.41 [−0.08, 0.90]
Morgan et al. 2018	0.47	0.23	10	10	13.4%	0.47 [0.02, 0.92]
Quinlan 2020	2.11	0.58	10	10	6.3%	2.11 [0.97, 3.25]
**Subtotal (95% CI)**			**90**	**90**	**83.8%**	**0.44 [0.04, 0.84]**
Heterogeneity: Tau^2 = 0.21; Chi^2 = 24.07, df = 7 (P = 0.001); I^2 = 71%						
Test for overall effect: Z = 2.14 (P = 0.03)						
**Total (95% CI)**			**118**	**108**	**100.0%**	**0.47 [0.11, 0.83]**
Heterogeneity: Tau^2 = 0.20; Chi^2 = 27.62, df = 9 (P = 0.001); I^2 = 67%						
Test for overall effect: Z = 2.53 (P = 0.01)						
Test for subgroup differences: Chi^2 = 0.16, df = 1 (P = 0.69), I^2 = 0%						

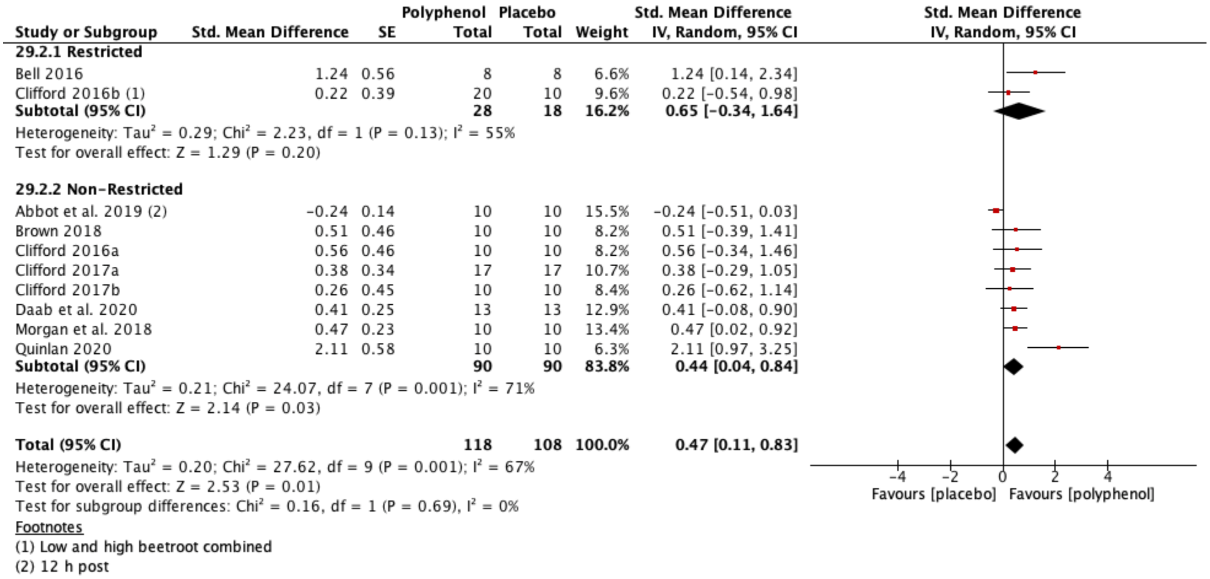

Footnotes
(1) Low and high beetroot combined
(2) 12 h post

(B)

Study or Subgroup	Std. Mean Difference	SE	Polyphenol Total	Placebo Total	Weight	Std. Mean Difference IV, Random, 95% CI
**29.3.1 Restricted**						
Bell 2016	1.34	0.57	8	8	5.7%	1.34 [0.22, 2.46]
Clifford 2016b (1)	0.62	0.4	20	10	8.5%	0.62 [−0.16, 1.40]
**Subtotal (95% CI)**			**28**	**18**	**14.2%**	**0.87 [0.20, 1.53]**
Heterogeneity: Tau^2 = 0.02; Chi^2 = 1.07, df = 1 (P = 0.30); I^2 = 6%						
Test for overall effect: Z = 2.54 (P = 0.01)						
**29.3.2 Non-Restricted**						
Abbot et al. 2019 (2)	−0.1	0.12	10	10	14.7%	−0.10 [−0.34, 0.14]
Brown 2018	1.39	0.51	10	10	6.6%	1.39 [0.39, 2.39]
Clifford 2016a	0.91	0.47	10	10	7.2%	0.91 [−0.01, 1.83]
Clifford 2017a	0.3	0.34	17	17	9.7%	0.30 [−0.37, 0.97]
Clifford 2017b	0.32	0.45	10	10	7.5%	0.32 [−0.56, 1.20]
Daab et al. 2020	0.73	0.36	13	13	9.3%	0.73 [0.02, 1.44]
Morehen 2020	0.14	0.17	11	11	13.7%	0.14 [−0.19, 0.47]
Morgan et al. 2018	0.5	0.28	10	10	11.1%	0.50 [−0.05, 1.05]
Quinlan 2020	1.91	0.56	10	10	5.9%	1.91 [0.81, 3.01]
**Subtotal (95% CI)**			**101**	**101**	**85.8%**	**0.52 [0.16, 0.88]**
Heterogeneity: Tau^2 = 0.18; Chi^2 = 26.90, df = 8 (P = 0.0007); I^2 = 70%						
Test for overall effect: Z = 2.86 (P = 0.004)						
**Total (95% CI)**			**129**	**119**	**100.0%**	**0.58 [0.24, 0.91]**
Heterogeneity: Tau^2 = 0.18; Chi^2 = 31.79, df = 10 (P = 0.0004); I^2 = 69%						
Test for overall effect: Z = 3.39 (P = 0.0007)						
Test for subgroup differences: Chi^2 = 0.79, df = 1 (P = 0.37), I^2 = 0%						

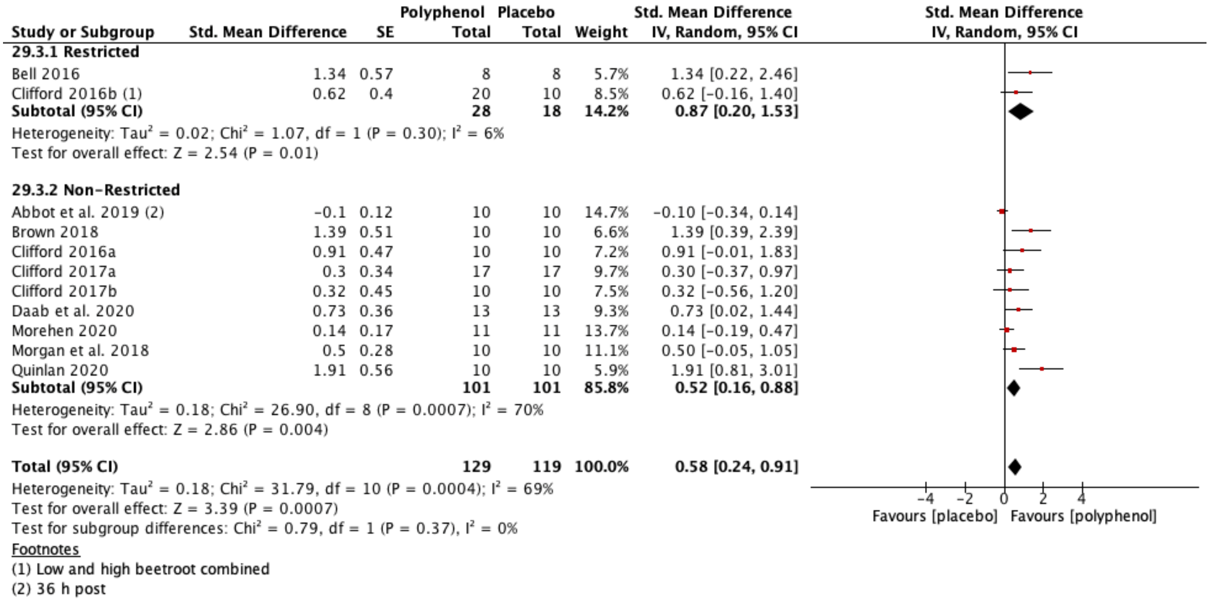

Footnotes
(1) Low and high beetroot combined
(2) 36 h post

(C)

Study or Subgroup	Std. Mean Difference	SE	Polyphenol Total	Placebo Total	Weight	Std. Mean Difference IV, Random, 95% CI
**29.4.1 Restricted**						
Bell 2016	1.49	0.58	8	8	10.7%	1.49 [0.35, 2.63]
Clifford 2016b (1)	0.25	0.39	20	10	14.4%	0.25 [−0.51, 1.01]
**Subtotal (95% CI)**			**28**	**18**	**25.1%**	**0.80 [−0.41, 2.00]**
Heterogeneity: Tau^2 = 0.52; Chi^2 = 3.15, df = 1 (P = 0.08); I^2 = 68%						
Test for overall effect: Z = 1.29 (P = 0.20)						
**29.4.2 Non-Restricted**						
Abbot et al. 2019 (2)	−0.27	0.1	10	10	19.8%	−0.27 [−0.47, −0.07]
Brown 2018	1.02	0.48	10	10	12.5%	1.02 [0.08, 1.96]
Clifford 2016a	1.32	0.51	10	10	12.0%	1.32 [0.32, 2.32]
Clifford 2017b	0.48	0.45	10	10	13.2%	0.48 [−0.40, 1.36]
Daab et al. 2020	0.43	0.25	13	13	17.4%	0.43 [−0.06, 0.92]
**Subtotal (95% CI)**			**53**	**53**	**74.9%**	**0.49 [−0.13, 1.11]**
Heterogeneity: Tau^2 = 0.37; Chi^2 = 22.07, df = 4 (P = 0.0002); I^2 = 82%						
Test for overall effect: Z = 1.56 (P = 0.12)						
**Total (95% CI)**			**81**	**71**	**100.0%**	**0.57 [0.03, 1.10]**
Heterogeneity: Tau^2 = 0.37; Chi^2 = 29.58, df = 6 (P < 0.0001); I^2 = 80%						
Test for overall effect: Z = 2.06 (P = 0.04)						
Test for subgroup differences: Chi^2 = 0.19, df = 1 (P = 0.66), I^2 = 0%						

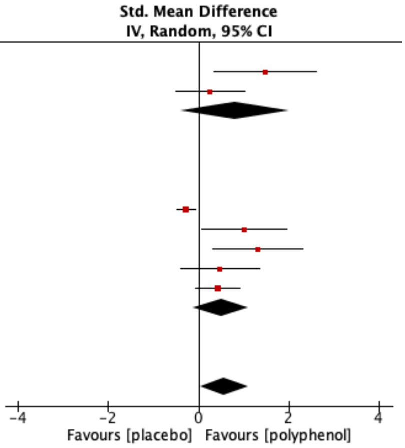

Footnotes
(1) Low and high beetroot combined
(2) 60 h post exercise

Supplementary Figure 10c. Comparison of studies based on restriction of dietary polyphenol intake: The effect of polyphenol-rich foods, juices and concentrates on countermovement jump (A) 24 hours; (B) 48 hours; (C) 72 hours.
