# Supplementary material for: Effect of Polyphenol-Rich Foods, Juices, and Concentrates on Recovery from Exercise Induced Muscle Damage: A Systematic Review and Meta-Analysis
Source: Nutrients. 2021 Aug 27;13(9):2988. doi: 10.3390/nu13092988 (PMC8465563; doi:10.3390/nu13092988)
Supplement: Supplementary file 1 [file nutrients-13-02988-s001.zip › Supplementary File 2 Funnel Plots.pdf]

Supplementary File 2 – Funnel Plots

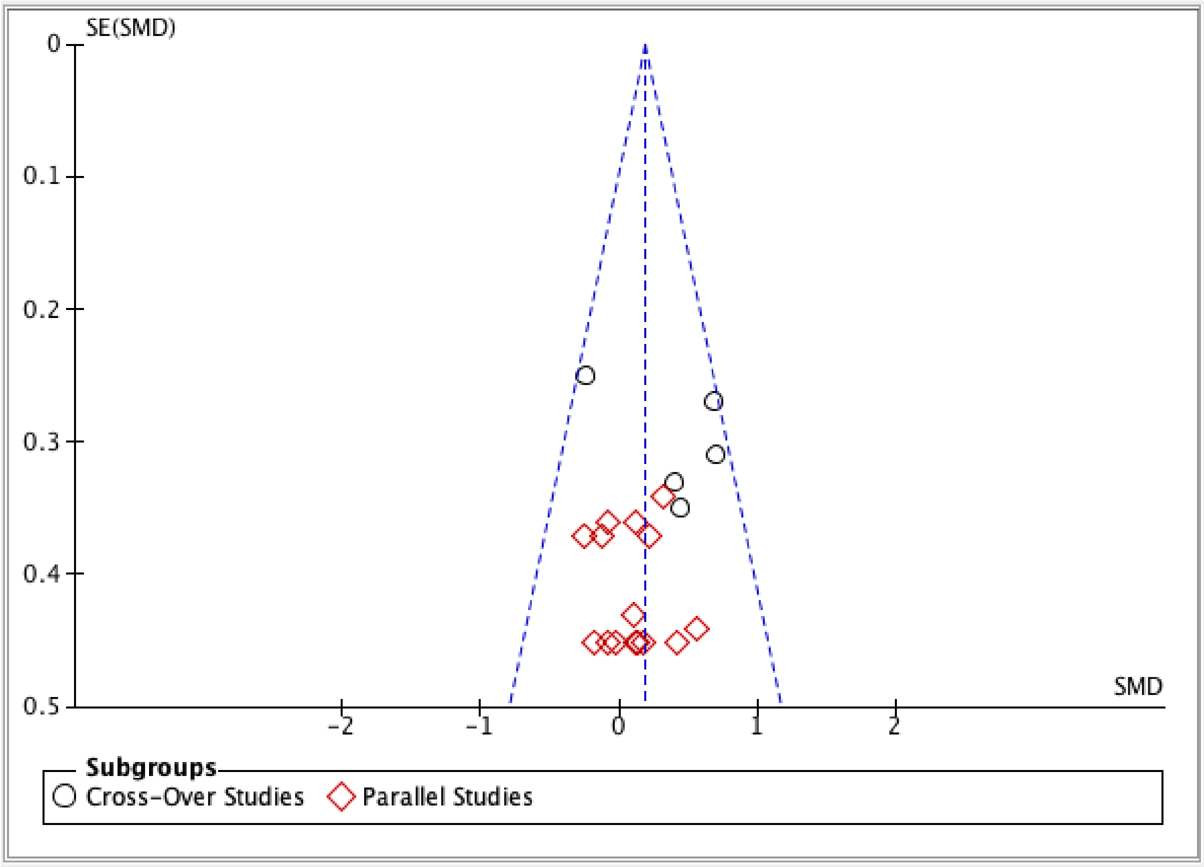

Figure S1a. MIVC Post

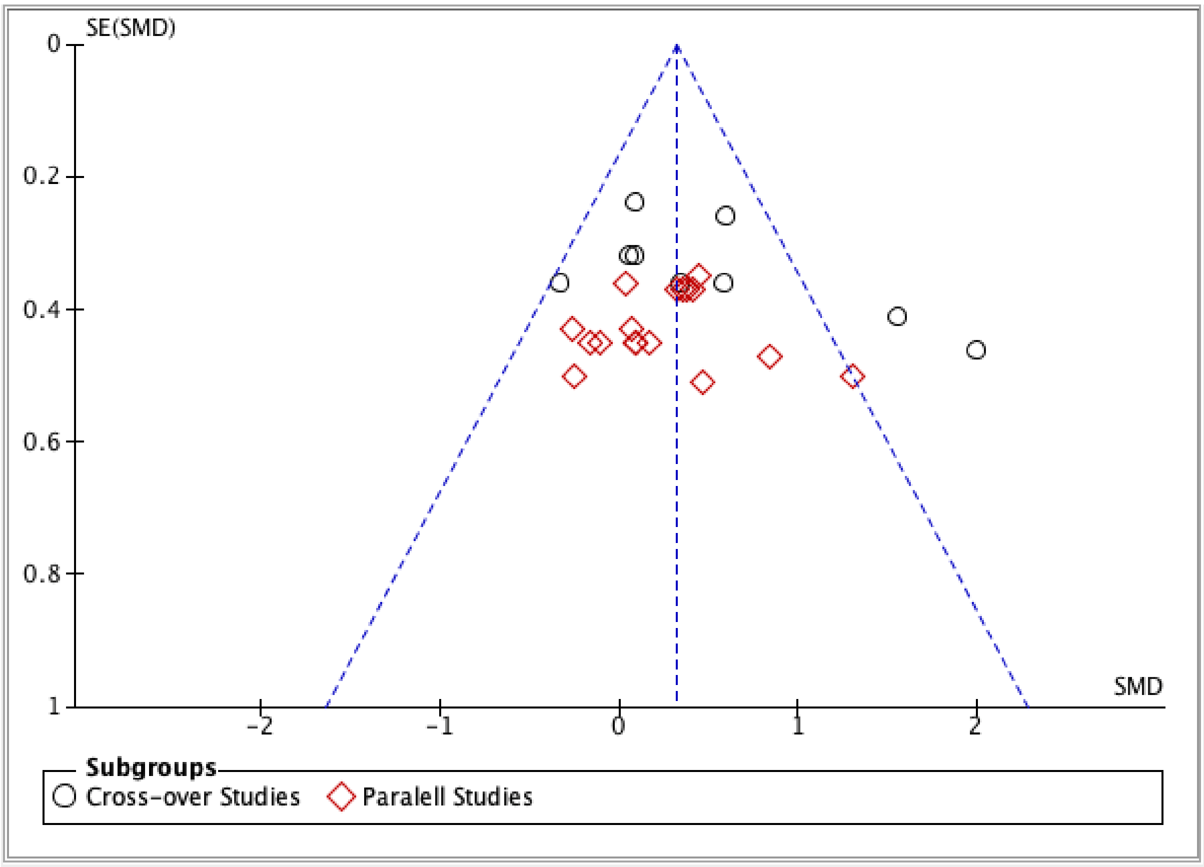

Figure S1b. MIVC 24 hours

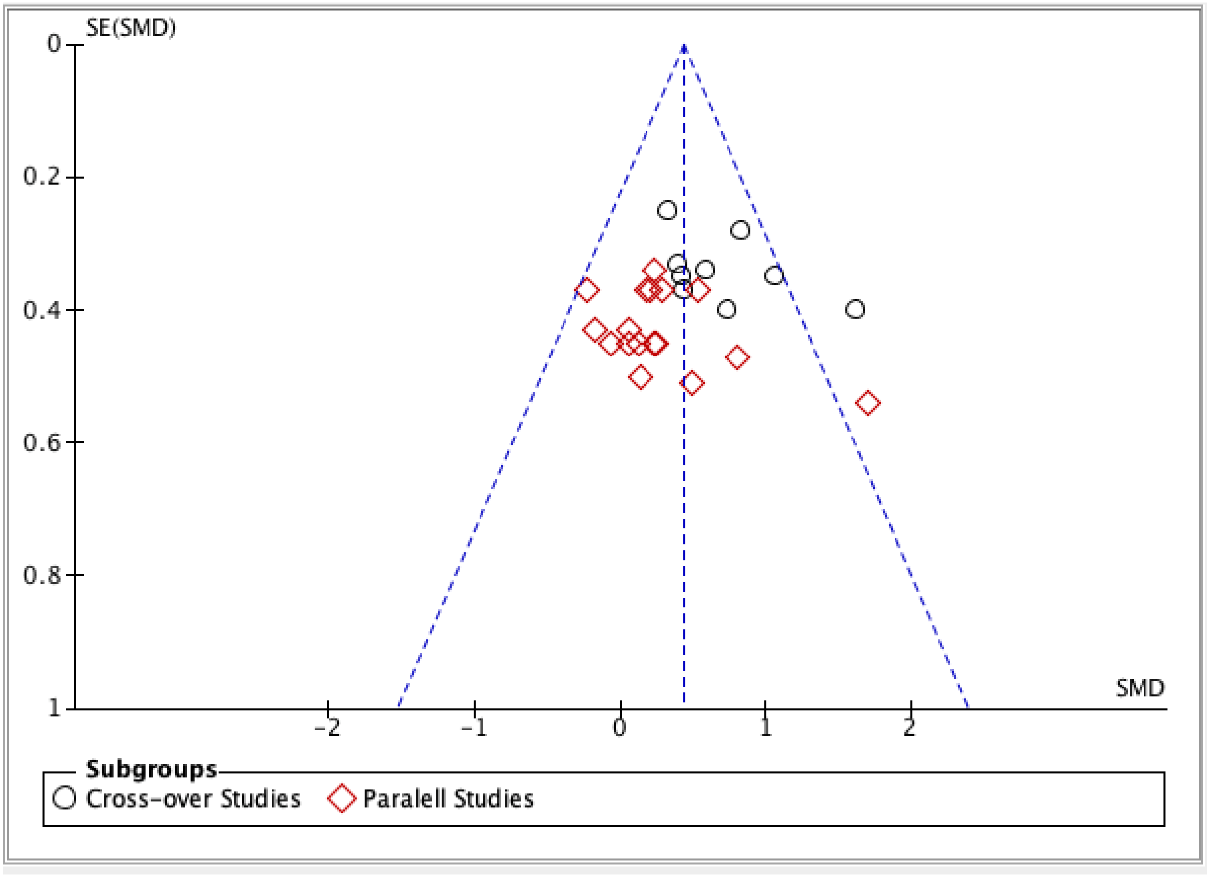

Figure S1c. MIVC 48 hours

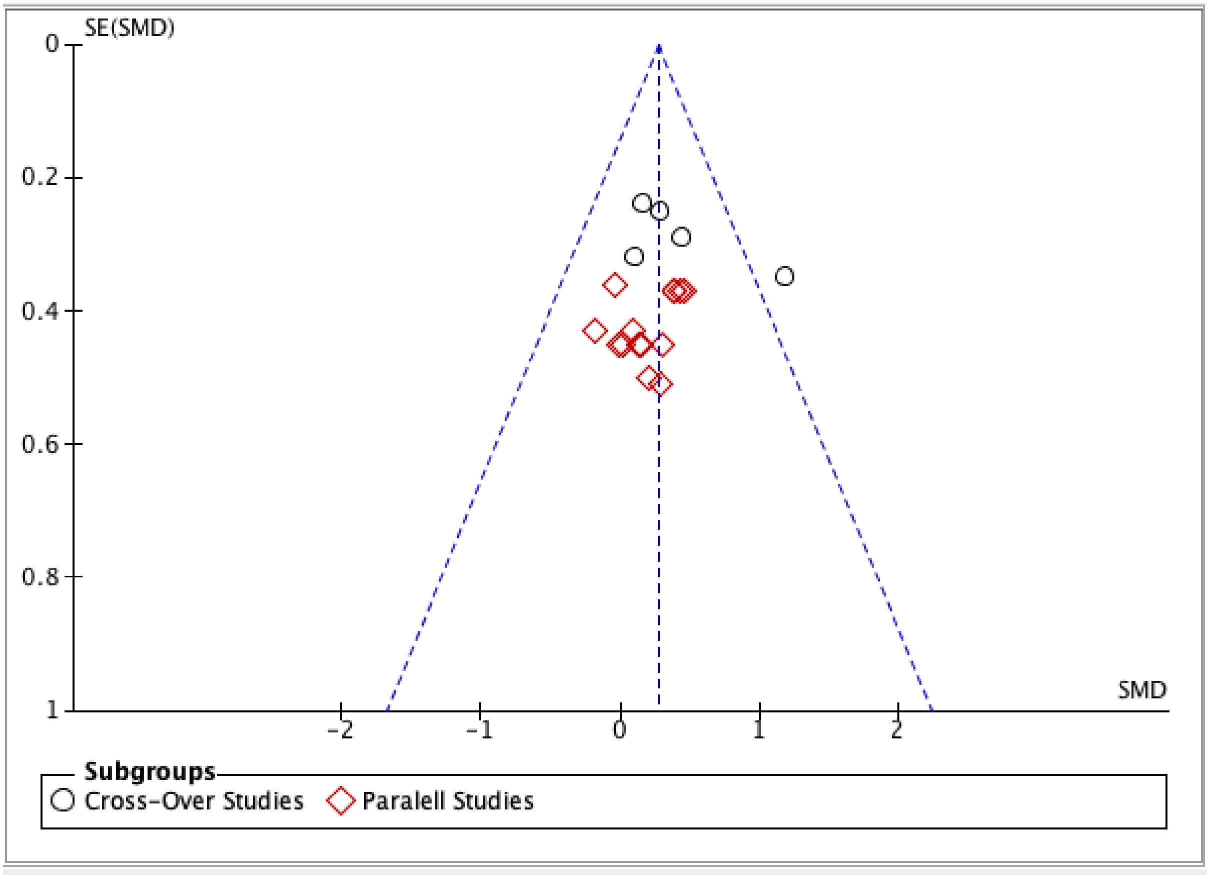

Figure S1d. MIVC 72 hours

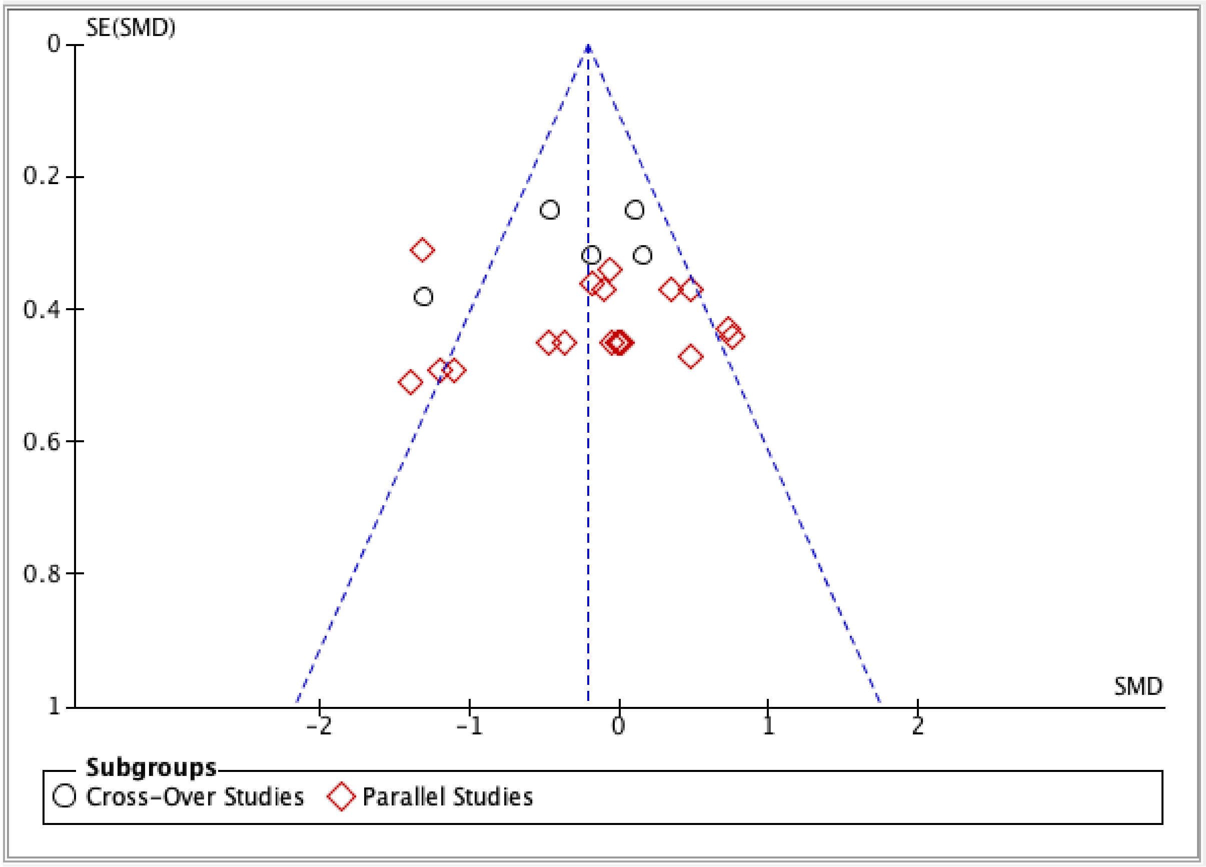

Figure S1e. DOMS Post

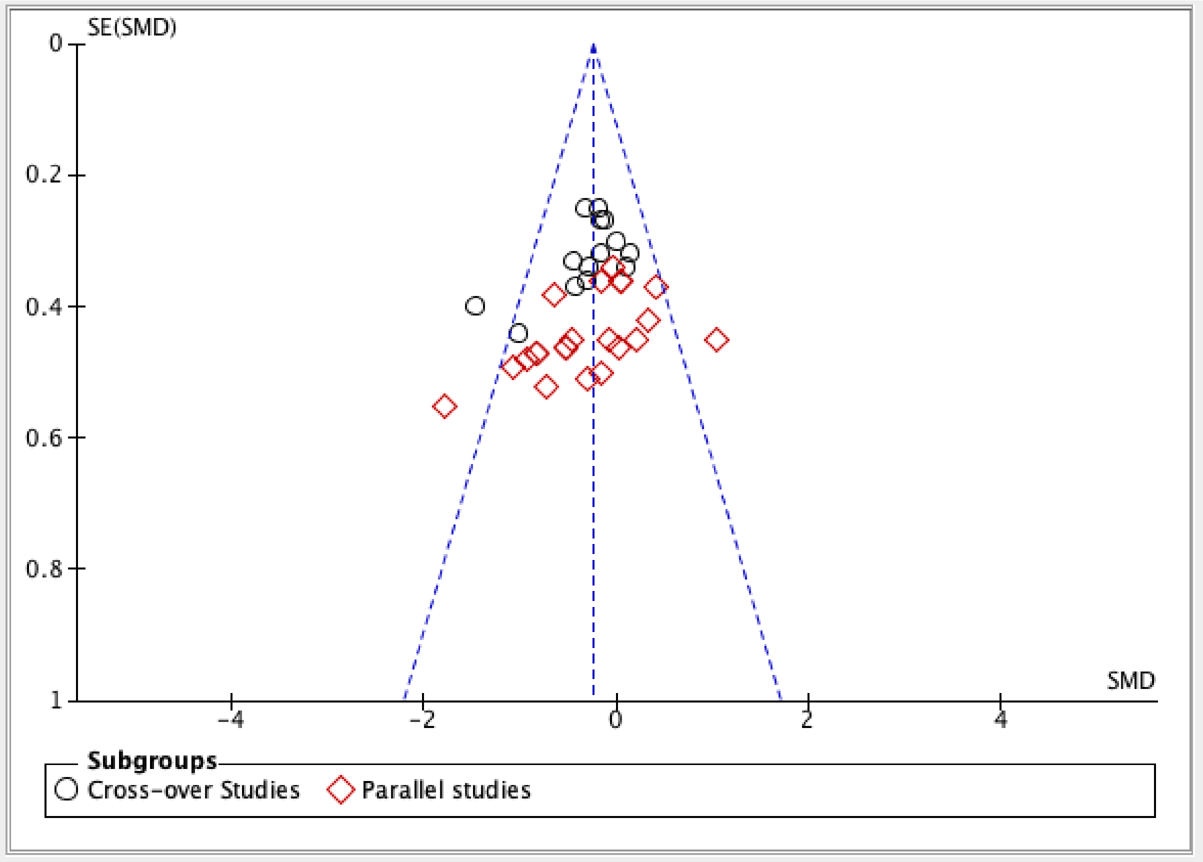

Figure S1f. DOMS 24 hours

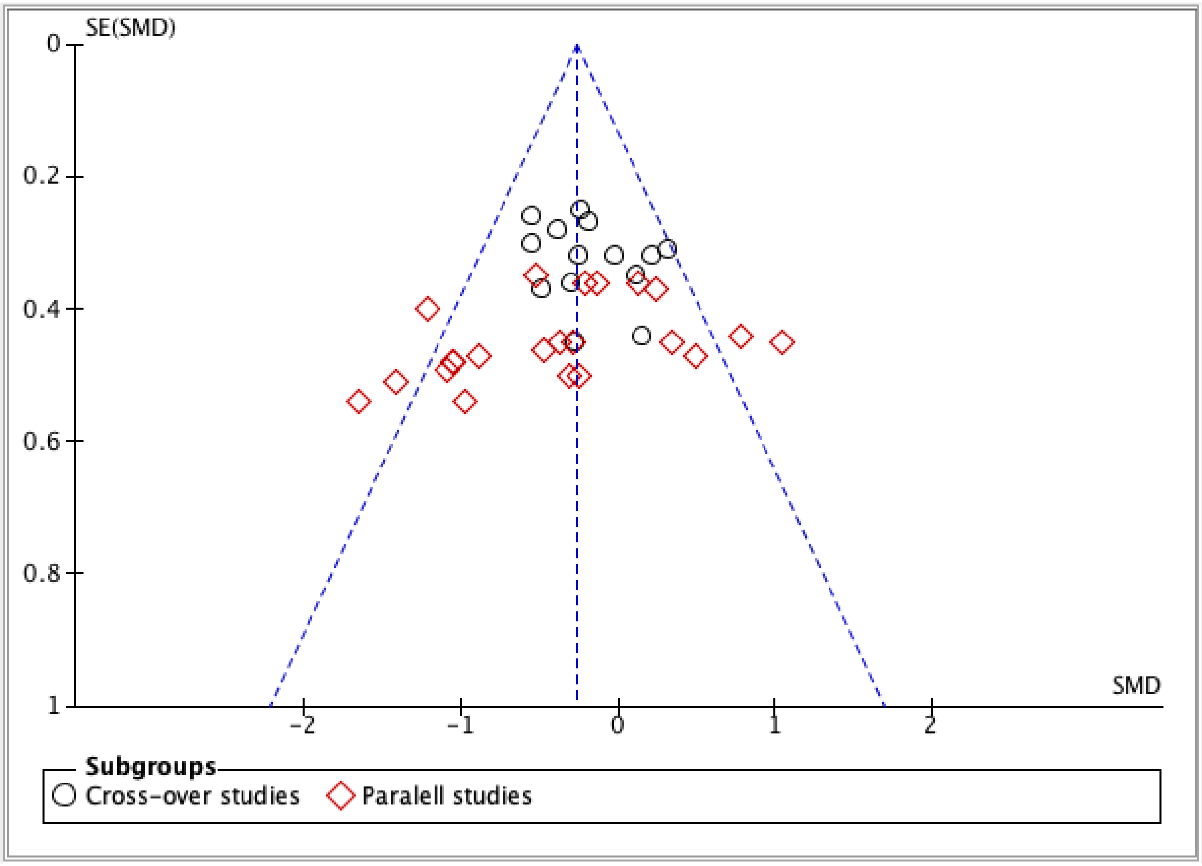

Figure S1g. DOMS 48 hours

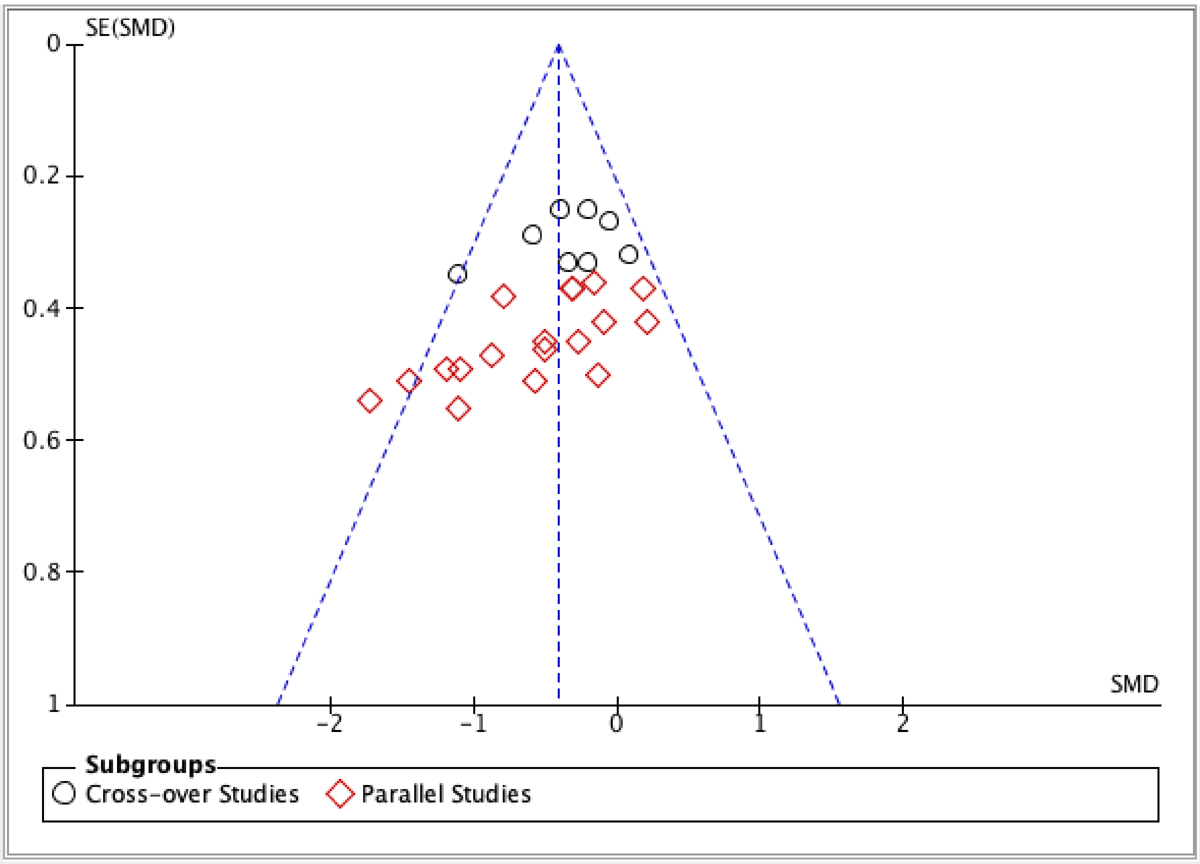

Figure S1h. DOMS 72 hours

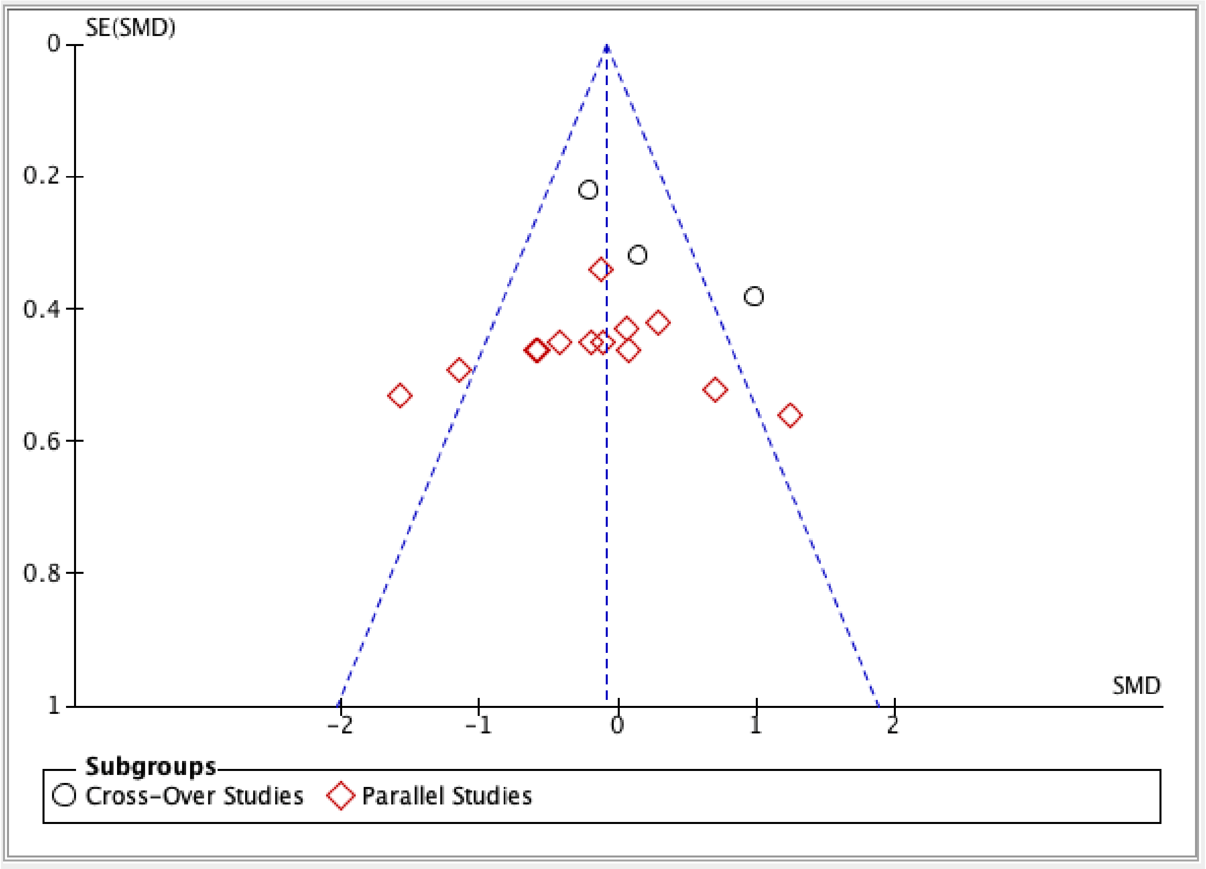

Figure S1i. CK Post

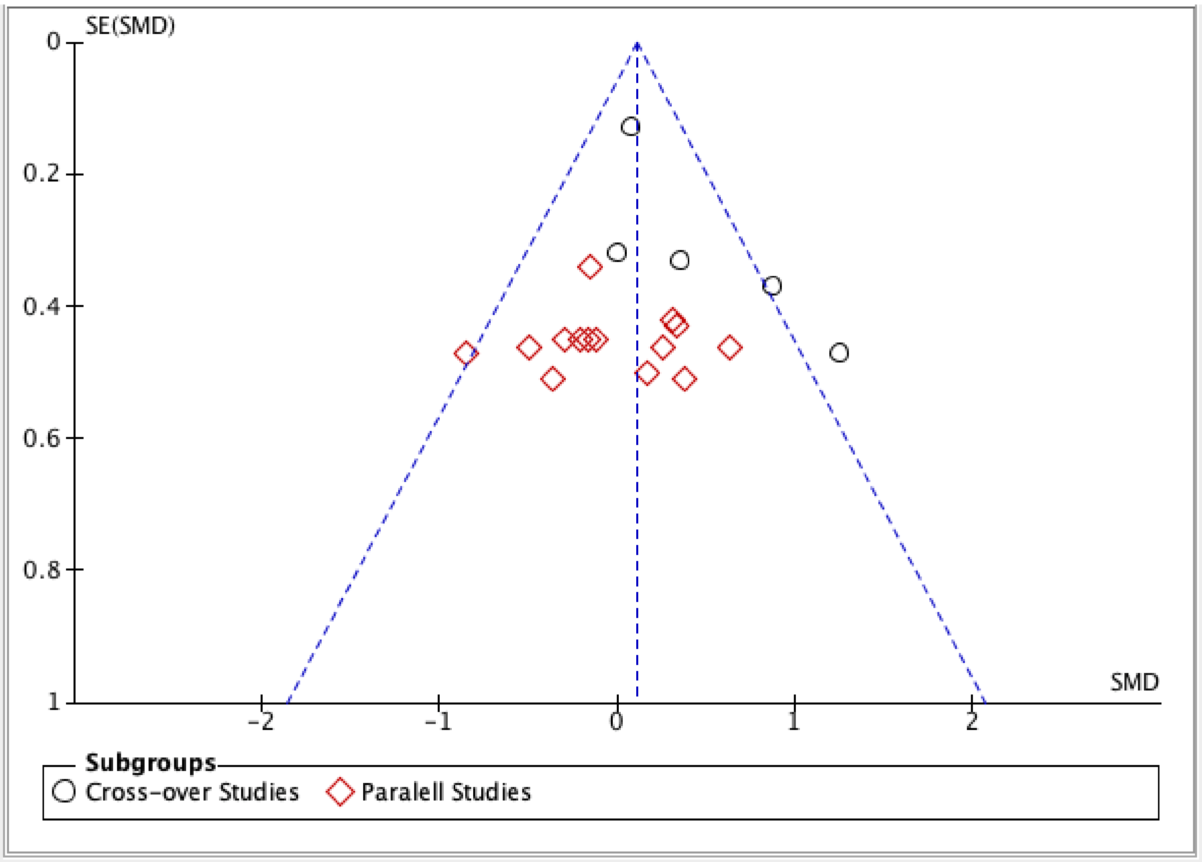

Figure S1j. CK 24 hours

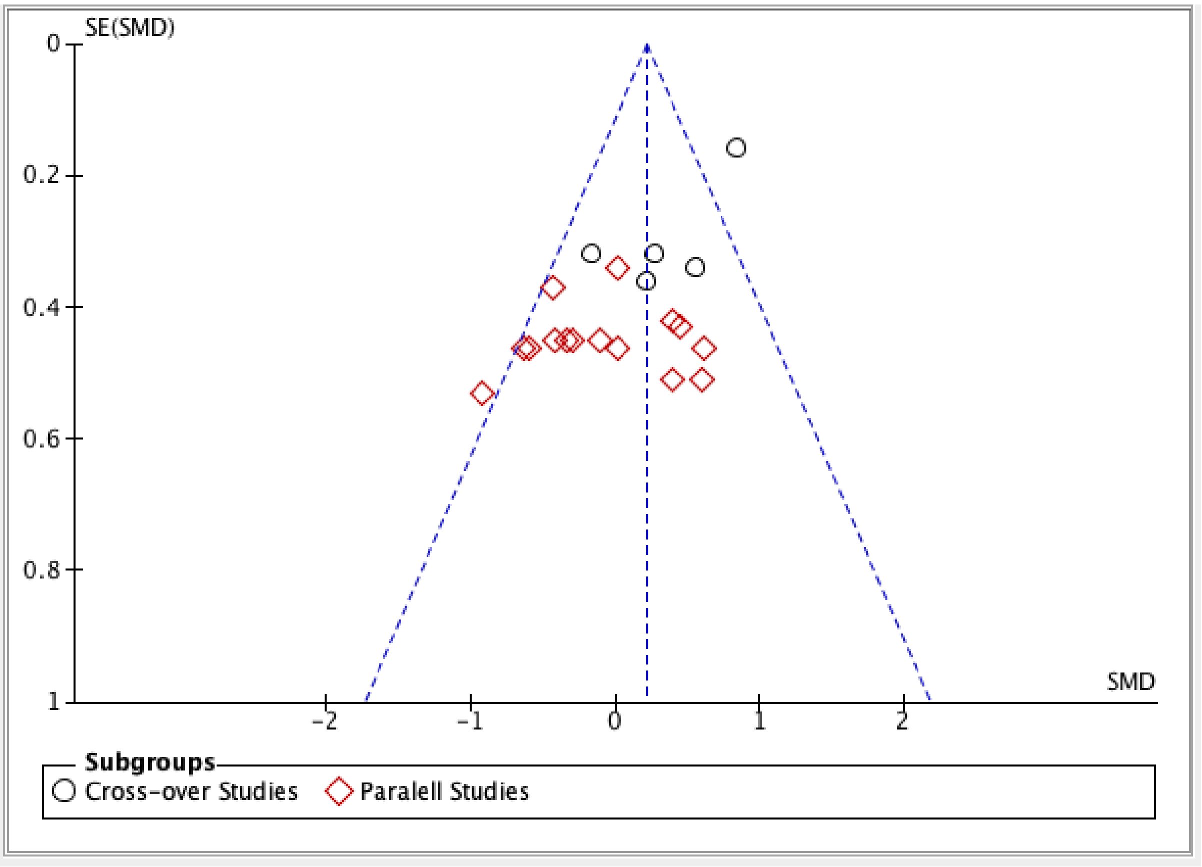

Figure S1k. CK 48 hours

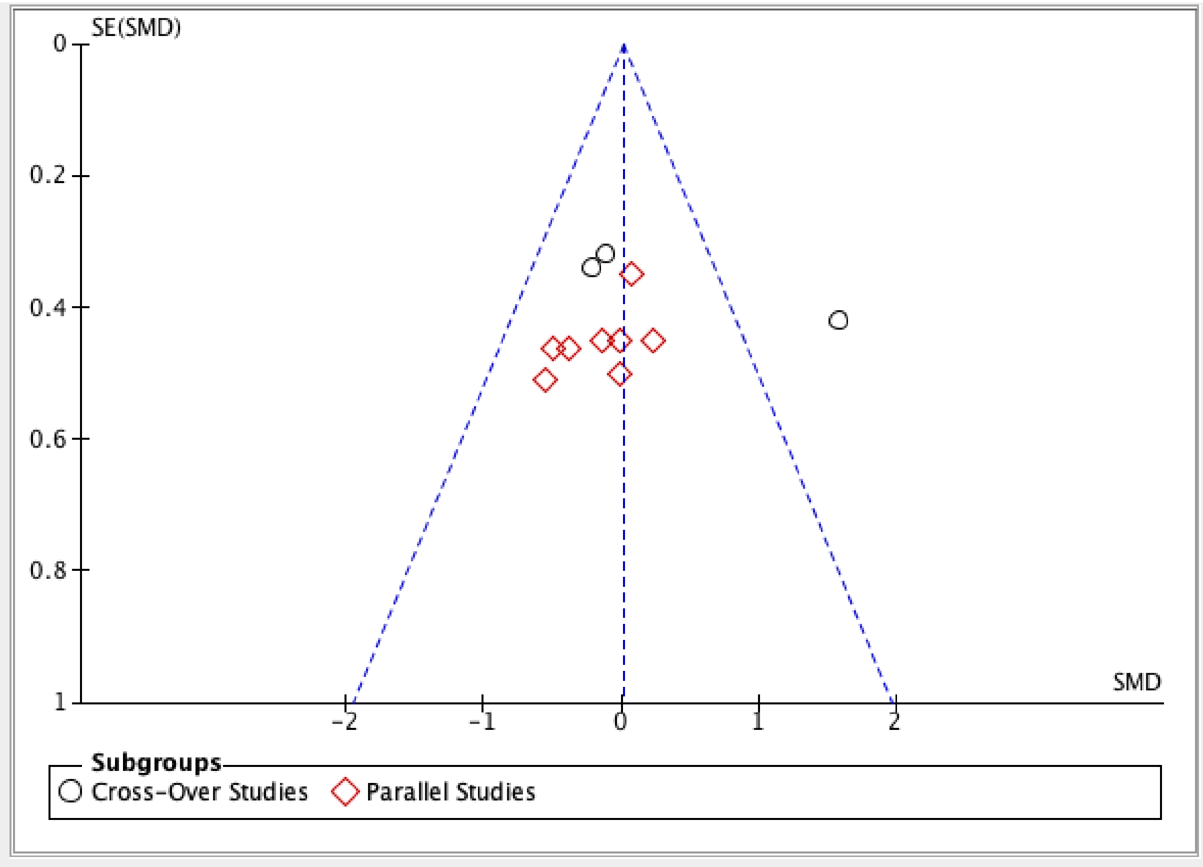

Figure S1l. CRP Post

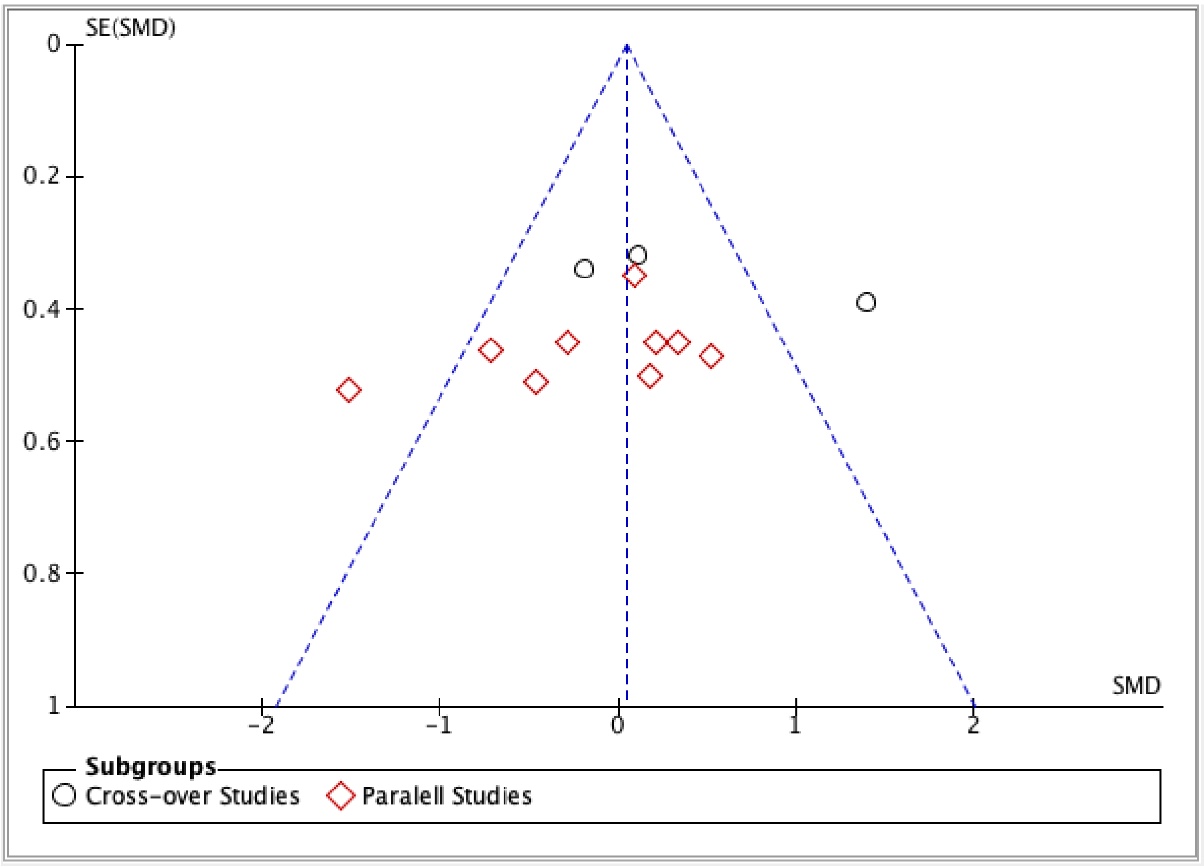

Figure S1m. CRP 24 hours

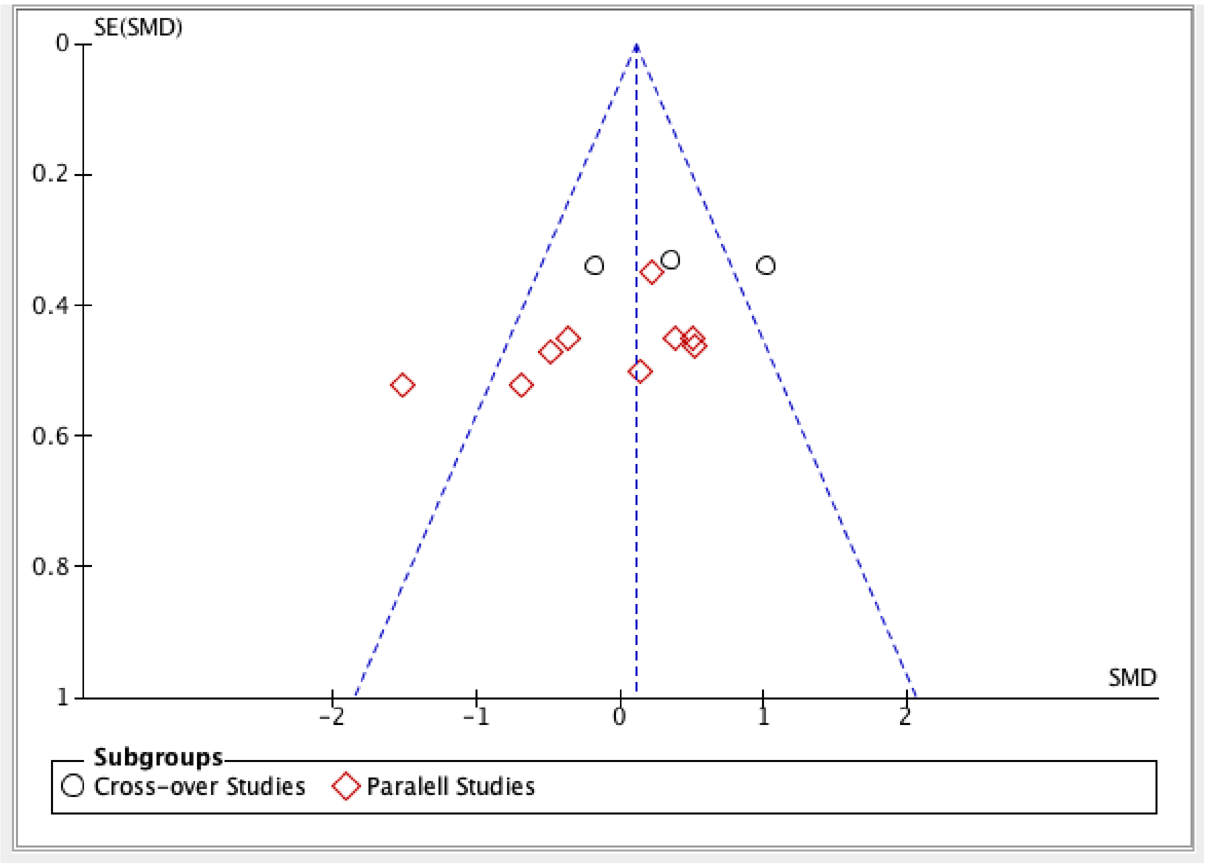

Figure S1n. CRP 48 hours
