# Supplementary material for: Effect of Polyphenol-Rich Foods, Juices, and Concentrates on Recovery from Exercise Induced Muscle Damage: A Systematic Review and Meta-Analysis
Source: Nutrients. 2021 Aug 27;13(9):2988. doi: 10.3390/nu13092988 (PMC8465563; doi:10.3390/nu13092988)
Supplement: Supplementary file 1 [file nutrients-13-02988-s001.zip › Supplementary File 3 GRADE_Edited.pdf]

Supplementary File 3 Quality assessment

Outcomes	No of studies (Design)	Limitations	Inconsistency	Indirectness	Imprecision	Publication bias	SMD [95% CI]	No of participants	Quality of the evidence (GRADE)
MIVC immediately post	14 (4 crossover; 10 parallel)	Serious limitations (3 high risk of bias, 11 some concerns)[a]	No serious inconsistency	No serious indirectness	Serious imprecision (less than 400 participants)[b]	Undetected	0.23 [0.04, 0.42]	370 (203 polyphenol; 167 placebo)	⊕⊕◯◯ Low
MIVC 24 hrs post	19 (7 crossover; 12 parallel)	Serious limitations (7 high risk of bias, 12 some concerns) [a]	Serious inconsistency[d]	No serious indirectness	No serious imprecision	Undetected	0.39 [0.15, 0.62]	466 (251 polyphenol; 215 placebo)	⊕⊕◯◯ Low
MIVC 48 hrs post	19 (7 crossover; 12 parallel)	Serious limitations (7 high risk of bias, 12 some concerns) [a]	No serious inconsistency	No serious indirectness	No serious imprecision	Undetected	0.48 [0.28, 0.67]	466 (251 polyphenol; 215 placebo)	⊕⊕⊕◯ Moderate
MIVC 72 hrs post	13 (4 crossover; 9 parallel)	Serious limitations (4 high risk of bias, 9 some concerns) [a]	No serious inconsistency	No serious indirectness	Serious imprecision (less than 400 participants) [b]	Undetected	0.29 [0.11, 0.46]	338 (187 polyphenol; 151 placebo)	⊕⊕◯◯ Low
MIVC 96 hrs post	5 (2 crossover; 3 parallel)	Serious limitations (2 high risk of bias, 3 some concerns) [a]	Serious inconsistency[d]	No serious indirectness	Serious imprecision (less than 400 participants) [b]	Undetected	0.50 [0.16, 0.83]	170 (98 polyphenol; 72 placebo)	⊕◯◯◯ Very Low
DOMS immediately post	15 (4 crossover; 11 parallel)	Serious limitations (3 high risk of bias, 12 some concerns) [a]	Serious inconsistency[d]	No serious indirectness	No serious imprecision	Undetected	-0.23 [-0.53, 0.07]	414 (226 polyphenol; 188 placebo)	⊕⊕◯◯ Low
DOMS 24 hrs post	24 (10 crossover; 14 parallel)	Serious limitations (9 high risk of bias, 15 some concerns) [a]	Serious inconsistency[d]	No serious indirectness	No serious imprecision	Undetected	-0.29 [-0.47, -0.11]	567 (302 polyphenol; 265 placebo)	⊕⊕◯◯ Low
DOMS 48 hrs post	24 (10 crossover; 14 parallel)	Serious limitations (9 high risk of bias, 15 some concerns) [a]	Serious inconsistency[d]	No serious indirectness	No serious imprecision	Undetected	-0.28 [-0.46, -0.09]	567 (302 polyphenol; 265 placebo)	⊕⊕◯◯ Low
DOMS 72 hrs post	16 (6 crossover; 10 parallel)	Serious limitations (5 high risk of bias, 11 some concerns) [a]	Serious inconsistency[d]	No serious indirectness	Serious imprecision (less than 400	Undetected	-0.46 [-0.69, -0.24]	396 (217 polyphenol; 179 placebo)	⊕◯◯◯ Very Low

					participants and CI)[c]			
DOMS 96 hrs post	5 (2 crossover; 3 parallel)	Serious limitations (2 high risk of bias, 3 some concerns)[a]	No serious inconsistency	No serious indirectness	Serious imprecision (less than 400 participants)[b]	Undetected	-0.10 [-0.30, 0.10]	158 (93 polyphenol; 65 placebo)	⊕⊕◯◯ Low
CMJ immediately post	8 (2 crossover; 6 parallel)	Serious limitations (1 high risk of bias, 4 some concerns)[a]	No serious inconsistency	No serious indirectness	Serious imprecision (less than 400 participants)[b]	Undetected	0.27 [0.01, 0.53]	190 (100 polyphenol; 90 placebo)	⊕⊕◯◯ Low
CMJ 24 hrs post	10 (3 crossover; 7 parallel)	Serious limitations (2 high risk of bias, 8 some concerns)[a]	Serious inconsistency[d]	No serious indirectness	Serious imprecision (less than 400 participants)[b]	Undetected	0.47 [0.11, 0.83]	216 (118 polyphenol; 108 placebo)	⊕◯◯◯ Very Low
CMJ 48 hrs post	11 (4 crossover; 7 parallel)	Serious limitations (3 high risk of bias, 8 some concerns)[a]	Serious inconsistency[d]	No serious indirectness	Serious imprecision (less than 400 participants)[b]	Undetected	0.58 [0.24, 0.91]	248 (129 polyphenol; 119 placebo)	⊕◯◯◯ Very Low
CMJ 72 hrs post	7 (2 crossover; 5 parallel)	Serious limitations (1 high risk of bias, 6 some concerns)[a]	Serious inconsistency[d]	No serious indirectness	Serious imprecision (less than 400 participants)[b]	Undetected	0.57 [0.03, 1.10]	152 (81 polyphenol; 71 placebo)	⊕◯◯◯ Very Low

[a] Serious risk of bias for MIVC, DOMS and CMJ at every post-exercise time point.

[b] Serious imprecision for less than 400 participants

[c] Serious imprecision for less than 400 participants and 95% CI overlap with a small effect size of 0.2 for beneficial and detrimental effects

[d] Serious inconsistency when heterogeneity I^2 was greater than 40%
