# Supplementary material for: Effect of Polyphenol-Rich Foods, Juices, and Concentrates on Recovery from Exercise Induced Muscle Damage: A Systematic Review and Meta-Analysis
Source: Nutrients. 2021 Aug 27;13(9):2988. doi: 10.3390/nu13092988 (PMC8465563; doi:10.3390/nu13092988)
Supplement: Supplementary file 1 [file nutrients-13-02988-s001.zip › Supplementary File 4 Sensitivity Analysis_Edited.pdf]

(A)

Study or Subgroup	Std. Mean Difference	SE	Polyphenol Total	Placebo Total	Weight	Std. Mean Difference IV, Random, 95% CI
**37.1.1 Cross-Over Studies**						
Bowtell 2011	0.44	0.35	9	9	9.2%	0.44 [−0.25, 1.13]
Daab et al. 2020	0.7	0.31	13	13	11.7%	0.70 [0.09, 1.31]
Morgan et al. 2018	0.4	0.33	10	10	10.3%	0.40 [−0.25, 1.05]
Trombold 2011 (1)	0.22	0.46	17	17	5.3%	0.22 [−0.68, 1.12]
**Subtotal (95% CI)**			**49**	**49**	**36.6%**	**0.48 [0.14, 0.82]**
Heterogeneity: Tau² = 0.00; Chi² = 0.89, df = 3 (P = 0.83); I² = 0%						
Test for overall effect: Z = 2.74 (P = 0.006)						
**37.1.2 Parallel Studies**						
Brown 2018	−0.09	0.45	10	10	5.6%	−0.09 [−0.97, 0.79]
Clifford 2016a	0.12	0.45	10	10	5.6%	0.12 [−0.76, 1.00]
Clifford 2016b (2)	0.09	0.39	20	10	7.4%	0.09 [−0.67, 0.85]
Clifford 2017a	0.32	0.34	17	17	9.7%	0.32 [−0.35, 0.99]
Clifford 2017b	−0.19	0.45	10	10	5.6%	−0.19 [−1.07, 0.69]
Lamb et al. 2019 (3)	0.34	0.37	22	11	8.2%	0.34 [−0.39, 1.07]
Machin et al. 2014 (4)	−0.03	0.23	30	15	21.3%	−0.03 [−0.48, 0.42]
**Subtotal (95% CI)**			**119**	**83**	**63.4%**	**0.08 [−0.18, 0.34]**
Heterogeneity: Tau² = 0.00; Chi² = 1.73, df = 6 (P = 0.94); I² = 0%						
Test for overall effect: Z = 0.60 (P = 0.55)						
**Total (95% CI)**			**168**	**132**	**100.0%**	**0.23 [0.02, 0.43]**
Heterogeneity: Tau² = 0.00; Chi² = 5.93, df = 10 (P = 0.82); I² = 0%						
Test for overall effect: Z = 2.13 (P = 0.03)						
Test for subgroup differences: Chi² = 3.30, df = 1 (P = 0.07), I² = 69.7%						

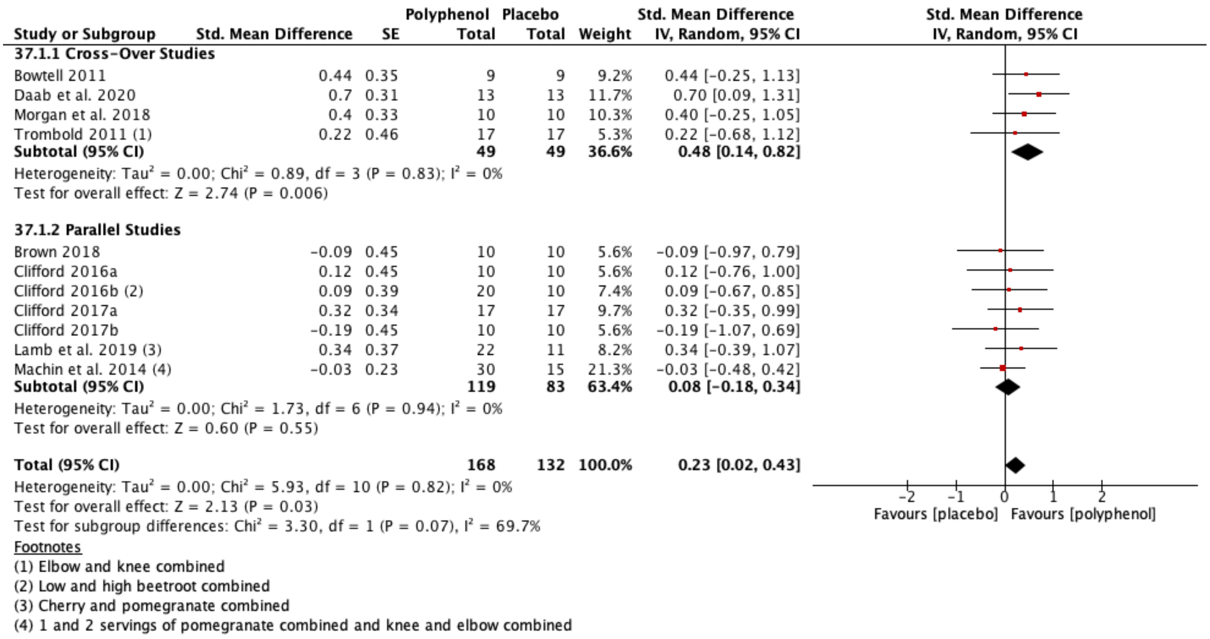

Footnotes
(1) Elbow and knee combined
(2) Low and high beetroot combined
(3) Cherry and pomegranate combined
(4) 1 and 2 servings of pomegranate combined and knee and elbow combined

(B)

Study or Subgroup	Std. Mean Difference	SE	Polyphenol Total	Placebo Total	Weight	Std. Mean Difference IV, Random, 95% CI
**37.2.1 Cross-over Studies**						
Bowtell 2011	0.58	0.36	9	9	7.8%	0.58 [−0.13, 1.29]
Daab et al. 2020	1.55	0.41	13	13	6.3%	1.55 [0.75, 2.35]
McLeay 2012 (1)	0.09	0.32	10	10	9.4%	0.09 [−0.54, 0.72]
Morgan et al. 2018	0.05	0.32	10	10	9.4%	0.05 [−0.58, 0.68]
Trombold 2011 (2)	0.33	0.25	17	17	13.4%	0.33 [−0.16, 0.82]
**Subtotal (95% CI)**			**59**	**59**	**46.3%**	**0.47 [0.01, 0.93]**
Heterogeneity: Tau² = 0.17; Chi² = 10.32, df = 4 (P = 0.04); I² = 61%						
Test for overall effect: Z = 2.01 (P = 0.04)						
**37.2.2 Paralell Studies**						
Brown 2018	0.09	0.45	10	10	5.4%	0.09 [−0.79, 0.97]
Clifford 2016a	0.09	0.45	10	10	5.4%	0.09 [−0.79, 0.97]
Clifford 2016b (3)	0.03	0.39	20	10	6.9%	0.03 [−0.73, 0.79]
Clifford 2017a	0.44	0.35	17	17	8.2%	0.44 [−0.25, 1.13]
Clifford 2017b	−0.11	0.45	10	10	5.4%	−0.11 [−0.99, 0.77]
Lamb et al. 2019 (4)	−0.12	0.37	22	11	7.5%	−0.12 [−0.85, 0.61]
Machin et al. 2014 (5)	0.28	0.23	30	15	14.8%	0.28 [−0.17, 0.73]
**Subtotal (95% CI)**			**119**	**83**	**53.7%**	**0.15 [−0.11, 0.42]**
Heterogeneity: Tau² = 0.00; Chi² = 2.00, df = 6 (P = 0.92); I² = 0%						
Test for overall effect: Z = 1.15 (P = 0.25)						
**Total (95% CI)**			**178**	**142**	**100.0%**	**0.28 [0.05, 0.50]**
Heterogeneity: Tau² = 0.03; Chi² = 14.09, df = 11 (P = 0.23); I² = 22%						
Test for overall effect: Z = 2.44 (P = 0.01)						
Test for subgroup differences: Chi² = 1.38, df = 1 (P = 0.24), I² = 27.7%						

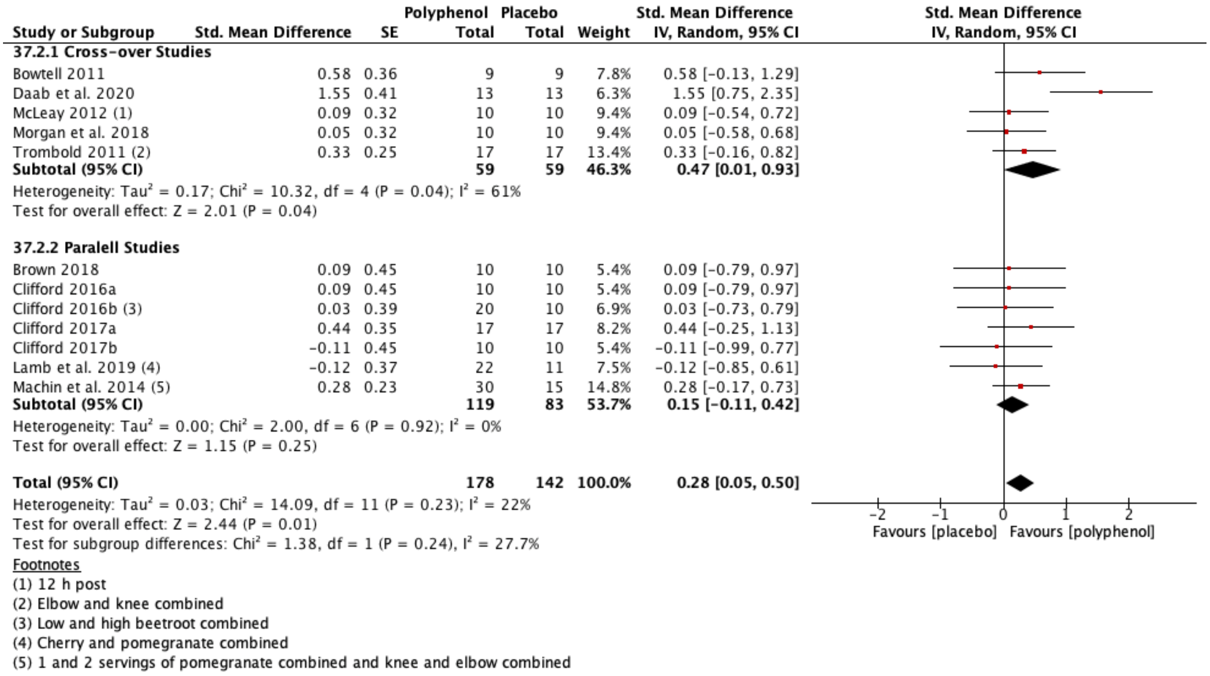

Footnotes
(1) 12 h post
(2) Elbow and knee combined
(3) Low and high beetroot combined
(4) Cherry and pomegranate combined
(5) 1 and 2 servings of pomegranate combined and knee and elbow combined

(C)

Study or Subgroup	Std. Mean Difference	SE	Polyphenol Total	Placebo Total	Weight	Std. Mean Difference IV, Random, 95% CI
**37.3.1 Cross-over Studies**						
Bowtell 2011	0.42	0.35	9	9	7.6%	0.42 [−0.27, 1.11]
Daab et al. 2020	1.06	0.35	13	13	7.6%	1.06 [0.37, 1.75]
McLeay 2012 (1)	0.58	0.34	10	10	8.1%	0.58 [−0.09, 1.25]
Morgan et al. 2018	0.4	0.33	10	10	8.6%	0.40 [−0.25, 1.05]
Trombold 2011 (2)	0.56	0.26	17	17	13.8%	0.56 [0.05, 1.07]
**Subtotal (95% CI)**			**59**	**59**	**45.8%**	**0.59 [0.31, 0.87]**
Heterogeneity: $Tau^2 = 0.00$; $Chi^2 = 2.38$, df = 4 (P = 0.67); $I^2 = 0\%$						
Test for overall effect: Z = 4.15 (P < 0.0001)						
**37.3.2 Paralell Studies**						
Brown 2018	0.24	0.45	10	10	4.6%	0.24 [−0.64, 1.12]
Clifford 2016a	0.12	0.45	10	10	4.6%	0.12 [−0.76, 1.00]
Clifford 2016b (3)	0.16	0.39	20	10	6.1%	0.16 [−0.60, 0.92]
Clifford 2017a	0.23	0.34	17	17	8.1%	0.23 [−0.44, 0.90]
Clifford 2017b	−0.07	0.45	10	10	4.6%	−0.07 [−0.95, 0.81]
Lamb et al. 2019 (4)	−0.06	0.37	22	11	6.8%	−0.06 [−0.79, 0.67]
Machin et al. 2014 (5)	0.11	0.22	30	15	19.3%	0.11 [−0.32, 0.54]
**Subtotal (95% CI)**			**119**	**83**	**54.2%**	**0.11 [−0.15, 0.37]**
Heterogeneity: $Tau^2 = 0.00$; $Chi^2 = 0.60$, df = 6 (P = 1.00); $I^2 = 0\%$						
Test for overall effect: Z = 0.83 (P = 0.41)						
**Total (95% CI)**			**178**	**142**	**100.0%**	**0.33 [0.14, 0.52]**
Heterogeneity: $Tau^2 = 0.00$; $Chi^2 = 9.22$, df = 11 (P = 0.60); $I^2 = 0\%$						
Test for overall effect: Z = 3.42 (P = 0.0006)						
Test for subgroup differences: $Chi^2 = 6.24$, df = 1 (P = 0.01), $I^2 = 84.0\%$						

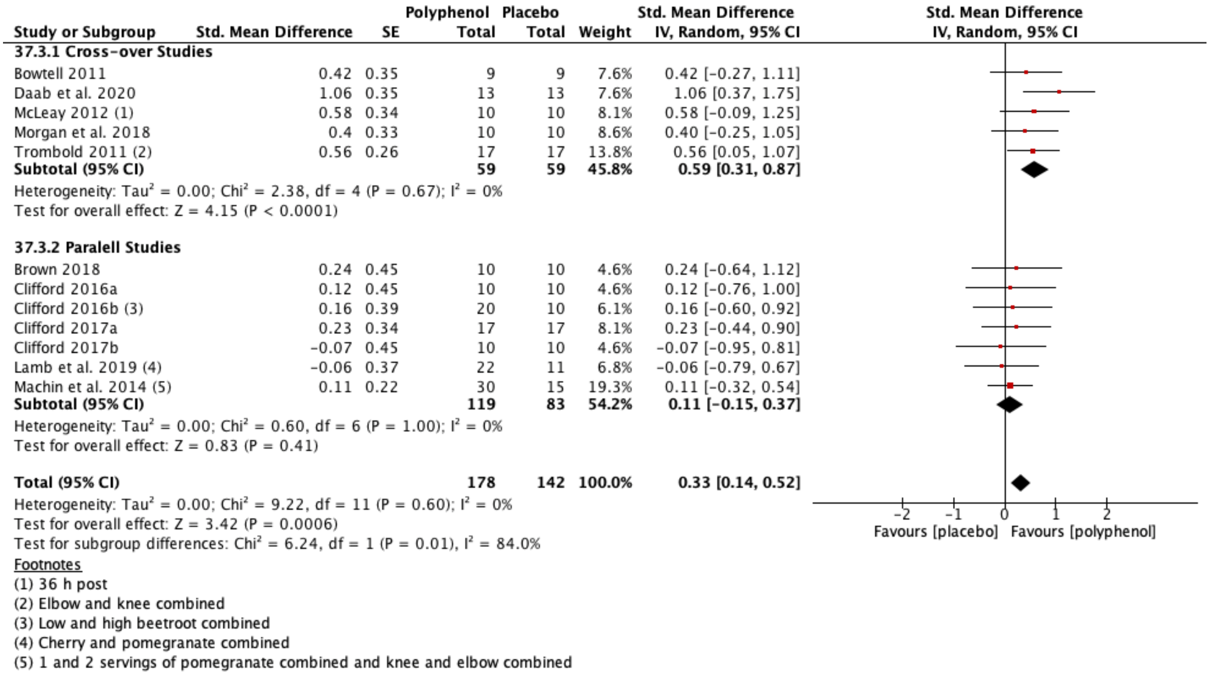

Footnotes
(1) 36 h post
(2) Elbow and knee combined
(3) Low and high beetroot combined
(4) Cherry and pomegranate combined
(5) 1 and 2 servings of pomegranate combined and knee and elbow combined

(D)

Study or Subgroup	Std. Mean Difference	SE	Polyphenol Total	Placebo Total	Weight	Std. Mean Difference IV, Random, 95% CI
**37.4.1 Cross-Over Studies**						
Daab et al. 2020	0.44	0.29	13	13	11.3%	0.44 [−0.13, 1.01]
McLeay 2012 (1)	0.1	0.32	10	10	9.2%	0.10 [−0.53, 0.73]
Trombold 2011 (2)	0.22	0.17	17	17	32.8%	0.22 [−0.11, 0.55]
**Subtotal (95% CI)**			**40**	**40**	**53.3%**	**0.25 [−0.02, 0.51]**
Heterogeneity: $Tau^2 = 0.00$; $Chi^2 = 0.68$, df = 2 (P = 0.71); $I^2 = 0\%$						
Test for overall effect: Z = 1.84 (P = 0.07)						
**37.4.2 Paralell Studies**						
Brown 2018	0.01	0.45	10	10	4.7%	0.01 [−0.87, 0.89]
Clifford 2016a	0.3	0.45	10	10	4.7%	0.30 [−0.58, 1.18]
Clifford 2016b (3)	0.07	0.39	20	10	6.2%	0.07 [−0.69, 0.83]
Clifford 2017b	0.13	0.45	10	10	4.7%	0.13 [−0.75, 1.01]
Lamb et al. 2019 (4)	−0.05	0.37	22	11	6.9%	−0.05 [−0.78, 0.68]
Machin et al. 2014 (5)	0.29	0.22	30	15	19.6%	0.29 [−0.14, 0.72]
**Subtotal (95% CI)**			**102**	**66**	**46.7%**	**0.17 [−0.11, 0.45]**
Heterogeneity: $Tau^2 = 0.00$; $Chi^2 = 0.93$, df = 5 (P = 0.97); $I^2 = 0\%$						
Test for overall effect: Z = 1.18 (P = 0.24)						
**Total (95% CI)**			**142**	**106**	**100.0%**	**0.21 [0.02, 0.40]**
Heterogeneity: $Tau^2 = 0.00$; $Chi^2 = 1.77$, df = 8 (P = 0.99); $I^2 = 0\%$						
Test for overall effect: Z = 2.15 (P = 0.03)						
Test for subgroup differences: $Chi^2 = 0.16$, df = 1 (P = 0.69), $I^2 = 0\%$						

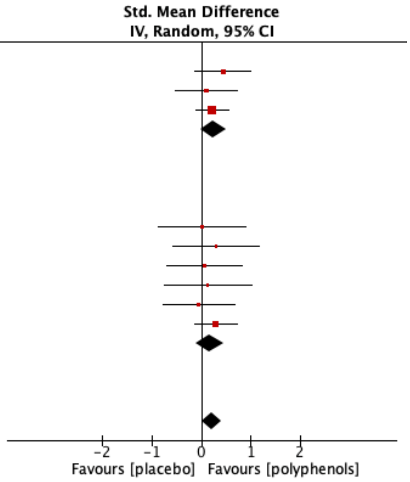

Footnotes
(1) 60 h post
(2) Elbow and knee combined
(3) Low and high beetroot combined
(4) Cherry and pomegranate combined
(5) 1 and 2 servings of pomegranate combined and knee and elbow combined

(E)

Study or Subgroup	Std. Mean Difference	SE	Polyphenol Total	Placebo Total	Weight	Std. Mean Difference IV, Random, 95% CI
**37.5.1 Cross-over studies**						
Trombold 2011 (1)	0.45	0.18	17	17	54.1%	0.45 [0.10, 0.80]
**Subtotal (95% CI)**			**17**	**17**	**54.1%**	**0.45 [0.10, 0.80]**
Heterogeneity: Not applicable						
Test for overall effect: Z = 2.50 (P = 0.01)						
**37.5.2 Paralell Studies**						
Lamb et al. 2019 (2)	0.08	0.37	22	11	12.8%	0.08 [−0.65, 0.81]
Machin et al. 2014 (3)	0.34	0.23	30	15	33.1%	0.34 [−0.11, 0.79]
**Subtotal (95% CI)**			**52**	**26**	**45.9%**	**0.27 [−0.12, 0.65]**
Heterogeneity: $Tau^2 = 0.00$; $Chi^2 = 0.36$, df = 1 (P = 0.55); $I^2 = 0\%$						
Test for overall effect: Z = 1.37 (P = 0.17)						
**Total (95% CI)**			**69**	**43**	**100.0%**	**0.37 [0.11, 0.63]**
Heterogeneity: $Tau^2 = 0.00$; $Chi^2 = 0.83$, df = 2 (P = 0.66); $I^2 = 0\%$						
Test for overall effect: Z = 2.77 (P = 0.006)						
Test for subgroup differences: $Chi^2 = 0.47$, df = 1 (P = 0.49), $I^2 = 0\%$						

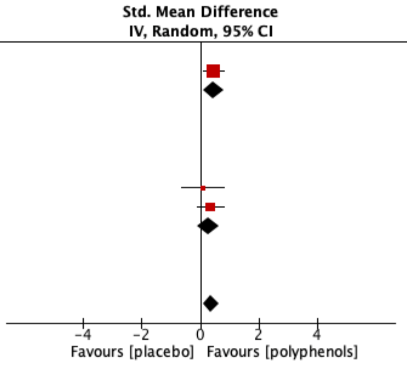

Footnotes
(1) Elbow and knee combined
(2) Cherry and pomegranate combined
(3) 1 and 2 servings of pomegranate combined and knee and elbow combined

Supplementary Figure 4a. Sensitivity analysis: Effect of polyphenol-rich foods, juices and concentrates on recovery of maximal isometric voluntary contraction (A) immediately post-exercise; (B) 24 hours; (C) 48 hours; (D) 72 hours; (E) 96 hours after excluding 10 studies classified as high risk of bias.

(A)

Study or Subgroup	Std. Mean Difference	SE	Polyphenol Total	Placebo Total	Weight	Std. Mean Difference IV, Random, 95% CI
**38.1.1 Cross-Over Studies**						
Bowtell 2011 (1)	−0.19	0.32	10	10	9.2%	−0.19 [−0.82, 0.44]
Daab et al. 2020	−1.3	0.38	13	13	7.9%	−1.30 [−2.04, −0.56]
Morgan et al. 2018	0.16	0.32	10	10	9.2%	0.16 [−0.47, 0.79]
Trombold 2011 (2)	−0.18	0.2806	17	17	10.2%	−0.18 [−0.73, 0.37]
**Subtotal (95% CI)**			**50**	**50**	**36.5%**	**−0.35 [−0.90, 0.21]**
Heterogeneity: Tau² = 0.22; Chi² = 9.29, df = 3 (P = 0.03); I² = 68%						
Test for overall effect: Z = 1.22 (P = 0.22)						
**38.1.2 Parallel Studies**						
Brown 2018 (3)	−0.91	0.32	10	10	9.2%	−0.91 [−1.54, −0.28]
Clifford 2016a (4)	−0.01	0.45	10	10	6.6%	−0.01 [−0.89, 0.87]
Clifford 2016b (5)	−0.21	0.39	20	10	7.7%	−0.21 [−0.97, 0.55]
Clifford 2017a	−0.06	0.34	17	17	8.8%	−0.06 [−0.73, 0.61]
Clifford 2017b (6)	−0.48	0.45	10	10	6.6%	−0.48 [−1.36, 0.40]
Lamb et al. 2019 (7)	0.7	0.38	24	11	7.9%	0.70 [−0.04, 1.44]
Lynn 2018	0.47	0.47	9	10	6.3%	0.47 [−0.45, 1.39]
Machin et al. 2014 (8)	0.13	0.27	30	15	10.5%	0.13 [−0.40, 0.66]
**Subtotal (95% CI)**			**130**	**93**	**63.5%**	**−0.06 [−0.43, 0.30]**
Heterogeneity: Tau² = 0.13; Chi² = 13.84, df = 7 (P = 0.05); I² = 49%						
Test for overall effect: Z = 0.34 (P = 0.74)						
**Total (95% CI)**			**180**	**143**	**100.0%**	**−0.16 [−0.46, 0.13]**
Heterogeneity: Tau² = 0.15; Chi² = 24.30, df = 11 (P = 0.01); I² = 55%						
Test for overall effect: Z = 1.07 (P = 0.28)						
Test for subgroup differences: Chi² = 0.70, df = 1 (P = 0.40), I² = 0%						

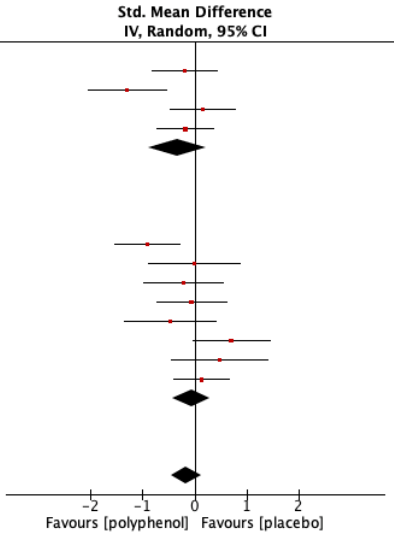

Footnotes
(1) PPT
(2) Elbow and knee combined
(3) PPT and VAS combined
(4) PPT
(5) PPT low and high beetroot combined
(6) PPT
(7) Cherry and pomegranate combined
(8) 1 and 2 servings of pomegranate combined and knee and elbow combined

(B)

Study or Subgroup	Std. Mean Difference	SE	Polyphenol Total	Placebo Total	Weight	Std. Mean Difference IV, Random, 95% CI
**38.2.1 Cross-over Studies**						
Abbot et al. 2019 (1)	0.11	0.34	10	10	6.7%	0.11 [−0.56, 0.78]
Bowtell 2011 (2)	−0.28	0.34	10	10	6.7%	−0.28 [−0.95, 0.39]
Daab et al. 2020	−1.46	0.4	13	13	5.7%	−1.46 [−2.24, −0.68]
Kupusaravic et al. 2019	−0.45	0.33	10	10	6.8%	−0.45 [−1.10, 0.20]
McLeay 2012 (3)	−0.17	0.32	10	10	7.0%	−0.17 [−0.80, 0.46]
Morgan et al. 2018	0.15	0.32	10	10	7.0%	0.15 [−0.48, 0.78]
Trombold 2011 (4)	−0.25	0.18	17	17	9.5%	−0.25 [−0.60, 0.10]
**Subtotal (95% CI)**			**80**	**80**	**49.4%**	**−0.30 [−0.62, 0.03]**
Heterogeneity: Tau² = 0.09; Chi² = 12.23, df = 6 (P = 0.06); I² = 51%						
Test for overall effect: Z = 1.80 (P = 0.07)						
**38.2.2 Parallel studies**						
Brown 2018 (5)	−1.09	0.24	10	10	8.4%	−1.09 [−1.56, −0.62]
Clifford 2016a (6)	−0.09	0.45	10	10	5.1%	−0.09 [−0.97, 0.79]
Clifford 2016b (7)	−1.08	0.42	20	10	5.5%	−1.08 [−1.90, −0.26]
Clifford 2017a	−0.04	0.34	17	17	6.7%	−0.04 [−0.71, 0.63]
Clifford 2017b (8)	−0.47	0.45	10	10	5.1%	−0.47 [−1.35, 0.41]
Lamb et al. 2019 (9)	0.61	0.37	24	11	6.2%	0.61 [−0.12, 1.34]
Lynn 2018	0.02	0.46	9	10	5.0%	0.02 [−0.88, 0.92]
Machin et al. 2014 (10)	0.09	0.22	30	15	8.8%	0.09 [−0.34, 0.52]
**Subtotal (95% CI)**			**130**	**93**	**50.6%**	**−0.26 [−0.72, 0.19]**
Heterogeneity: Tau² = 0.29; Chi² = 24.90, df = 7 (P = 0.0008); I² = 72%						
Test for overall effect: Z = 1.14 (P = 0.26)						
**Total (95% CI)**			**210**	**173**	**100.0%**	**−0.29 [−0.55, −0.02]**
Heterogeneity: Tau² = 0.16; Chi² = 37.15, df = 14 (P = 0.0007); I² = 62%						
Test for overall effect: Z = 2.10 (P = 0.04)						
Test for subgroup differences: Chi² = 0.01, df = 1 (P = 0.90), I² = 0%						

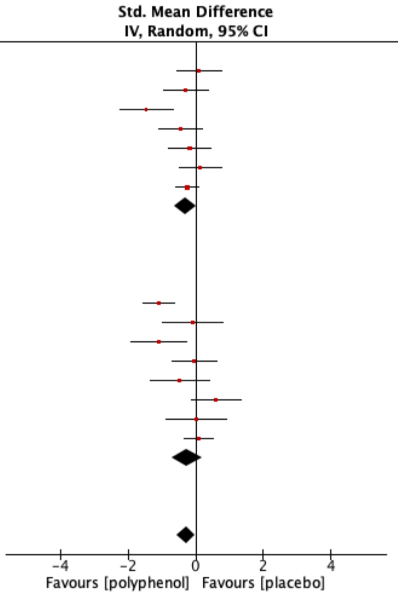

Footnotes
(1) 12 h post
(2) PPT
(3) 12 h post
(4) Elbow and knee combined
(5) PPT and VAS combined
(6) PPT
(7) PPT low and high beetroot combined
(8) PPT
(9) Cherry and pomegranate combined
(10) 1 and 2 servings of pomegranate combined and knee and elbow combined

(C)

Study or Subgroup	Std. Mean Difference	SE	Polyphenol Total	Placebo Total	Weight	Std. Mean Difference IV, Random, 95% CI
**38.3.1 Cross-over studies**						
Abbot et al. 2019 (1)	0.15	0.44	10	10	4.9%	0.15 [−0.71, 1.01]
Bowtell 2011 (2)	−0.27	0.45	10	10	4.7%	−0.27 [−1.15, 0.61]
Daab et al. 2020	−0.55	0.3	13	13	7.7%	−0.55 [−1.14, 0.04]
Kupusaravic et al. 2019	−0.25	0.32	10	10	7.2%	−0.25 [−0.88, 0.38]
McLeay 2012 (3)	0.21	0.32	10	10	7.2%	0.21 [−0.42, 0.84]
Morgan et al. 2018	−0.03	0.32	10	10	7.2%	−0.03 [−0.66, 0.60]
Trombold 2011 (4)	−0.39	0.18	17	17	11.3%	−0.39 [−0.74, −0.04]
**Subtotal (95% CI)**			**80**	**80**	**50.3%**	**−0.24 [−0.46, −0.02]**
Heterogeneity: $Tau^2 = 0.00$; $Chi^2 = 4.96$, df = 6 (P = 0.55); $I^2 = 0\%$						
Test for overall effect: Z = 2.15 (P = 0.03)						
**38.3.2 Paralell studies**						
Brown 2018 (5)	−1.09	0.29	10	10	8.0%	−1.09 [−1.66, −0.52]
Clifford 2016a (6)	−0.29	0.45	10	10	4.7%	−0.29 [−1.17, 0.59]
Clifford 2016b (7)	−0.88	0.41	20	10	5.4%	−0.88 [−1.68, −0.08]
Clifford 2017a	−0.53	0.35	17	17	6.5%	−0.53 [−1.22, 0.16]
Clifford 2017b (8)	−0.48	0.46	10	10	4.6%	−0.48 [−1.38, 0.42]
Lamb et al. 2019 (9)	0.66	0.38	24	11	5.9%	0.66 [−0.08, 1.40]
Lynn 2018	0.49	0.47	9	10	4.5%	0.49 [−0.43, 1.41]
Machin et al. 2014 (10)	0	0.22	30	15	10.0%	0.00 [−0.43, 0.43]
**Subtotal (95% CI)**			**130**	**93**	**49.7%**	**−0.28 [−0.71, 0.15]**
Heterogeneity: $Tau^2 = 0.25$; $Chi^2 = 21.06$, df = 7 (P = 0.004); $I^2 = 67\%$						
Test for overall effect: Z = 1.27 (P = 0.20)						
**Total (95% CI)**			**210**	**173**	**100.0%**	**−0.24 [−0.47, −0.01]**
Heterogeneity: $Tau^2 = 0.09$; $Chi^2 = 26.11$, df = 14 (P = 0.03); $I^2 = 46\%$						
Test for overall effect: Z = 2.04 (P = 0.04)						
Test for subgroup differences: $Chi^2 = 0.03$, df = 1 (P = 0.86), $I^2 = 0\%$						

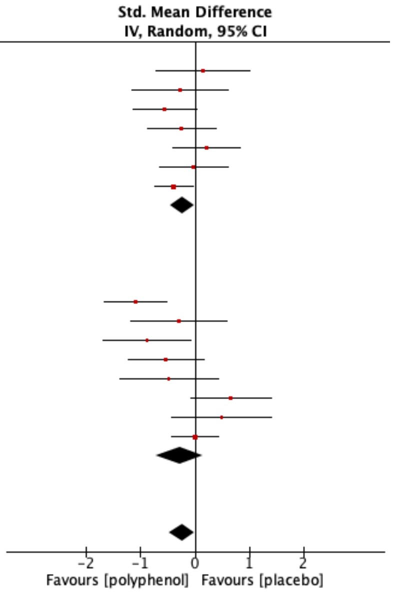

Footnotes
(1) 36 h post
(2) PPT
(3) 36 h post
(4) Elbow and knee combined
(5) PPT and VAS combined
(6) PPT
(7) PPT low and high beetroot combined
(8) PPT
(9) Cherry and pomegranate combined
(10) 1 and 2 servings of pomegranate combined and knee and elbow combined

(D)

Study or Subgroup	Std. Mean Difference	SE	Polyphenol Total	Placebo Total	Weight	Std. Mean Difference IV, Random, 95% CI
**38.4.1 Cross-over Studies**						
Abbot et al. 2019 (1)	−0.2	0.33	10	10	8.9%	−0.20 [−0.85, 0.45]
Daab et al. 2020	−1.11	0.35	13	13	8.5%	−1.11 [−1.80, −0.42]
Kupusaravic et al. 2019	−0.34	0.33	10	10	8.9%	−0.34 [−0.99, 0.31]
McLeay 2012 (2)	0.08	0.32	10	10	9.2%	0.08 [−0.55, 0.71]
Trombold 2011 (3)	−0.3	0.18	17	17	13.0%	−0.30 [−0.65, 0.05]
**Subtotal (95% CI)**			**60**	**60**	**48.5%**	**−0.35 [−0.68, −0.02]**
Heterogeneity: $Tau^2 = 0.06$; $Chi^2 = 6.79$, df = 4 (P = 0.15); $I^2 = 41\%$						
Test for overall effect: Z = 2.07 (P = 0.04)						
**38.4.2 Parallel Studies**						
Brown 2018 (4)	−1.35	0.25	10	10	11.0%	−1.35 [−1.84, −0.86]
Clifford 2016a (5)	−0.27	0.45	10	10	6.5%	−0.27 [−1.15, 0.61]
Clifford 2016b (6)	−0.68	0.4	20	10	7.4%	−0.68 [−1.46, 0.10]
Clifford 2017b (7)	−0.5	0.45	10	10	6.5%	−0.50 [−1.38, 0.38]
Lamb et al. 2019 (8)	0.08	0.36	24	11	8.2%	0.08 [−0.63, 0.79]
Machin et al. 2014 (9)	−0.15	0.22	30	15	11.9%	−0.15 [−0.58, 0.28]
**Subtotal (95% CI)**			**104**	**66**	**51.5%**	**−0.50 [−0.99, −0.00]**
Heterogeneity: $Tau^2 = 0.26$; $Chi^2 = 17.09$, df = 5 (P = 0.004); $I^2 = 71\%$						
Test for overall effect: Z = 1.97 (P = 0.05)						
**Total (95% CI)**			**164**	**126**	**100.0%**	**−0.43 [−0.72, −0.14]**
Heterogeneity: $Tau^2 = 0.14$; $Chi^2 = 25.11$, df = 10 (P = 0.005); $I^2 = 60\%$						
Test for overall effect: Z = 2.93 (P = 0.003)						
Test for subgroup differences: $Chi^2 = 0.23$, df = 1 (P = 0.63), $I^2 = 0\%$						

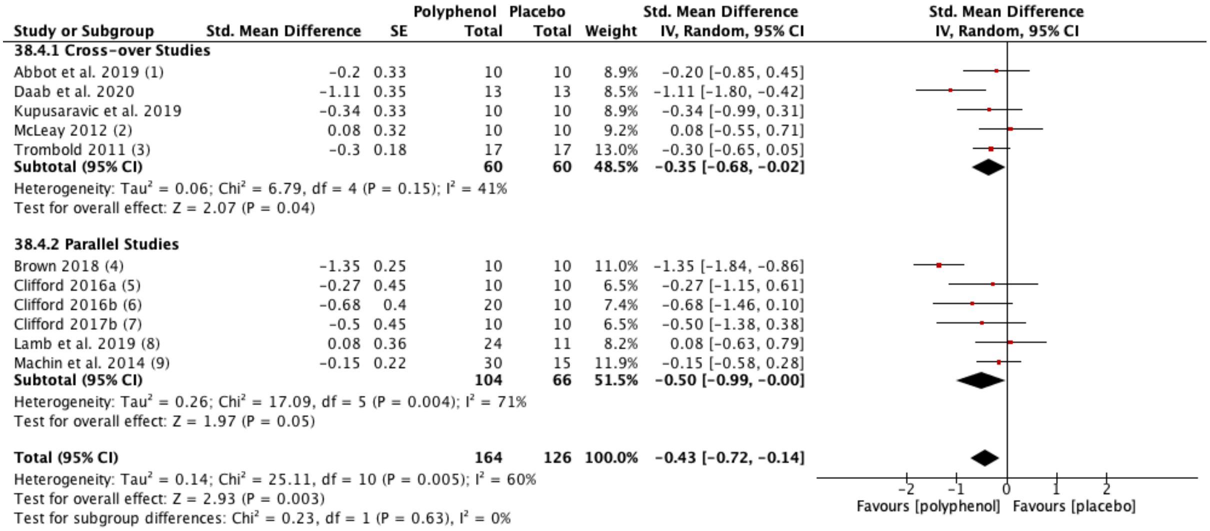

Footnotes
(1) 60 h post
(2) 60 h post
(3) Elbow and knee combined
(4) PPT and VAS combined
(5) PPT
(6) PPT low and high beetroot combined
(7) PPT
(8) Cherry and pomegranate combined
(9) 1 and 2 servings of pomegranate combined and knee and elbow combined

(E)

Study or Subgroup	Std. Mean Difference	SE	Polyphenol Total	Placebo Total	Weight	Std. Mean Difference IV, Random, 95% CI
**38.5.1 Cross-over studies**						
Trombold 2011 (1)	−0.14	0.17	17	17	54.9%	−0.14 [−0.47, 0.19]
**Subtotal (95% CI)**			**17**	**17**	**54.9%**	**−0.14 [−0.47, 0.19]**
Heterogeneity: Not applicable						
Test for overall effect: Z = 0.82 (P = 0.41)						
**38.5.2 Paralell Studies**						
Lamb et al. 2019 (2)	−0.12	0.36	24	11	12.3%	−0.12 [−0.83, 0.59]
Machin et al. 2014 (3)	0.04	0.22	30	15	32.8%	0.04 [−0.39, 0.47]
**Subtotal (95% CI)**			**54**	**26**	**45.1%**	**−0.00 [−0.37, 0.36]**
Heterogeneity: $Tau^2 = 0.00$; $Chi^2 = 0.14$, df = 1 (P = 0.70); $I^2 = 0\%$						
Test for overall effect: Z = 0.02 (P = 0.99)						
**Total (95% CI)**			**71**	**43**	**100.0%**	**−0.08 [−0.33, 0.17]**
Heterogeneity: $Tau^2 = 0.00$; $Chi^2 = 0.43$, df = 2 (P = 0.80); $I^2 = 0\%$						
Test for overall effect: Z = 0.62 (P = 0.53)						
Test for subgroup differences: $Chi^2 = 0.29$, df = 1 (P = 0.59), $I^2 = 0\%$						

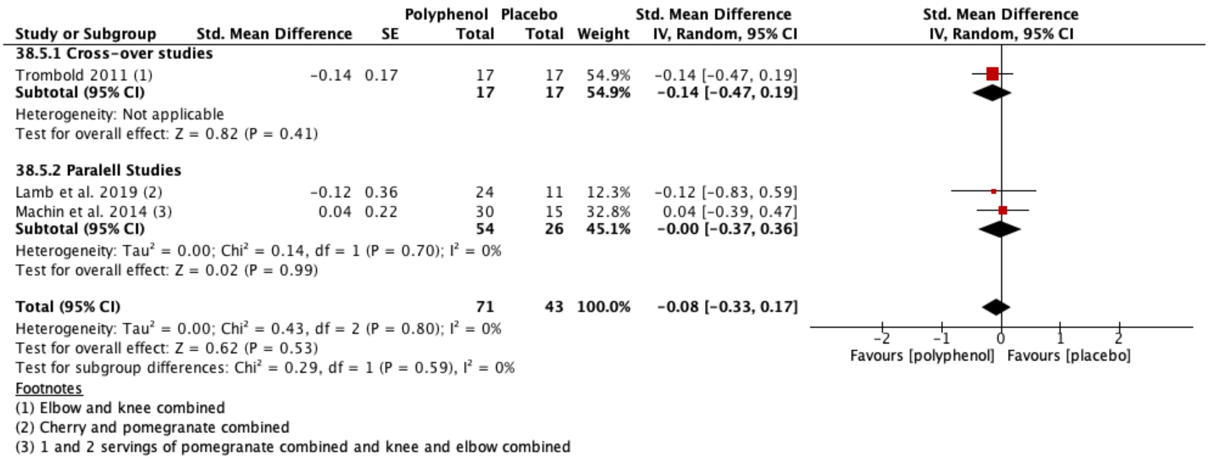

Footnotes
(1) Elbow and knee combined
(2) Cherry and pomegranate combined
(3) 1 and 2 servings of pomegranate combined and knee and elbow combined

Supplementary Figure 4b. Sensitivity analysis: Effect of polyphenol-rich foods, juices and concentrates on recovery of delayed onset muscle soreness (A) immediately post-exercise; (B) 24 hours; (C) 48 hours; (D) 72 hours; (E) 96 hours after excluding 10 studies classified as high risk of bias.

PPT: Pain pressure threshold

VAS: Visual analogue scale

(A)

Study or Subgroup	Std. Mean Difference	SE	Polyphenol Total	Placebo Total	Weight	Std. Mean Difference IV, Random, 95% CI
**39.1.1 Cross-Over Studies**						
Daab et al. 2020	0.28	0.3	13	13	22.0%	0.28 [−0.31, 0.87]
Morgan et al. 2018	0.31	0.32	10	10	19.3%	0.31 [−0.32, 0.94]
**Subtotal (95% CI)**			**23**	**23**	**41.3%**	**0.29 [−0.13, 0.72]**
Heterogeneity: Tau² = 0.00; Chi² = 0.00, df = 1 (P = 0.95); I² = 0%						
Test for overall effect: Z = 1.34 (P = 0.18)						
**39.1.2 Parallel Studies**						
Brown 2018	0.56	0.46	10	10	9.4%	0.56 [−0.34, 1.46]
Clifford 2016a	0.55	0.46	10	10	9.4%	0.55 [−0.35, 1.45]
Clifford 2016b (1)	−0.22	0.39	20	10	13.0%	−0.22 [−0.98, 0.54]
Clifford 2017a	0.31	0.34	17	17	17.1%	0.31 [−0.36, 0.98]
Clifford 2017b	0	0.45	10	10	9.8%	0.00 [−0.88, 0.88]
**Subtotal (95% CI)**			**67**	**57**	**58.7%**	**0.22 [−0.14, 0.58]**
Heterogeneity: Tau² = 0.00; Chi² = 2.64, df = 4 (P = 0.62); I² = 0%						
Test for overall effect: Z = 1.19 (P = 0.23)						
**Total (95% CI)**			**90**	**80**	**100.0%**	**0.25 [−0.03, 0.53]**
Heterogeneity: Tau² = 0.00; Chi² = 2.72, df = 6 (P = 0.84); I² = 0%						
Test for overall effect: Z = 1.78 (P = 0.08)						
Test for subgroup differences: Chi² = 0.07, df = 1 (P = 0.79), I² = 0%						

Std. Mean Difference
IV, Random, 95% CI

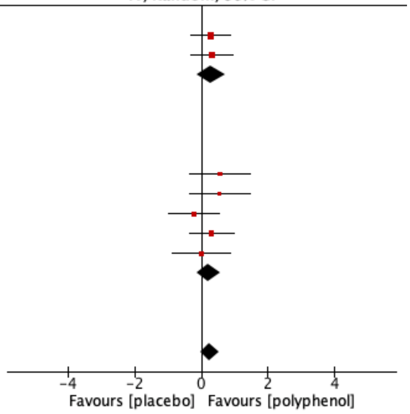

Footnotes
(1) Low and high beetroot combined

(B)

Study or Subgroup	Std. Mean Difference	SE	Polyphenol Total	Placebo Total	Weight	Std. Mean Difference IV, Random, 95% CI
**39.2.1 Cross-over Studies**						
Abbot et al. 2019 (1)	−0.24	0.14	10	10	23.9%	−0.24 [−0.51, 0.03]
Daab et al. 2020	0.41	0.25	13	13	15.9%	0.41 [−0.08, 0.90]
Morgan et al. 2018	0.47	0.23	10	10	17.2%	0.47 [0.02, 0.92]
**Subtotal (95% CI)**			**33**	**33**	**57.1%**	**0.18 [−0.32, 0.69]**
Heterogeneity: Tau² = 0.16; Chi² = 9.69, df = 2 (P = 0.008); I² = 79%						
Test for overall effect: Z = 0.71 (P = 0.48)						
**39.2.2 Parallel studies**						
Brown 2018	0.51	0.46	10	10	7.4%	0.51 [−0.39, 1.41]
Clifford 2016a	0.56	0.46	10	10	7.4%	0.56 [−0.34, 1.46]
Clifford 2016b (2)	0.22	0.39	20	10	9.4%	0.22 [−0.54, 0.98]
Clifford 2017a	0.38	0.34	17	17	11.3%	0.38 [−0.29, 1.05]
Clifford 2017b	0.26	0.45	10	10	7.6%	0.26 [−0.62, 1.14]
**Subtotal (95% CI)**			**67**	**57**	**42.9%**	**0.37 [0.01, 0.73]**
Heterogeneity: Tau² = 0.00; Chi² = 0.47, df = 4 (P = 0.98); I² = 0%						
Test for overall effect: Z = 2.03 (P = 0.04)						
**Total (95% CI)**			**100**	**90**	**100.0%**	**0.25 [−0.03, 0.53]**
Heterogeneity: Tau² = 0.07; Chi² = 12.65, df = 7 (P = 0.08); I² = 45%						
Test for overall effect: Z = 1.75 (P = 0.08)						
Test for subgroup differences: Chi² = 0.36, df = 1 (P = 0.55), I² = 0%						

Std. Mean Difference
IV, Random, 95% CI

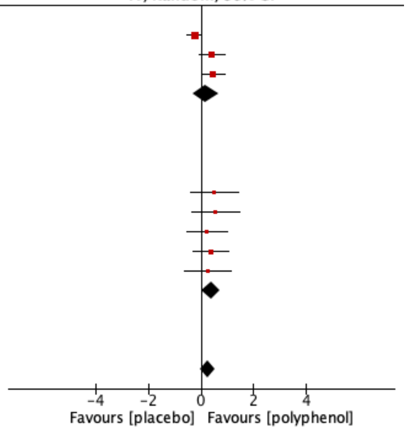

Footnotes
(1) 12 h post
(2) Low and high beetroot combined

(C)

Study or Subgroup	Std. Mean Difference	SE	Polyphenol Total	Placebo Total	Weight	Std. Mean Difference IV, Random, 95% CI
**39.3.1 Cross-over studies**						
Abbot et al. 2019 (1)	−0.1	0.12	10	10	20.7%	−0.10 [−0.34, 0.14]
Daab et al. 2020	0.73	0.36	13	13	12.4%	0.73 [0.02, 1.44]
Morgan et al. 2018	0.5	0.28	10	10	15.1%	0.50 [−0.05, 1.05]
**Subtotal (95% CI)**			**33**	**33**	**48.1%**	**0.31 [−0.24, 0.85]**
Heterogeneity: Tau² = 0.17; Chi² = 7.71, df = 2 (P = 0.02); I² = 74%						
Test for overall effect: Z = 1.09 (P = 0.27)						
**39.3.2 Parallel studies**						
Brown 2018	1.39	0.51	10	10	8.5%	1.39 [0.39, 2.39]
Clifford 2016a	0.91	0.47	10	10	9.4%	0.91 [−0.01, 1.83]
Clifford 2016b (2)	0.62	0.4	20	10	11.2%	0.62 [−0.16, 1.40]
Clifford 2017a	0.3	0.34	17	17	13.0%	0.30 [−0.37, 0.97]
Clifford 2017b	0.32	0.45	10	10	9.8%	0.32 [−0.56, 1.20]
**Subtotal (95% CI)**			**67**	**57**	**51.9%**	**0.62 [0.25, 0.99]**
Heterogeneity: Tau² = 0.00; Chi² = 3.99, df = 4 (P = 0.41); I² = 0%						
Test for overall effect: Z = 3.30 (P = 0.0010)						
**Total (95% CI)**			**100**	**90**	**100.0%**	**0.49 [0.12, 0.86]**
Heterogeneity: Tau² = 0.16; Chi² = 18.54, df = 7 (P = 0.010); I² = 62%						
Test for overall effect: Z = 2.60 (P = 0.009)						
Test for subgroup differences: Chi² = 0.88, df = 1 (P = 0.35), I² = 0%						

Std. Mean Difference
IV, Random, 95% CI

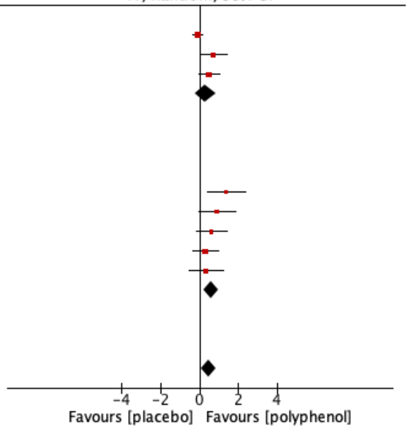

Footnotes
(1) 36 h post
(2) Low and high beetroot combined

(D)

Study or Subgroup	Std. Mean Difference	SE	Polyphenol Total	Placebo Total	Weight	Std. Mean Difference IV, Random, 95% CI
**39.4.1 Cross-over Studies**						
Abbot et al. 2019 (1)	−0.27	0.1	10	10	23.3%	−0.27 [−0.47, −0.07]
Daab et al. 2020	0.43	0.25	13	13	19.9%	0.43 [−0.06, 0.92]
**Subtotal (95% CI)**			**23**	**23**	**43.2%**	**0.04 [−0.64, 0.72]**
Heterogeneity: $Tau^2 = 0.21$; $Chi^2 = 6.76$, df = 1 (P = 0.009); $I^2 = 85\%$						
Test for overall effect: Z = 0.12 (P = 0.90)						
**39.4.2 Parallel Studies**						
Brown 2018	1.02	0.48	10	10	13.6%	1.02 [0.08, 1.96]
Clifford 2016a	1.32	0.51	10	10	12.9%	1.32 [0.32, 2.32]
Clifford 2016b (2)	0.25	0.39	20	10	16.0%	0.25 [−0.51, 1.01]
Clifford 2017b	0.48	0.45	10	10	14.3%	0.48 [−0.40, 1.36]
**Subtotal (95% CI)**			**50**	**40**	**56.8%**	**0.70 [0.22, 1.18]**
Heterogeneity: $Tau^2 = 0.03$; $Chi^2 = 3.49$, df = 3 (P = 0.32); $I^2 = 14\%$						
Test for overall effect: Z = 2.86 (P = 0.004)						
**Total (95% CI)**			**73**	**63**	**100.0%**	**0.44 [−0.09, 0.97]**

Heterogeneity: $Tau^2 = 0.30$; $Chi^2 = 22.69$, df = 5 (P = 0.0004); $I^2 = 78\%$
Test for overall effect: Z = 1.64 (P = 0.10)
Test for subgroup differences: $Chi^2 = 2.38$, df = 1 (P = 0.12), $I^2 = 58.0\%$

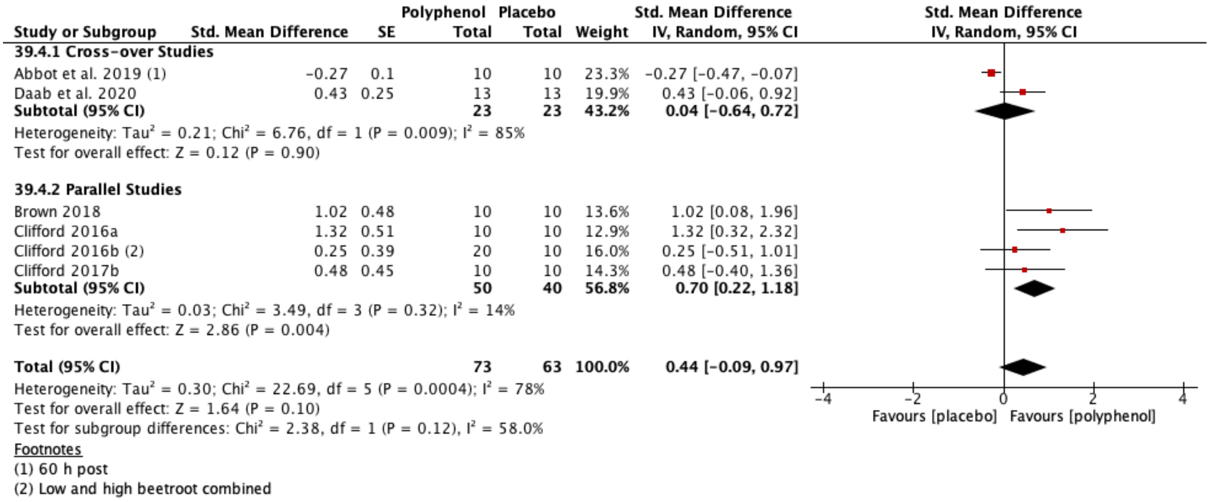

Footnotes
(1) 60 h post
(2) Low and high beetroot combined

Supplementary Figure 4c. Sensitivity analysis: Effect of polyphenol-rich foods, juices and concentrates on recovery of countermovement jump (A) immediately post-exercise; (B) 24 hours; (C) 48 hours; (D) 72 hours after excluding 10 studies classified as high risk of bias.
