# Supplementary material for: Effect of Polyphenol-Rich Foods, Juices, and Concentrates on Recovery from Exercise Induced Muscle Damage: A Systematic Review and Meta-Analysis
Source: Nutrients. 2021 Aug 27;13(9):2988. doi: 10.3390/nu13092988 (PMC8465563; doi:10.3390/nu13092988)
Supplement: Supplementary file 1 [file nutrients-13-02988-s001.zip › Supplementary File 5 CK Forest Plot_edited.pdf]

(A)

Study or Subgroup	Std. Mean Difference	SE	Polyphenol Total	Placebo Total	Weight	Std. Mean Difference IV, Random, 95% CI
**16.1.1 Cross-Over Studies**						
Bowtell 2011	0.98	0.38	10	10	7.7%	0.98 [0.24, 1.72]
Daab et al. 2020	−0.21	0.22	13	13	11.2%	−0.21 [−0.64, 0.22]
Morgan et al. 2018	0.14	0.32	10	10	8.9%	0.14 [−0.49, 0.77]
**Subtotal (95% CI)**			**33**	**33**	**27.8%**	**0.25 [−0.41, 0.91]**
Heterogeneity: Tau² = 0.24; Chi² = 7.36, df = 2 (P = 0.03); I² = 73%						
Test for overall effect: Z = 0.74 (P = 0.46)						
**16.1.2 Parallel Studies**						
Bell 2015	1.24	0.56	8	8	5.0%	1.24 [0.14, 2.34]
Bell 2016	0.7	0.52	8	8	5.5%	0.70 [−0.32, 1.72]
Brown 2018	−0.12	0.45	10	10	6.5%	−0.12 [−1.00, 0.76]
Clifford 2016a	−1.15	0.49	10	10	5.9%	−1.15 [−2.11, −0.19]
Clifford 2016b (1)	−0.76	0.4	20	10	7.3%	−0.76 [−1.54, 0.02]
Clifford 2017a	−0.13	0.34	17	17	8.5%	−0.13 [−0.80, 0.54]
Clifford 2017b	−0.2	0.45	10	10	6.5%	−0.20 [−1.08, 0.68]
Howatson 2010	−0.6	0.46	10	10	6.3%	−0.60 [−1.50, 0.30]
Lamb et al. 2019 (2)	0.16	0.37	23	11	7.9%	0.16 [−0.57, 0.89]
Lynn 2018	0.07	0.46	9	10	6.3%	0.07 [−0.83, 0.97]
Quinlan 2020	−0.58	0.46	10	10	6.3%	−0.58 [−1.48, 0.32]
**Subtotal (95% CI)**			**135**	**114**	**72.2%**	**−0.16 [−0.51, 0.20]**
Heterogeneity: Tau² = 0.16; Chi² = 18.08, df = 10 (P = 0.05); I² = 45%						
Test for overall effect: Z = 0.87 (P = 0.38)						
**Total (95% CI)**			**168**	**147**	**100.0%**	**−0.05 [−0.35, 0.25]**
Heterogeneity: Tau² = 0.17; Chi² = 27.13, df = 13 (P = 0.01); I² = 52%						
Test for overall effect: Z = 0.31 (P = 0.75)						
Test for subgroup differences: Chi² = 1.14, df = 1 (P = 0.29), I² = 12.2%						

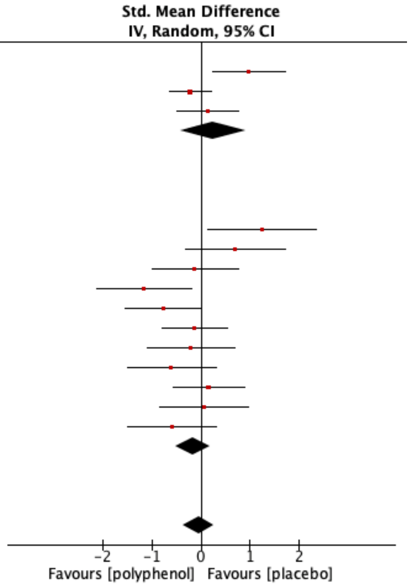

Footnotes
(1) Low and high beetroot combined
(2) Cherry and pomegranate combined

(B)

Study or Subgroup	Std. Mean Difference	SE	Polyphenol Total	Placebo Total	Weight	Std. Mean Difference IV, Random, 95% CI
**16.2.1 Cross-over Studies**						
Bowtell 2011	0.87	0.37	10	10	6.2%	0.87 [0.14, 1.60]
Daab et al. 2020	0.08	0.13	13	13	17.9%	0.08 [−0.17, 0.33]
McLeay 2012 (1)	0.35	0.33	10	10	7.3%	0.35 [−0.30, 1.00]
Morgan et al. 2018	0	0.32	10	10	7.6%	0.00 [−0.63, 0.63]
Peschek 2014	1.25	0.47	8	8	4.2%	1.25 [0.33, 2.17]
**Subtotal (95% CI)**			**51**	**51**	**43.3%**	**0.40 [−0.01, 0.80]**
Heterogeneity: Tau² = 0.12; Chi² = 9.68, df = 4 (P = 0.05); I² = 59%						
Test for overall effect: Z = 1.92 (P = 0.06)						
**16.2.2 Paralell Studies**						
Bell 2015	0.16	0.5	8	8	3.8%	0.16 [−0.82, 1.14]
Bell 2016	0.38	0.51	8	8	3.7%	0.38 [−0.62, 1.38]
Brown 2018	−0.5	0.46	10	10	4.4%	−0.50 [−1.40, 0.40]
Clifford 2016a	−0.12	0.45	10	10	4.6%	−0.12 [−1.00, 0.76]
Clifford 2016b (2)	−0.21	0.39	20	10	5.7%	−0.21 [−0.97, 0.55]
Clifford 2017a	−0.15	0.34	17	17	7.0%	−0.15 [−0.82, 0.52]
Clifford 2017b	0.63	0.46	10	10	4.4%	0.63 [−0.27, 1.53]
Howatson 2010	−0.3	0.45	10	10	4.6%	−0.30 [−1.18, 0.58]
Hutchison 2016	−0.36	0.51	8	8	3.7%	−0.36 [−1.36, 0.64]
Lamb et al. 2019 (3)	0.3	0.37	23	11	6.2%	0.30 [−0.43, 1.03]
Lynn 2018	0.25	0.46	9	10	4.4%	0.25 [−0.65, 1.15]
Quinlan 2020	−0.85	0.47	10	10	4.2%	−0.85 [−1.77, 0.07]
**Subtotal (95% CI)**			**143**	**122**	**56.7%**	**−0.06 [−0.31, 0.19]**
Heterogeneity: Tau² = 0.00; Chi² = 9.19, df = 11 (P = 0.60); I² = 0%						
Test for overall effect: Z = 0.49 (P = 0.62)						
**Total (95% CI)**			**194**	**173**	**100.0%**	**0.11 [−0.10, 0.32]**
Heterogeneity: Tau² = 0.05; Chi² = 21.85, df = 16 (P = 0.15); I² = 27%						
Test for overall effect: Z = 1.04 (P = 0.30)						
Test for subgroup differences: Chi² = 3.58, df = 1 (P = 0.06), I² = 72.1%						

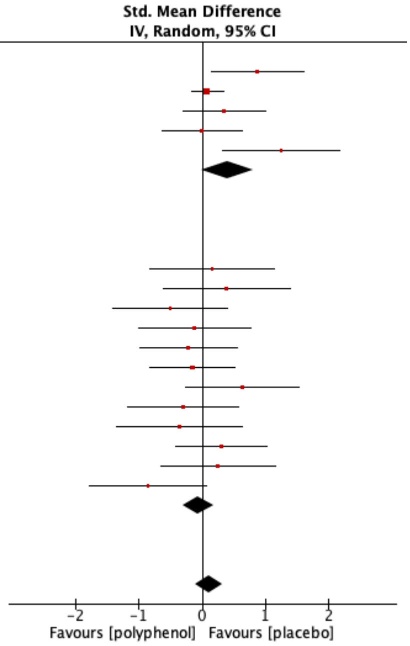

Footnotes
(1) 12 h post
(2) Low and high beetroot combined
(3) Cherry and pomegranate combined

(C)

Study or Subgroup	Std. Mean Difference	SE	Polyphenol Total	Placebo Total	Weight	Std. Mean Difference IV, Random, 95% CI
**16.3.1 Cross-over Studies**						
Bowtell 2011	0.55	0.34	10	10	6.4%	0.55 [-0.12, 1.22]
Daab et al. 2020	0.84	0.16	13	13	9.4%	0.84 [0.53, 1.15]
McLeay 2012 (1)	-0.16	0.32	10	10	6.7%	-0.16 [-0.79, 0.47]
Morgan et al. 2018	0.27	0.32	10	10	6.7%	0.27 [-0.36, 0.90]
Peschek 2014	0.22	0.36	8	8	6.1%	0.22 [-0.49, 0.93]
**Subtotal (95% CI)**			**51**	**51**	**35.2%**	**0.39 [0.00, 0.79]**
Heterogeneity: Tau² = 0.12; Chi² = 9.80, df = 4 (P = 0.04); I² = 59%						
Test for overall effect: Z = 1.96 (P = 0.05)						
**16.3.2 Paralell Studies**						
Bell 2015	0.59	0.51	8	8	4.2%	0.59 [-0.41, 1.59]
Bell 2016	0.4	0.51	8	8	4.2%	0.40 [-0.60, 1.40]
Brown 2018	-0.64	0.46	10	10	4.7%	-0.64 [-1.54, 0.26]
Clifford 2016a	-0.11	0.45	10	10	4.9%	-0.11 [-0.99, 0.77]
Clifford 2016b (2)	-0.5	0.4	20	10	5.5%	-0.50 [-1.28, 0.28]
Clifford 2017a	0.02	0.34	17	17	6.4%	0.02 [-0.65, 0.69]
Clifford 2017b	0.61	0.46	10	10	4.7%	0.61 [-0.29, 1.51]
Howatson 2010	-0.34	0.45	10	10	4.9%	-0.34 [-1.22, 0.54]
Hutchison 2016	-0.92	0.53	8	8	4.0%	-0.92 [-1.96, 0.12]
Lamb et al. 2019 (3)	0.38	0.37	23	11	5.9%	0.38 [-0.35, 1.11]
Lima et al. 2019	-0.44	0.37	15	15	5.9%	-0.44 [-1.17, 0.29]
Lynn 2018	0.01	0.46	9	10	4.7%	0.01 [-0.89, 0.91]
Quinlan 2020	-0.42	0.45	10	10	4.9%	-0.42 [-1.30, 0.46]
**Subtotal (95% CI)**			**158**	**137**	**64.8%**	**-0.10 [-0.35, 0.14]**
Heterogeneity: Tau² = 0.02; Chi² = 13.44, df = 12 (P = 0.34); I² = 11%						
Test for overall effect: Z = 0.82 (P = 0.41)						
**Total (95% CI)**			**209**	**188**	**100.0%**	**0.07 [-0.19, 0.33]**
Heterogeneity: Tau² = 0.16; Chi² = 38.15, df = 17 (P = 0.002); I² = 55%						
Test for overall effect: Z = 0.50 (P = 0.62)						
Test for subgroup differences: Chi² = 4.39, df = 1 (P = 0.04), I² = 77.2%						

Std. Mean Difference
IV, Random, 95% CI

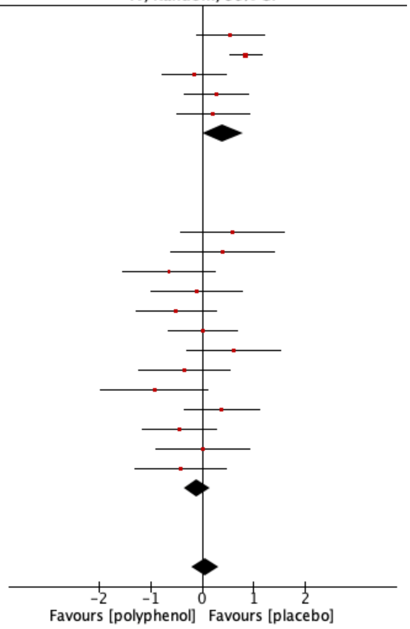

Footnotes
(1) 36 h post
(2) Low and high beetroot combined
(3) Cherry and pomegranate combined

(D)

Study or Subgroup	Std. Mean Difference	SE	Polyphenol Total	Placebo Total	Weight	Std. Mean Difference IV, Random, 95% CI
**16.4.1 Cross-Over Studies**						
Daab et al. 2020	0.34	0.22	13	13	17.3%	0.34 [-0.09, 0.77]
McLeay 2012 (1)	-0.99	0.39	10	10	11.7%	-0.99 [-1.75, -0.23]
**Subtotal (95% CI)**			**23**	**23**	**29.0%**	**-0.29 [-1.59, 1.02]**
Heterogeneity: Tau² = 0.78; Chi² = 8.82, df = 1 (P = 0.003); I² = 89%						
Test for overall effect: Z = 0.43 (P = 0.67)						
**16.4.2 Paralell Studies**						
Bell 2015	0.34	0.51	8	8	8.7%	0.34 [-0.66, 1.34]
Bell 2016	0.42	0.51	8	8	8.7%	0.42 [-0.58, 1.42]
Brown 2018	-0.51	0.46	10	10	9.8%	-0.51 [-1.41, 0.39]
Clifford 2016a	-0.34	0.45	10	10	10.1%	-0.34 [-1.22, 0.54]
Clifford 2016b (2)	-0.9	0.41	20	10	11.1%	-0.90 [-1.70, -0.10]
Clifford 2017b	0.29	0.45	10	10	10.1%	0.29 [-0.59, 1.17]
Lamb et al. 2019 (3)	0.06	0.36	23	11	12.6%	0.06 [-0.65, 0.77]
**Subtotal (95% CI)**			**89**	**67**	**71.0%**	**-0.12 [-0.49, 0.25]**
Heterogeneity: Tau² = 0.05; Chi² = 7.59, df = 6 (P = 0.27); I² = 21%						
Test for overall effect: Z = 0.65 (P = 0.51)						
**Total (95% CI)**			**112**	**90**	**100.0%**	**-0.14 [-0.52, 0.24]**
Heterogeneity: Tau² = 0.17; Chi² = 16.75, df = 8 (P = 0.03); I² = 52%						
Test for overall effect: Z = 0.72 (P = 0.47)						
Test for subgroup differences: Chi² = 0.06, df = 1 (P = 0.81), I² = 0%						

Std. Mean Difference
IV, Random, 95% CI

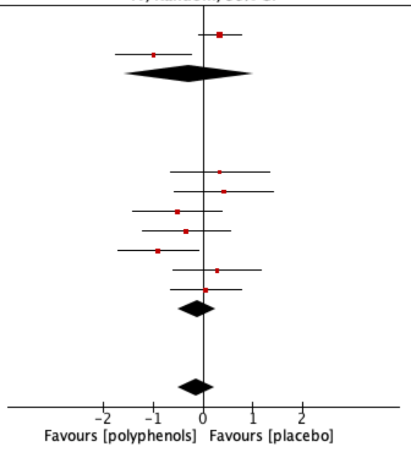

Footnotes
(1) 60 h post
(2) Low and high beetroot combined
(3) Cherry and pomegranate combined

(E)

Study or Subgroup	Std. Mean Difference	SE	Polyphenol Total	Placebo Total	Weight	Std. Mean Difference IV, Random, 95% CI
**16.5.2 Paralell Studies**						
Hutchison 2016	−1.2	0.56	8	8	23.2%	−1.20 [−2.30, −0.10]
Lamb et al. 2019 (1)	−0.09	0.37	23	11	38.9%	−0.09 [−0.82, 0.64]
Lima et al. 2019	−0.82	0.38	15	15	37.8%	−0.82 [−1.56, −0.08]
**Subtotal (95% CI)**			**46**	**34**	**100.0%**	**−0.62 [−1.25, 0.00]**
Heterogeneity: Tau² = 0.13; Chi² = 3.38, df = 2 (P = 0.18); I² = 41%						
Test for overall effect: Z = 1.95 (P = 0.05)						
**Total (95% CI)**			**46**	**34**	**100.0%**	**−0.62 [−1.25, 0.00]**
Heterogeneity: Tau² = 0.13; Chi² = 3.38, df = 2 (P = 0.18); I² = 41%						
Test for overall effect: Z = 1.95 (P = 0.05)						
Test for subgroup differences: Not applicable						

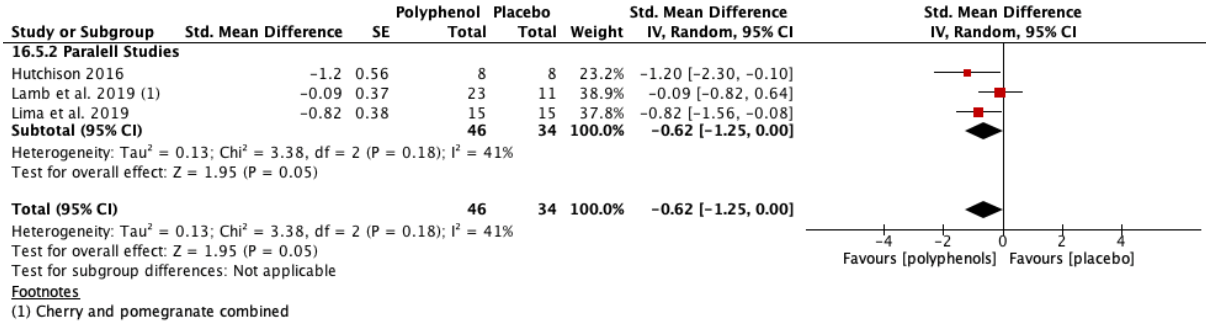

Footnotes
(1) Cherry and pomegranate combined

Supplementary Figure 5. Effect of polyphenol-rich foods, juices and concentrates on creatine kinase (A) immediately post-exercise; (B) 24 hours; (C) 48 hours; (D) 72 hours; (E) 96 hours.
