# Supplementary material for: Effect of Polyphenol-Rich Foods, Juices, and Concentrates on Recovery from Exercise Induced Muscle Damage: A Systematic Review and Meta-Analysis
Source: Nutrients. 2021 Aug 27;13(9):2988. doi: 10.3390/nu13092988 (PMC8465563; doi:10.3390/nu13092988)
Supplement: Supplementary file 1 [file nutrients-13-02988-s001.zip › Supplementary File 6 CRP Forest Plot-edited.pdf]

(A)

Study or Subgroup	Std. Mean Difference	SE	Polyphenol Total	Placebo Total	Weight	Std. Mean Difference IV, Random, 95% CI
**17.1.1 Cross-Over Studies**						
Bowtell 2011	−0.21	0.34	10	10	11.3%	−0.21 [−0.88, 0.46]
Daab et al. 2020	1.58	0.42	13	13	9.2%	1.58 [0.76, 2.40]
Morgan et al. 2018	−0.11	0.32	10	10	11.9%	−0.11 [−0.74, 0.52]
**Subtotal (95% CI)**			**33**	**33**	**32.4%**	**0.39 [−0.65, 1.43]**
Heterogeneity: Tau² = 0.71; Chi² = 13.12, df = 2 (P = 0.001); I² = 85%						
Test for overall effect: Z = 0.74 (P = 0.46)						
**17.1.2 Parallel Studies**						
Bell 2015	−0.02	0.5	8	8	7.4%	−0.02 [−1.00, 0.96]
Bell 2016	−0.56	0.51	8	8	7.2%	−0.56 [−1.56, 0.44]
Brown 2018	−0.01	0.45	10	10	8.5%	−0.01 [−0.89, 0.87]
Clifford 2016a	0.23	0.45	10	10	8.5%	0.23 [−0.65, 1.11]
Clifford 2017a	0.07	0.35	16	16	11.0%	0.07 [−0.62, 0.76]
Clifford 2017b	−0.14	0.45	10	10	8.5%	−0.14 [−1.02, 0.74]
Lynn 2018	−0.39	0.46	9	10	8.2%	−0.39 [−1.29, 0.51]
Quinlan 2020	−0.5	0.46	10	10	8.2%	−0.50 [−1.40, 0.40]
**Subtotal (95% CI)**			**81**	**82**	**67.6%**	**−0.14 [−0.44, 0.17]**
Heterogeneity: Tau² = 0.00; Chi² = 2.76, df = 7 (P = 0.91); I² = 0%						
Test for overall effect: Z = 0.86 (P = 0.39)						
**Total (95% CI)**			**114**	**115**	**100.0%**	**0.01 [−0.33, 0.34]**
Heterogeneity: Tau² = 0.14; Chi² = 18.15, df = 10 (P = 0.05); I² = 45%						
Test for overall effect: Z = 0.04 (P = 0.97)						
Test for subgroup differences: Chi² = 0.91, df = 1 (P = 0.34), I² = 0%						

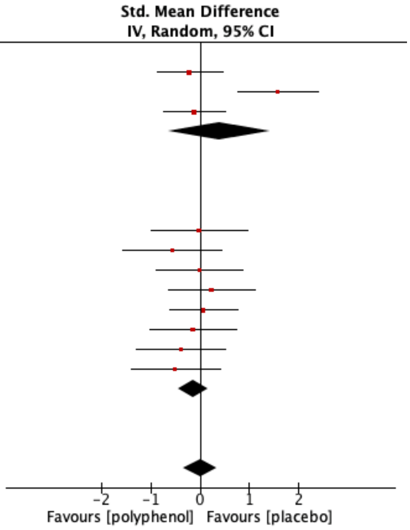

(B)

Study or Subgroup	Std. Mean Difference	SE	Polyphenol Total	Placebo Total	Weight	Std. Mean Difference IV, Random, 95% CI
**17.2.1 Cross-over Studies**						
Bowtell 2011	−0.19	0.34	10	10	9.9%	−0.19 [−0.86, 0.48]
Daab et al. 2020	1.39	0.39	13	13	9.0%	1.39 [0.63, 2.15]
Morgan et al. 2018	0.11	0.32	10	10	10.2%	0.11 [−0.52, 0.74]
**Subtotal (95% CI)**			**33**	**33**	**29.1%**	**0.42 [−0.48, 1.31]**
Heterogeneity: Tau² = 0.50; Chi² = 10.19, df = 2 (P = 0.006); I² = 80%						
Test for overall effect: Z = 0.91 (P = 0.36)						
**17.2.2 Paralell Studies**						
Bell 2015	0.18	0.5	8	8	7.3%	0.18 [−0.80, 1.16]
Bell 2016	−0.46	0.51	8	8	7.1%	−0.46 [−1.46, 0.54]
Brown 2018	0.33	0.45	10	10	8.0%	0.33 [−0.55, 1.21]
Clifford 2016a	0.21	0.45	10	10	8.0%	0.21 [−0.67, 1.09]
Clifford 2017a	0.09	0.35	16	16	9.7%	0.09 [−0.60, 0.78]
Clifford 2017b	−0.29	0.45	10	10	8.0%	−0.29 [−1.17, 0.59]
Howatson 2010	−1.51	0.52	10	10	7.0%	−1.51 [−2.53, −0.49]
Lynn 2018	0.52	0.47	9	10	7.7%	0.52 [−0.40, 1.44]
Quinlan 2020	−0.72	0.46	10	10	7.9%	−0.72 [−1.62, 0.18]
**Subtotal (95% CI)**			**91**	**92**	**70.9%**	**−0.15 [−0.54, 0.24]**
Heterogeneity: Tau² = 0.14; Chi² = 13.54, df = 8 (P = 0.09); I² = 41%						
Test for overall effect: Z = 0.77 (P = 0.44)						
**Total (95% CI)**			**124**	**125**	**100.0%**	**0.00 [−0.37, 0.38]**
Heterogeneity: Tau² = 0.26; Chi² = 27.30, df = 11 (P = 0.004); I² = 60%						
Test for overall effect: Z = 0.02 (P = 0.98)						
Test for subgroup differences: Chi² = 1.31, df = 1 (P = 0.25), I² = 23.8%						

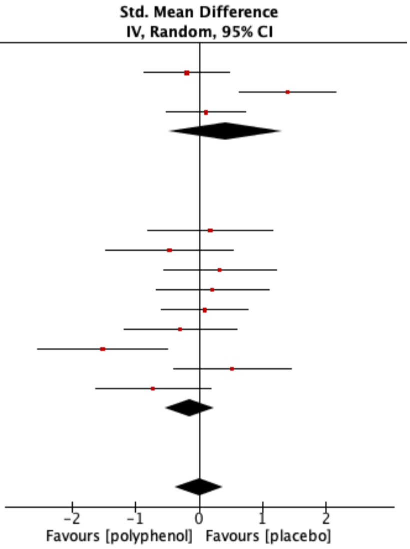

(C)

Study or Subgroup	Std. Mean Difference	SE	Polyphenol Total	Placebo Total	Weight	Std. Mean Difference IV, Random, 95% CI
**17.3.1 Cross-over Studies**						
Bowtell 2011	−0.17	0.34	10	10	10.0%	−0.17 [−0.84, 0.50]
Daab et al. 2020	1.02	0.34	13	13	10.0%	1.02 [0.35, 1.69]
Morgan et al. 2018	0.35	0.33	10	10	10.2%	0.35 [−0.30, 1.00]
**Subtotal (95% CI)**			**33**	**33**	**30.1%**	**0.40 [−0.27, 1.07]**
Heterogeneity: Tau² = 0.24; Chi² = 6.16, df = 2 (P = 0.05); I² = 68%						
Test for overall effect: Z = 1.17 (P = 0.24)						
**17.3.2 Paralell Studies**						
Bell 2015	0.14	0.5	8	8	7.1%	0.14 [−0.84, 1.12]
Bell 2016	−0.69	0.52	8	8	6.9%	−0.69 [−1.71, 0.33]
Brown 2018	0.5	0.45	10	10	7.9%	0.50 [−0.38, 1.38]
Clifford 2016a	0.51	0.46	10	10	7.8%	0.51 [−0.39, 1.41]
Clifford 2017a	0.22	0.35	16	16	9.8%	0.22 [−0.47, 0.91]
Clifford 2017b	0.38	0.45	10	10	7.9%	0.38 [−0.50, 1.26]
Howatson 2010	−1.52	0.52	10	10	6.9%	−1.52 [−2.54, −0.50]
Lynn 2018	−0.49	0.47	9	10	7.6%	−0.49 [−1.41, 0.43]
Quinlan 2020	−0.36	0.45	10	10	7.9%	−0.36 [−1.24, 0.52]
**Subtotal (95% CI)**			**91**	**92**	**69.9%**	**−0.11 [−0.52, 0.31]**
Heterogeneity: Tau² = 0.19; Chi² = 15.47, df = 8 (P = 0.05); I² = 48%						
Test for overall effect: Z = 0.50 (P = 0.62)						
**Total (95% CI)**			**124**	**125**	**100.0%**	**0.04 [−0.32, 0.41]**
Heterogeneity: Tau² = 0.22; Chi² = 25.25, df = 11 (P = 0.008); I² = 56%						
Test for overall effect: Z = 0.24 (P = 0.81)						
Test for subgroup differences: Chi² = 1.59, df = 1 (P = 0.21), I² = 37.0%						

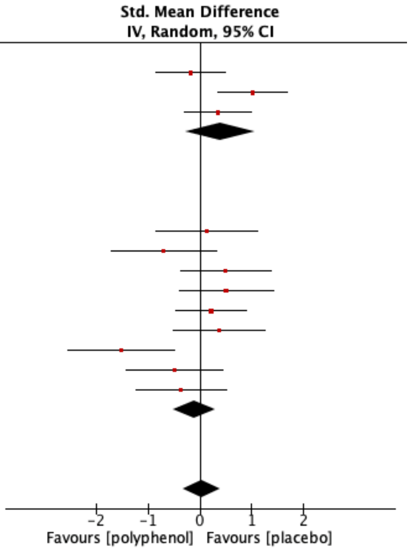

(D)

Study or Subgroup	Std. Mean Difference	SE	Polyphenol Total	Placebo Total	Weight	Std. Mean Difference IV, Random, 95% CI
**17.4.1 Cross-Over Studies**						
Daab et al. 2020	−0.5	0.29	13	13	29.7%	−0.50 [−1.07, 0.07]
**Subtotal (95% CI)**			**13**	**13**	**29.7%**	**−0.50 [−1.07, 0.07]**
Heterogeneity: Not applicable						
Test for overall effect: Z = 1.72 (P = 0.08)						
**17.4.2 Paralell Studies**						
Bell 2015	0.03	0.5	8	8	12.7%	0.03 [−0.95, 1.01]
Bell 2016	−0.73	0.52	8	8	11.9%	−0.73 [−1.75, 0.29]
Brown 2018	−0.11	0.45	10	10	15.2%	−0.11 [−0.99, 0.77]
Clifford 2016a	0.43	0.45	10	10	15.2%	0.43 [−0.45, 1.31]
Clifford 2017b	0.42	0.45	10	10	15.2%	0.42 [−0.46, 1.30]
**Subtotal (95% CI)**			**46**	**46**	**70.3%**	**0.05 [−0.37, 0.46]**
Heterogeneity: $Tau^2 = 0.00$; $Chi^2 = 3.77$, df = 4 (P = 0.44); $I^2 = 0\%$						
Test for overall effect: Z = 0.23 (P = 0.82)						
**Total (95% CI)**			**59**	**59**	**100.0%**	**−0.12 [−0.50, 0.26]**
Heterogeneity: $Tau^2 = 0.04$; $Chi^2 = 6.10$, df = 5 (P = 0.30); $I^2 = 18\%$						
Test for overall effect: Z = 0.62 (P = 0.54)						
Test for subgroup differences: $Chi^2 = 2.33$, df = 1 (P = 0.13), $I^2 = 57.2\%$						

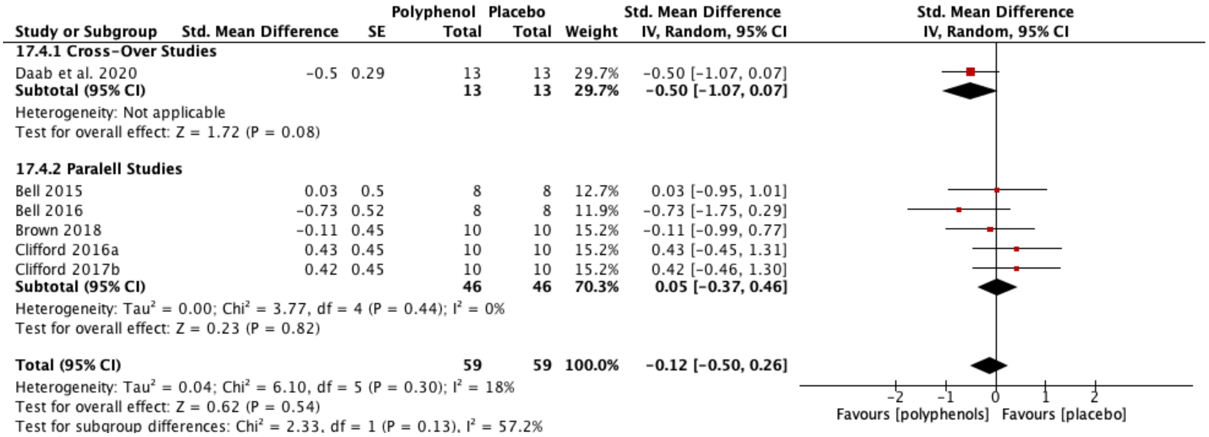

Supplementary Figure 6. Effect of polyphenol-rich foods, juices and concentrates on c-reactive protein (A) immediately post-exercise; (B) 24 hours; (C) 48 hours; (D) 72 hours.
