# Supplementary material for: Effect of Polyphenol-Rich Foods, Juices, and Concentrates on Recovery from Exercise Induced Muscle Damage: A Systematic Review and Meta-Analysis
Source: Nutrients. 2021 Aug 27;13(9):2988. doi: 10.3390/nu13092988 (PMC8465563; doi:10.3390/nu13092988)
Supplement: Supplementary file 1 [file nutrients-13-02988-s001.zip › Supplementary File 7 IL-6 Forest Plot_edited.pdf]

(A)

Study or Subgroup	Std. Mean Difference	SE	Polyphenol Total	Placebo Total	Weight	Std. Mean Difference IV, Random, 95% CI
**18.1.1 Cross-Over Studies**						
Morehen 2020	−0.42	0.18	11	11	22.8%	−0.42 [−0.77, −0.07]
Morgan et al. 2018	0.05	0.32	10	10	16.9%	0.05 [−0.58, 0.68]
**Subtotal (95% CI)**			**21**	**21**	**39.6%**	**−0.26 [−0.70, 0.18]**
Heterogeneity: Tau^2 = 0.04; Chi^2 = 1.64, df = 1 (P = 0.20); I^2 = 39%						
Test for overall effect: Z = 1.16 (P = 0.24)						
**18.1.2 Parallel Studies**						
Bell 2015	−0.77	0.53	8	8	10.2%	−0.77 [−1.81, 0.27]
Bell 2016	−1.16	0.55	8	8	9.7%	−1.16 [−2.24, −0.08]
Clifford 2016b (1)	0.57	0.4	20	10	13.9%	0.57 [−0.21, 1.35]
Clifford 2017a	−0.01	0.35	16	16	15.7%	−0.01 [−0.70, 0.68]
Howatson 2010	−1.27	0.5	10	10	10.9%	−1.27 [−2.25, −0.29]
**Subtotal (95% CI)**			**62**	**52**	**60.4%**	**−0.46 [−1.17, 0.24]**
Heterogeneity: Tau^2 = 0.43; Chi^2 = 12.40, df = 4 (P = 0.01); I^2 = 68%						
Test for overall effect: Z = 1.29 (P = 0.20)						
**Total (95% CI)**			**83**	**73**	**100.0%**	**−0.34 [−0.76, 0.08]**
Heterogeneity: Tau^2 = 0.17; Chi^2 = 14.04, df = 6 (P = 0.03); I^2 = 57%						
Test for overall effect: Z = 1.59 (P = 0.11)						
Test for subgroup differences: Chi^2 = 0.23, df = 1 (P = 0.63), I^2 = 0%						

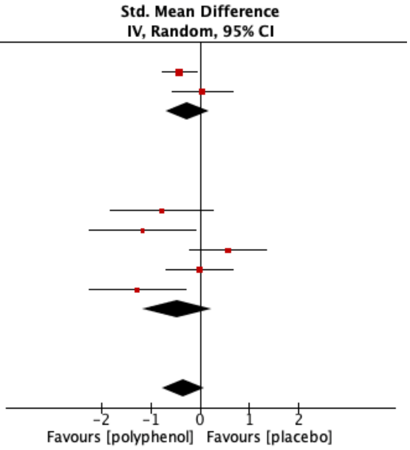

Footnotes
(1) Low and high beetroot combined

(B)

Study or Subgroup	Std. Mean Difference	SE	Polyphenol Total	Placebo Total	Weight	Std. Mean Difference IV, Random, 95% CI
**18.2.1 Cross-over Studies**						
McLeay 2012 (1)	−0.4	0.33	10	10	17.4%	−0.40 [−1.05, 0.25]
Morgan et al. 2018	0.24	0.32	10	10	17.7%	0.24 [−0.39, 0.87]
**Subtotal (95% CI)**			**20**	**20**	**35.1%**	**−0.07 [−0.70, 0.55]**
Heterogeneity: Tau^2 = 0.10; Chi^2 = 1.94, df = 1 (P = 0.16); I^2 = 48%						
Test for overall effect: Z = 0.23 (P = 0.81)						
**18.2.2 Paralell Studies**						
Bell 2015	0.43	0.51	8	8	11.8%	0.43 [−0.57, 1.43]
Bell 2016	−1.43	0.58	8	8	10.2%	−1.43 [−2.57, −0.29]
Clifford 2016b (2)	−0.25	0.39	20	10	15.3%	−0.25 [−1.01, 0.51]
Clifford 2017a	0.51	0.36	16	16	16.3%	0.51 [−0.20, 1.22]
Hutchison 2016	−0.92	0.53	8	8	11.3%	−0.92 [−1.96, 0.12]
**Subtotal (95% CI)**			**60**	**50**	**64.9%**	**−0.27 [−0.96, 0.43]**
Heterogeneity: Tau^2 = 0.40; Chi^2 = 11.70, df = 4 (P = 0.02); I^2 = 66%						
Test for overall effect: Z = 0.75 (P = 0.45)						
**Total (95% CI)**			**80**	**70**	**100.0%**	**−0.18 [−0.64, 0.28]**
Heterogeneity: Tau^2 = 0.21; Chi^2 = 13.70, df = 6 (P = 0.03); I^2 = 56%						
Test for overall effect: Z = 0.76 (P = 0.44)						
Test for subgroup differences: Chi^2 = 0.16, df = 1 (P = 0.69), I^2 = 0%						

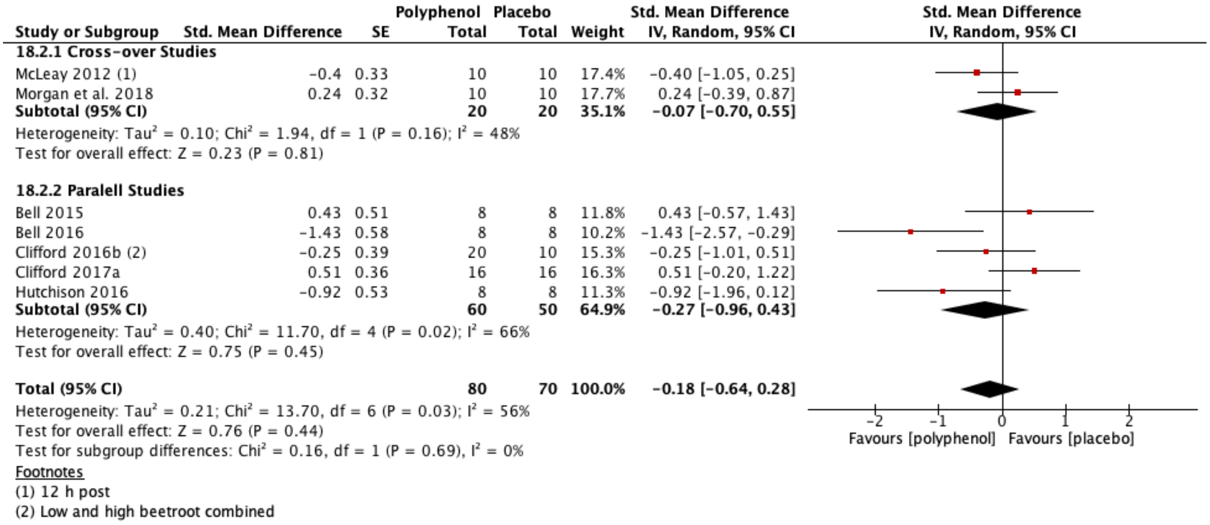

Footnotes
(1) 12 h post
(2) Low and high beetroot combined

(C)

Study or Subgroup	Std. Mean Difference	SE	Polyphenol Total	Placebo Total	Weight	Std. Mean Difference IV, Random, 95% CI
**18.3.1 Cross-over Studies**						
McLeay 2012 (1)	−0.2	0.32	10	10	14.0%	−0.20 [−0.83, 0.43]
Morehen 2020	0.42	0.09	11	11	17.8%	0.42 [0.24, 0.60]
Morgan et al. 2018	0.2	0.32	10	10	14.0%	0.20 [−0.43, 0.83]
**Subtotal (95% CI)**			**31**	**31**	**45.8%**	**0.24 [−0.12, 0.59]**
Heterogeneity: Tau^2 = 0.05; Chi^2 = 3.76, df = 2 (P = 0.15); I^2 = 47%						
Test for overall effect: Z = 1.31 (P = 0.19)						
**18.3.2 Paralell Studies**						
Bell 2015	−1.33	0.57	8	8	9.3%	−1.33 [−2.45, −0.21]
Bell 2016	−1.47	0.58	8	8	9.2%	−1.47 [−2.61, −0.33]
Clifford 2016b (2)	−0.52	0.39	20	10	12.6%	−0.52 [−1.28, 0.24]
Clifford 2017a	0.94	0.38	16	16	12.8%	0.94 [0.20, 1.68]
Hutchison 2016	−0.59	0.51	8	8	10.3%	−0.59 [−1.59, 0.41]
**Subtotal (95% CI)**			**60**	**50**	**54.2%**	**−0.54 [−1.43, 0.36]**
Heterogeneity: Tau^2 = 0.81; Chi^2 = 18.61, df = 4 (P = 0.0009); I^2 = 79%						
Test for overall effect: Z = 1.17 (P = 0.24)						
**Total (95% CI)**			**91**	**81**	**100.0%**	**−0.19 [−0.68, 0.30]**
Heterogeneity: Tau^2 = 0.34; Chi^2 = 31.91, df = 7 (P < 0.0001); I^2 = 78%						
Test for overall effect: Z = 0.76 (P = 0.45)						
Test for subgroup differences: Chi^2 = 2.48, df = 1 (P = 0.12), I^2 = 59.7%						

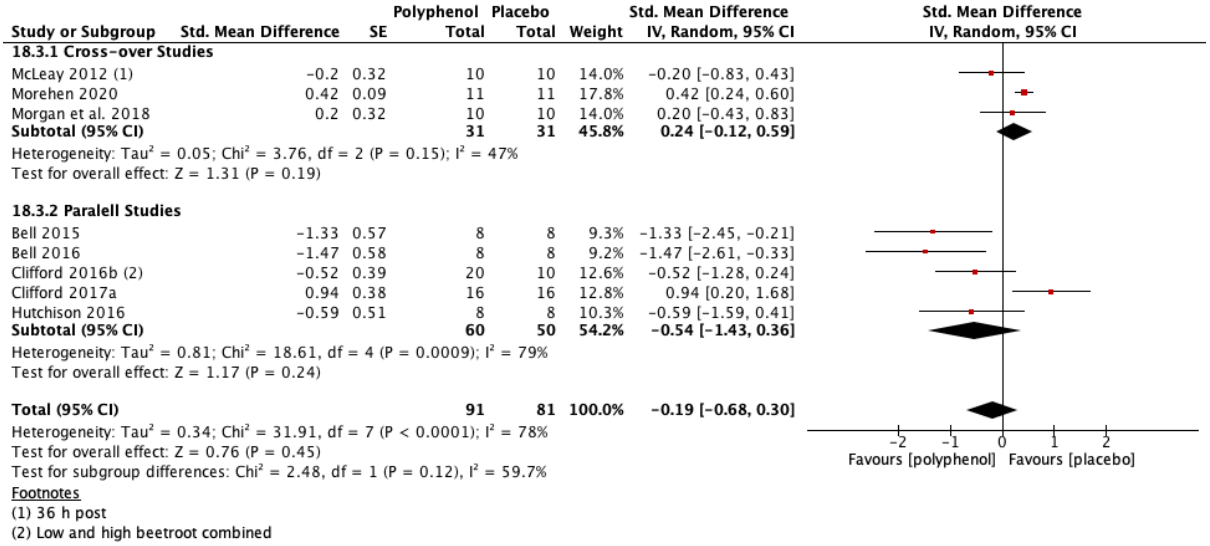

Footnotes
(1) 36 h post
(2) Low and high beetroot combined

(D)

Study or Subgroup	Std. Mean Difference	SE	Polyphenol Total	Placebo Total	Weight	Std. Mean Difference IV, Random, 95% CI
**18.4.1 Cross-Over Studies**						
McLeay 2012 (1)	0.18	0.32	10	10	31.1%	0.18 [−0.45, 0.81]
**Subtotal (95% CI)**			**10**	**10**	**31.1%**	**0.18 [−0.45, 0.81]**
Heterogeneity: Not applicable						
Test for overall effect: Z = 0.56 (P = 0.57)						
**18.4.2 Paralell Studies**						
Bell 2015	−0.42	0.51	8	8	22.3%	−0.42 [−1.42, 0.58]
Bell 2016	−1.29	0.57	8	8	20.0%	−1.29 [−2.41, −0.17]
Clifford 2016b (2)	−0.98	0.41	20	10	26.7%	−0.98 [−1.78, −0.18]
**Subtotal (95% CI)**			**36**	**26**	**68.9%**	**−0.89 [−1.43, −0.34]**
Heterogeneity: $Tau^2 = 0.00$; $Chi^2 = 1.39$, df = 2 (P = 0.50); $I^2 = 0\%$						
Test for overall effect: Z = 3.18 (P = 0.001)						
**Total (95% CI)**			**46**	**36**	**100.0%**	**−0.56 [−1.25, 0.13]**
Heterogeneity: $Tau^2 = 0.30$; $Chi^2 = 7.71$, df = 3 (P = 0.05); $I^2 = 61\%$						
Test for overall effect: Z = 1.58 (P = 0.11)						
Test for subgroup differences: $Chi^2 = 6.32$, df = 1 (P = 0.01), $I^2 = 84.2\%$						

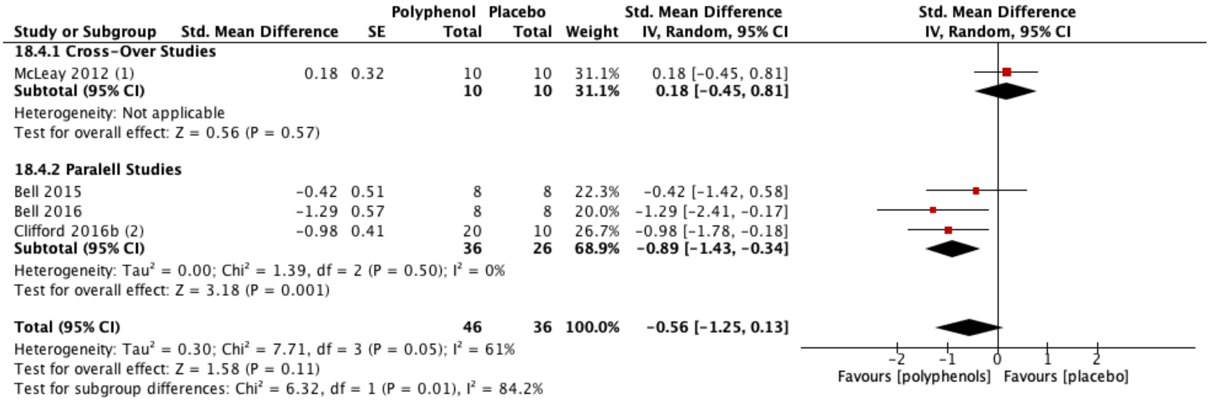

Footnotes
(1) 60 h post
(2) Low and high beetroot combined

(E)

Study or Subgroup	Std. Mean Difference	SE	Polyphenol Total	Placebo Total	Weight	Std. Mean Difference IV, Random, 95% CI
**18.5.2 Paralell Studies**						
Hutchison 2016	−0.76	0.53	8	8	100.0%	−0.76 [−1.80, 0.28]
**Subtotal (95% CI)**			**8**	**8**	**100.0%**	**−0.76 [−1.80, 0.28]**
Heterogeneity: Not applicable						
Test for overall effect: Z = 1.43 (P = 0.15)						
**Total (95% CI)**			**8**	**8**	**100.0%**	**−0.76 [−1.80, 0.28]**
Heterogeneity: Not applicable						
Test for overall effect: Z = 1.43 (P = 0.15)						
Test for subgroup differences: Not applicable						

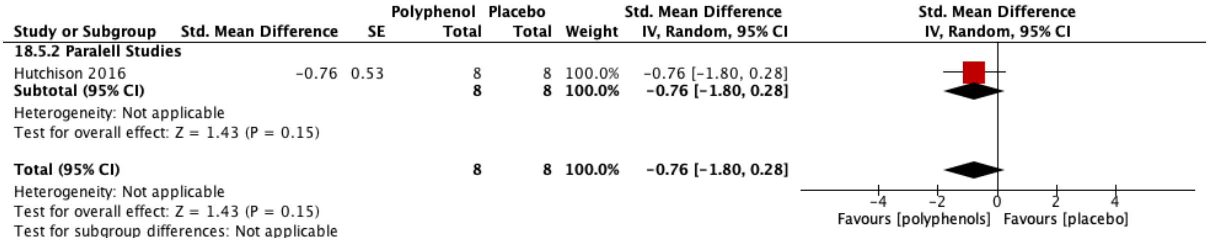

Supplementary Figure 7. Effect of polyphenol-rich foods, juices and concentrates on interleukin-6 ~~at~~ (A) immediately post-exercise; (B) 24 hours; (C) 48 hours; (D) 72 hours; (E) 96 hours.
