# Supplementary material for: Effect of Polyphenol-Rich Foods, Juices, and Concentrates on Recovery from Exercise Induced Muscle Damage: A Systematic Review and Meta-Analysis
Source: Nutrients. 2021 Aug 27;13(9):2988. doi: 10.3390/nu13092988 (PMC8465563; doi:10.3390/nu13092988)
Supplement: Supplementary file 1 [file nutrients-13-02988-s001.zip › Supplementary File 8 PC Forest Plot_edited.pdf]

(A)

Study or Subgroup	Std. Mean Difference	SE	Polyphenol Total	Placebo Total	Weight	Std. Mean Difference IV, Random, 95% CI
**19.1.1 Cross-Over Studies**						
Bowtell 2011	3.97	1.05	8	8	15.8%	3.97 [1.91, 6.03]
Morgan et al. 2018	−0.06	0.32	10	10	29.8%	−0.06 [−0.69, 0.57]
**Subtotal (95% CI)**			**18**	**18**	**45.5%**	**1.83 [−2.11, 5.77]**
Heterogeneity: Tau² = 7.52; Chi² = 13.48, df = 1 (P = 0.0002); I² = 93%						
Test for overall effect: Z = 0.91 (P = 0.36)						
**19.1.2 Parallel Studies**						
Clifford 2016a	−0.68	0.46	10	10	27.1%	−0.68 [−1.58, 0.22]
Howatson 2010	0.15	0.45	10	10	27.3%	0.15 [−0.73, 1.03]
**Subtotal (95% CI)**			**20**	**20**	**54.5%**	**−0.26 [−1.07, 0.55]**
Heterogeneity: Tau² = 0.14; Chi² = 1.66, df = 1 (P = 0.20); I² = 40%						
Test for overall effect: Z = 0.63 (P = 0.53)						
**Total (95% CI)**			**38**	**38**	**100.0%**	**0.46 [−0.67, 1.60]**
Heterogeneity: Tau² = 1.02; Chi² = 16.61, df = 3 (P = 0.0008); I² = 82%						
Test for overall effect: Z = 0.80 (P = 0.42)						
Test for subgroup differences: Chi² = 1.04, df = 1 (P = 0.31), I² = 3.5%						

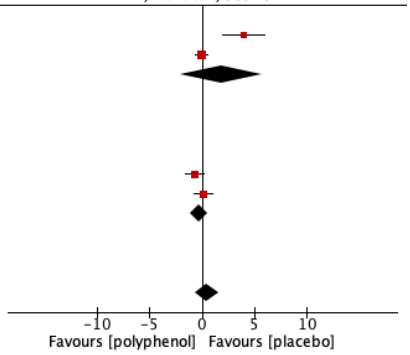

(B)

Study or Subgroup	Std. Mean Difference	SE	Polyphenol Total	Placebo Total	Weight	Std. Mean Difference IV, Random, 95% CI
**19.2.1 Cross-over Studies**						
Bowtell 2011	1.98	0.61	8	8	14.6%	1.98 [0.78, 3.18]
McLeay 2012 (1)	0.07	0.32	10	10	23.8%	0.07 [−0.56, 0.70]
Morgan et al. 2018	−0.15	0.32	10	10	23.8%	−0.15 [−0.78, 0.48]
**Subtotal (95% CI)**			**28**	**28**	**62.1%**	**0.49 [−0.50, 1.48]**
Heterogeneity: Tau² = 0.59; Chi² = 9.88, df = 2 (P = 0.007); I² = 80%						
Test for overall effect: Z = 0.98 (P = 0.33)						
**19.2.2 Paralell Studies**						
Clifford 2016a	0.77	0.47	10	10	18.6%	0.77 [−0.15, 1.69]
Howatson 2010	−0.13	0.45	10	10	19.3%	−0.13 [−1.01, 0.75]
**Subtotal (95% CI)**			**20**	**20**	**37.9%**	**0.31 [−0.57, 1.19]**
Heterogeneity: Tau² = 0.19; Chi² = 1.91, df = 1 (P = 0.17); I² = 48%						
Test for overall effect: Z = 0.69 (P = 0.49)						
**Total (95% CI)**			**48**	**48**	**100.0%**	**0.39 [−0.24, 1.01]**
Heterogeneity: Tau² = 0.33; Chi² = 11.85, df = 4 (P = 0.02); I² = 66%						
Test for overall effect: Z = 1.22 (P = 0.22)						
Test for subgroup differences: Chi² = 0.07, df = 1 (P = 0.78), I² = 0%						

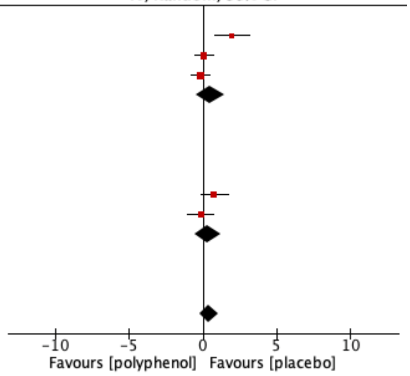

Footnotes
(1) 12 h post

(C)

Study or Subgroup	Std. Mean Difference	SE	Polyphenol Total	Placebo Total	Weight	Std. Mean Difference IV, Random, 95% CI
**19.3.1 Cross-over Studies**						
Bowtell 2011	1.74	0.56	8	8	15.2%	1.74 [0.64, 2.84]
McLeay 2012 (1)	−0.19	0.32	10	10	23.8%	−0.19 [−0.82, 0.44]
Morgan et al. 2018	0.13	0.32	10	10	23.8%	0.13 [−0.50, 0.76]
**Subtotal (95% CI)**			**28**	**28**	**62.8%**	**0.45 [−0.48, 1.38]**
Heterogeneity: Tau² = 0.51; Chi² = 9.09, df = 2 (P = 0.01); I² = 78%						
Test for overall effect: Z = 0.95 (P = 0.34)						
**19.3.2 Paralell Studies**						
Clifford 2016a	0.63	0.46	10	10	18.4%	0.63 [−0.27, 1.53]
Howatson 2010	−0.19	0.45	10	10	18.8%	−0.19 [−1.07, 0.69]
**Subtotal (95% CI)**			**20**	**20**	**37.2%**	**0.21 [−0.59, 1.02]**
Heterogeneity: Tau² = 0.13; Chi² = 1.62, df = 1 (P = 0.20); I² = 38%						
Test for overall effect: Z = 0.52 (P = 0.60)						
**Total (95% CI)**			**48**	**48**	**100.0%**	**0.33 [−0.25, 0.92]**
Heterogeneity: Tau² = 0.27; Chi² = 10.71, df = 4 (P = 0.03); I² = 63%						
Test for overall effect: Z = 1.11 (P = 0.27)						
Test for subgroup differences: Chi² = 0.14, df = 1 (P = 0.71), I² = 0%						

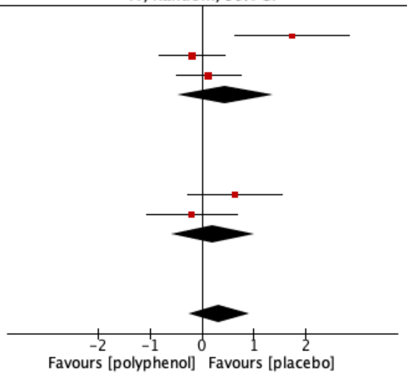

Footnotes
(1) 36 h post

(D)

Study or Subgroup	Std. Mean Difference	SE	Polyphenol Total	Placebo Total	Weight	Std. Mean Difference IV, Random, 95% CI
**19.4.1 Cross-Over Studies**						
McLeay 2012 (1)	−0.69	0.31	10	10	68.8%	−0.69 [−1.30, −0.08]
**Subtotal (95% CI)**			**10**	**10**	**68.8%**	**−0.69 [−1.30, −0.08]**
Heterogeneity: Not applicable						
Test for overall effect: Z = 2.23 (P = 0.03)						
**19.4.2 Paralell Studies**						
Clifford 2016a	−0.53	0.46	10	10	31.2%	−0.53 [−1.43, 0.37]
**Subtotal (95% CI)**			**10**	**10**	**31.2%**	**−0.53 [−1.43, 0.37]**
Heterogeneity: Not applicable						
Test for overall effect: Z = 1.15 (P = 0.25)						
**Total (95% CI)**			**20**	**20**	**100.0%**	**−0.64 [−1.14, −0.14]**

Heterogeneity: $Tau^2 = 0.00$; $Chi^2 = 0.08$, df = 1 (P = 0.77); $I^2 = 0\%$
Test for overall effect: Z = 2.49 (P = 0.01)
Test for subgroup differences: $Chi^2 = 0.08$, df = 1 (P = 0.77), $I^2 = 0\%$

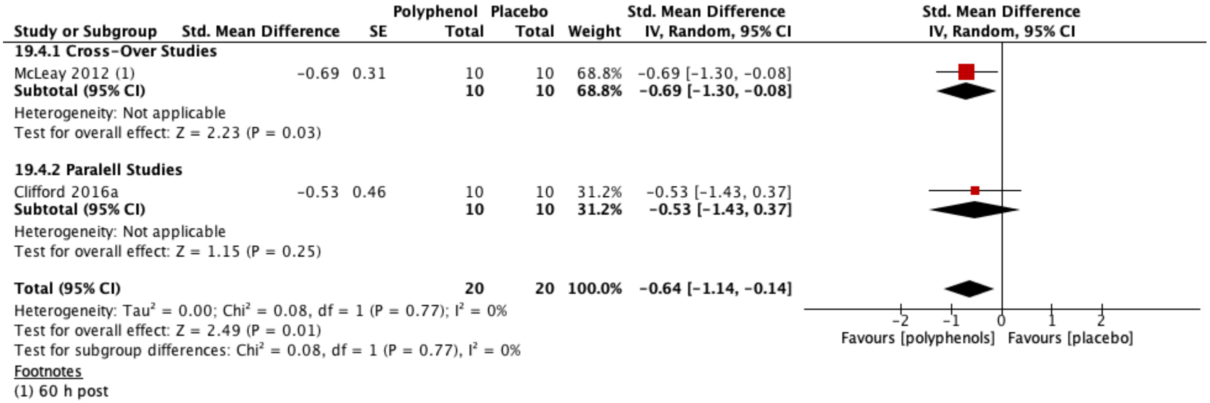

Footnotes
(1) 60 h post

Supplementary Figure 8. Effect of polyphenol-rich foods, juices and concentrates on protein carbonyls ~~at~~ (A) immediately post-exercise; (B) 24 hours; (C) 48 hours; (D) 72 hours.
