# Supplementary material for: Effect of Polyphenol-Rich Foods, Juices, and Concentrates on Recovery from Exercise Induced Muscle Damage: A Systematic Review and Meta-Analysis
Source: Nutrients. 2021 Aug 27;13(9):2988. doi: 10.3390/nu13092988 (PMC8465563; doi:10.3390/nu13092988)
Supplement: Supplementary file 1 [file nutrients-13-02988-s001.zip › Supplementary File 9 Analyses by individual polyphenol rich foods_edited.pdf]

Supplementary File 9. Analyses by individual polyphenols

Table S1. Effect of individual polyphenol-rich foods, juices and concentrates on maximal voluntary isometric contraction (MIVC)

**MIVC**	**Cherry**	**POM**	**Cocoa**	**Beetroot**
Immediately post	0.31 (-0.06, 0.68); P = 0.10, I^2 = 0%	0.03 (-0.33, 0.40); P = 0.85, I^2 = 0%		0.29 (-0.04, 0.62); P = 0.08, I^2 = 0%
24 hrs	0.59 (0.06, 1.12); P = 0.03, I^2 = 64%	0.27 (-0.04, 0.58); P = 0.08, I^2 = 0%	0.03 (-0.42, 0.48); P = 0.91, I^2 = 0%	0.41 (-0.17, 0.99); P = 0.17, I^2 = 62%
48 hrs	0.67 (0.23, 1.11); P = 0.003, I^2 = 50%	0.23 (-0.15, 0.61); P = 0.24, I^2 = 29%	0.50 (0.09, 0.91); P = 0.02, I^2 = 0%	0.34 (-0.06, 0.75); P = 0.10, I^2 = 29%
72 hrs	0.41 (-0.08, 0.90); P = 0.10, I^2 = 38%	0.21 (-0.04, 0.46); P = 0.10, I^2 = 0%		0.28 (-0.09, 0.64); P = 0.14, I^2 = 0%
96 hrs	0.79 (-0.38, 1.95); P = 0.19, I^2 = 77%	0.37 (0.11, 0.63); P = 0.006, I^2 = 0%		

Data presented as SMD (95% CI)

Table S2. Effect of individual polyphenol-rich foods, juices and concentrates on delayed onset muscle soreness (DOMS)

**DOMS**	**Cherry**	**POM**	**Cocoa**	**Beetroot**
Immediately post	-0.32 (-0.92, 0.28); P = 0.30, I^2 = 75	0.14 (-0.31, 0.59); P = 0.54, I^2 = 37%		-0.42 (-0.90, 0.07); P = 0.09, I^2 = 48%
24 hrs	-0.27 (-0.55, 0.00); P = 0.05, I^2 = 46%	0.16 (-0.40, 0.72); P = 0.58, I^2 = 73%	-0.23 (-0.89, 0.43); P = 0.50, I^2 = 67%	-0.62 (-1.19, -0.06); P = 0.03, I^2 = 60%
48 hrs	-0.24 (-0.58, 0.10); P = 0.17, I^2 = 59%	0.09 (-0.53, 0.72); P = 0.77, I^2 = 78%	-0.17 (-0.50, 0.17); P = 0.33, I^2 = 0%	-0.56 (-0.89, -0.22); P = 0.001, I^2 = 0%
72 hrs	-0.58 (-0.83, -0.33); P < 0.001, I^2 = 64%	-0.22 (-0.48, 0.03); P = 0.09, I^2 = 0%		-0.70 (-1.10. -0.31); P < 0.001, I^2 = 0%
96 hrs	-0.15 (-0.49, 0.19); P = 0.40, I^2 = 0%	-0.08 (-0.34, 0.17); P = 0.51, I^2 = 0%		

Data presented as SMD (95% CI)

Table S3. Effect of individual polyphenol-rich foods, juices and concentrates on counter movement jump (CMJ)

**CMJ**	**Cherry**	**POM**	**Cocoa**	**Beetroot**
Immediately post	0.51 (-0.12, 1.14); P = 0.11, I^2 = 0%			0.25 (-0.08, 0.58); P = 0.13, I^2 = 0%
24 hrs	0.82 (-0.26, 1.90); P = 0.14, I^2 = 87%			0.34 (0.04, 0.65); P = 0.03, I^2 = 0%
48 hrs	0.72 (0.11, 1.32); P = 0.02, I^2 = 84%			0.46 (0.12, 0.81); P = 0.009, I^2 = 0%
72 hrs	0.65 (-0.55, 1.86); P = 0.29, I^2 = 87%			0.49 (0.07, 0.90); P = 0.02, I^2 = 22%

Data presented as SMD (95% CI)

Table S4. Subgroup Analyses comparing individual polyphenol-rich foods, juices and concentrates on maximal voluntary isometric contraction (MIVC)

**MIVC**	**Tart Cherry**	**Pomegranate**	**Beetroot**	**Cocoa**
Tart Cherry		Post (P = 0.30)<br>24 hrs (P = 0.31)<br>48 hrs (P = 0.14)<br>72 hrs (P = 0.48)<br>96 hrs (P = 0.50)	Post (P = 0.94)<br>24 hrs (P = 0.65)<br>48 hrs (P = 0.28)<br>72 hrs (P = 0.67)	24 hrs (P = 0.11)<br>48 hrs (P = 0.58)
Pomegranate			Post (P = 0.31)<br>24 hrs (P = 0.68)<br>48 hrs (P = 0.68)<br>72 hrs (P = 0.77)	24 hrs (P = 0.38)<br>48 hrs (P = 0.33)
Beetroot				24 hrs (P = 0.30)<br>48 hrs (P = 0.59)
Cocoa				

Table S5. Subgroup Analyses comparing individual polyphenol-rich foods, juices and concentrates on delayed onset muscle soreness (DOMS)

**DOMS**	**Tart Cherry**	**Pomegranate**	**Beetroot**	**Cocoa**
Tart Cherry		Post (P = 0.23)<br>24 hrs (P = 0.17)<br>48 hrs (P = 0.36)<br>72 hrs (P = 0.05)<br>96 hrs (P = 0.77)	Post (P = 0.80)<br>24 hrs (P = 0.28)<br>48 hrs (P = 0.19)<br>72 hrs (P = 0.61)	24 hrs (P = 0.91)<br>48 hrs (P = 0.76)
Pomegranate			Post (P = 0.10)<br>24 hrs (P = 0.05)<br>48 hrs (P = 0.07)<br>72 hrs (P = 0.05)	24 hrs (P = 0.38)<br>48 hrs (P = 0.47)
Beetroot				24 hrs (P = 0.38)<br>48 hrs (P = 0.10)
Cocoa				

Table S6. Subgroup Analyses comparing individual polyphenol-rich foods, juices and concentrates on countermovement jump (CMJ)

**CMJ**	**Tart Cherry**	**Pomegranate**	**Beetroot**	**Cocoa**
Tart Cherry			Post (P = 0.47)<br>24 hrs (P = 0.41)<br>48 hrs (P = 0.48)<br>72 hrs (P = 0.80)	
Pomegranate				
Beetroot				
Cocoa				
